# 16S rRNA and metagenomic shotgun sequencing data revealed consistent patterns of gut microbiome signature in pediatric ulcerative colitis

**DOI:** 10.1038/s41598-022-07995-7

**Published:** 2022-04-19

**Authors:** Wenxuan Zuo, Beibei Wang, Xin Bai, Yihui Luan, Yingying Fan, Sonia Michail, Fengzhu Sun

**Affiliations:** 1 Quantitative and Computational Biology Department, University of Southern California, Los Angeles, CA 90089 USA; 2 School of Mathematics, Shandong University, Jinan, 250100 Shandong China; 3 Data Sciences and Operations Department, Marshall School of Business, University of Southern California, Los Angeles, CA 90089 USA; 4 Department of Pediatrics, Keck School of Medicine of the University of Southern California, Los Angeles, CA 90033 USA

**Keywords:** Microbiology, Gastroenterology, Gastrointestinal diseases, Diseases, Gastrointestinal diseases

## Abstract

Dysbiosis of human gut microbiota has been reported in association with ulcerative colitis (UC) in both children and adults using either 16S rRNA gene or shotgun sequencing data. However, these studies used either 16S rRNA or metagenomic shotgun sequencing but not both. We sequenced feces samples from 19 pediatric UC and 23 healthy children ages between 7 to 21 years using both 16S rRNA and metagenomic shotgun sequencing. The samples were analyzed using three different types of data: 16S rRNA genus level abundance, microbial species and pathway abundance profiles. We demonstrated that (a) the alpha diversity of pediatric UC cases is lower than that of healthy controls; (b) the beta diversity within children with UC is more variable than within the healthy children; (c) several microbial families including *Akkermansiaceae, Clostridiaceae, Eggerthellaceae*, *Lachnospiraceae*, and *Oscillospiraceae*, contain species that are depleted in pediatric UC compared to controls; (d) a few associated species unique to pediatric UC, but not adult UC, were also identified, e.g. some species in the *Christensenellaceae* family were found to be depleted and some species in the *Enterobacteriaceae* family were found to be enriched in pediatric UC; and (e) both 16S rRNA and shotgun sequencing data can predict pediatric UC status with area under the receiver operating characteristic curve (AUROC) of close to 0.90 based on cross validation. We showed that 16S rRNA data yielded similar results as shotgun data in terms of alpha diversity, beta diversity, and prediction accuracy. Our study demonstrated that pediatric UC subjects harbor a dysbiotic and less diverse gut microbial population with distinct differences from healthy children. We also showed that 16S rRNA data yielded accurate disease prediction results in comparison to shotgun data, which can be more expensive and laborious. These conclusions were confirmed in an independent data set of 7 pediatric UC cases and 8 controls.

## Introduction

Ulcerative Colitis (UC) is a form of inflammatory bowel disease (IBD) that causes inflammation and ulcers in the digestive tract. Millions of individuals of all ages worldwide are affected by IBD. IBD is more common in developed countries with a prevalence reaching 0.3% and the prevalence of IBD increases as countries become more industrialized^41^. There is currently no cure for IBD and early diagnosis is essential for the management of the disease. Despite many years of extensive investigations, the exact etiology of UC remains unknown. Both genetic and environmental factors have been shown to be associated with the development of UC. The catalog of genome-wide association studies (GWAS)^27^ that archives GWAS for all different phenotypes recently lists 19 publications across 33 studies for UC in various populations and hundreds of genetic loci have been found to be associated with UC with p-value less than $10^{-8}$.

Some microbial organisms from human gut microbiota have been shown to be strongly associated with UC. Some investigators used marker genes, in particular, gut microbial 16S rRNA gene, to gauge the differences in bacterial compositions of UC cases and healthy controls^15,16,21,30–33,35,48^. It was consistently found that the compositions of bacterial organisms in UC, more broadly IBD cases, are significantly different from that of healthy controls. The gut microbiota of IBD cases is less diverse than that of healthy controls^16,30,33,35^. UC cases can be accurately predicted and differentiated from healthy controls using gut microbial profiles with the area under the receiver operating characteristic curve (AUROC) of 0.83 to 0.92 based on several machine learning algorithms in many studies^16,48^. Some bacterial taxa are strongly associated with UC with low frequencies of the phyla *Firmicutes* and *Bacteroidetes*, and high frequencies of the phyla *Proteobacteria* and *Actinobacteria*^48^.

In addition to the 16S marker gene, random shotgun metagenomic sequencing has also been used to investigate the association of gut microbial organisms and their pathways with IBD^13,41,46^. The metabolomic and shotgun metagenomic data can help understand the mechanisms by which human gut microbiota contribute to IBD and its development. It was shown that both metabolite and shotgun sequence data can predict disease status with high AUROC of 0.86 to 0.92^14^. Longitudinal multi-omics data including metagenomes, metatranscriptomes, proteomes, metabolomes, and viromes have also been generated and the relationships among the multi-omics data during disease development were discovered^18,20,26,41^. Associations between personal genomic variations and microbiota have also been identified^3^.

In a recent comparative study of the prediction accuracy of UC cases and healthy controls^45^ using personal genetic polymorphisms and microbiota, it was found that the aggregated AUROC using human gut data is 0.92, while the aggregated AUROC using personal genotypes is only 0.765, indicating much higher prediction accuracy using gut microbiota compared to personal genomic variation. The study also compared the prediction accuracies of phenotypes for many other complex traits including colorectal cancer, hypertension, type II diabetes, etc. using metagenomes and personal genomes, respectively, and showed that the prediction accuracy using metagenomes is generally much higher than that based on personal genomes.

Several groups investigated the association of microbial organisms with pediatric UC^11,23,26,28,41,42^. Shah et al.^42^ used only 10 pediatric UC cases and 13 controls to identify microbial taxa associated with pediatric UC using the 16S RNA gene, while Knoll et al.^23^ used only 5 UC children and their 12 healthy siblings with shotgun sequencing. De Meij et al.^11^ studied the gut microbiome of 41 pediatric UC cases and 61 healthy controls using the IS-pro technology that measures the genomic length between 16S and 23S rDNA. However, they did not use either 16S or shotgun sequencing in their studies. Malham et al.^28^ used 58 pediatric UC cases and 34 controls with 16S and 18S sequencing. However, no shotgun reads data were generated. The integrative Human Microbiome Project (iHMP)^26,41^ investigated the dynamics of both metagenomes and metatranscriptomes of IBD cases for both children and adults using 16S and shotgun sequencing of either ileum or rectum samples. The study included 15 pediatric UC cases and 14 non-IBD healthy controls. Except for iHMP^26^ with relatively small numbers of pediatric UC cases and healthy controls, no other studies are currently available to investigate pediatric UC cases and healthy controls using the abundance profiles of different taxa based on 16S rRNA marker gene, bacterial species abundance profiles, and pathway abundance from shotgun metagenomic reads data simultaneously. In this study, based on a set of 19 pediatric UC cases and 23 healthy controls with all of them having both 16S rRNA gene and metagenomic shotgun sequencing data available, we investigate the (1) alpha diversity, (2) beta diversity, (3) associations of different bacterial organisms with pediatric UC using 16S rRNA gene and metagenomic shotgun data from species to phylum level, and pathway abundance from metagenomic shotgun sequencing, respectively, and (4) prediction accuracy of pediatric UC disease status. We find consistently that (1) pediatric UC cases have lower alpha diversity than healthy controls, (2) the beta diversity between UC cases and between UC cases and controls is higher than that between healthy controls, (3) the set of taxa associated with pediatric UC cases have a high overlap with that associated with adult UC, and (4) pediatric UC status can be predicted with high accuracy with AUROC close to 0.90. These conclusions are validated in an independent set of 7 UC cases and 8 healthy controls.

## Materials and methods

### Data description

This study has been approved by the Children’s Hospital of Los Angeles Institutional Review Board (IRB # CCI-11-00148), and we confirm that all research was performed in accordance with relevant guidelines and regulations. Informed consent and/or assent was obtained from all participants and/or their legal guardians. To investigate the differences in microbial composition between pediatric UC cases and healthy controls, we collected fecal samples from 19 children with UC ages 7–21 years who have mild to moderate disease and 23 healthy controls. All the individuals were Caucasian and none of the subjects used antibiotics, probiotics or proton pump inhibitors. Three of the 19 UC cases used steroid. The numbers of UC cases using biologic, immunomodulator, and 5-aminosalicylate therapy at the time of study were 4, 2 and 2, respectively (supplementary Table S1 and S2). There are no age and gender differences between the UC cases and healthy controls (Table 1). Both the hypervariable V4 region of the bacterial 16S ribosomal RNA (rRNA) gene and the whole metagenome were sequenced on these individuals. However, the numbers of reads for the healthy controls are much higher than that for the UC cases for both the 16S rRNA and shotgun sequencing data (Table 1).

**Table 1:** Metadata demographics. There are no age and gender differences between the UC cases and healthy controls. However, the numbers of reads for the healthy controls are much higher than that for the UC cases for both the 16S rRNA and shotgun sequencing data. Statistical significance (p-value) for the differences was calculated using the Wilcoxon rank-sum test.

	Healthy	UC	p-value
Number of subjects	23	19	NA
Age (mean ± sd)	14.04±3.5	14.47±3.5	0.559
Gender (% male)	10 (43.48%)	10 (52.63%)	0.569
Number of reads (million) for 16S rRNA (mean ± sd)	0.19 ± 0.31	0.09 ± 0.06	0.002
Number of reads (million) for shotgun (mean ± sd)	4.30 ± 0.80	2.45± 2.17	0.0002

### Microbial DNA extraction and sequencing

Fecal DNA was extracted using the QIAamp Powerfecal DNA kit (Qiagen, Germantown MD) following the manufacturer’s instruction. Mechanical lysis of fecal cells were carried out using Vortex-Genie 2 with a horizontal tube holder adaptor.

#### The 16S rRNA amplicon sequencing

The 16S bacterial DNA V4 region was amplified from the extracted DNA by PCR and sequenced in the Illumina MiSeq Reagent Kit v2 flowcell on an Illumina MiSeq System using 2 $\times$ 150bp paired-end protocol. All samples were sequenced using uniquely barcoded primers (515FB: 5’-GTG YCA GCM GCC GCG GTA A-3’; 806RB: 5’-GGA CTA CNV GGG TWT CTA AT-3’)^47^ that were modified from the original 515F-806R primer pairs^9^. Agilent High Sensitivity DNA Bioanalyzer chips was used to assess the library qualities. The number of reads in each sample ranged from 22 thousand to 1 million. The mean number of reads for the samples was 145 thousand with standard deviation of 235 thousand. The histograms of the numbers of reads in the samples are given in supplementary Fig. S1(**A**).

#### Whole genome metagenomic sequencing and cleansing

Metagenomic libraries were constructed using the Nextera XT DNA Library Preparation Kit (Illumina) and Illumina Nextera XT Index v2 Kit A and B following the manufacturer’s protocols. Library qualities were assessed on Agilent High Sensitivity DNA Bioanalyzer chips. Libraries were pooled and sequenced on an Illumina NextSeq500 High Output v2 flowcell on an Illumina NextSeq 500 System, producing 2$\times$150bp paired-end reads. All samples and control reads were pre-processed, and quality filtered using Trim_Galore. Host-derived reads were removed using KneadData. The number of reads in each sample ranged from 400 thousand to 7,258 thousand. The mean number of reads of the samples was 3,465 thousand with standard deviation of 1,829 thousand. The histograms of the numbers of reads in the samples are given in supplementary Fig. S1(**B**).

#### The 16S rRNA and shotgun sequencing data processing

The 16S rRNA gene hypervariable V4 region sequencing data were processed and analyzed using QIIME2^6^. We first used DADA2^8^ to denoise the 16S sequences and then grouped the sequences into Amplicon Sequence Variants (ASVs). Taxonomic assignment was then performed for representative sequences from each ASV using a QIIME2 naive Bayesian classifier^5^ trained on Silva 138^38^ 99% OTUs from 515F/806R region of sequences. In total, 3,598 ASVs were reported, comprising 19 phyla, 28 classes, 66 orders, 122 families, 331 genera, and 814 species.

For the metagenomic shotgun reads data, after getting the cleansed data, Centrifuge^22^ and HUMAnN2 with its UniRef_90 EC database^13^ were then used to map the reads to known microbial genomes and gene families to obtain species-level abundance and pathway abundance, respectively. Default parameters of these packages were used in our study. The mapping rate of bacterial/archaeal for each sample was listed in supplementary Table S2. The distribution of bacteria/archaea mapping rate is shown in supplementary Fig. S2.

### Principal coordinate analysis (PCoA) and identification of factors contributing to beta diversity

We first calculated the beta diversity measured by the Bray–Curtis distance^7^ for each pair of samples. With the pairwise distance matrix of the samples, we then used Principal Coordinate Analysis (PCoA)^12^ to project the samples into two dimensional Euclidean space to visualize the clustering of the samples. PCoA is a dimension reduction method that projects samples into a low dimensional Euclidean space so that the pairwise distances of the samples in the Euclidean space are as close as possible to the given distances. The Goodness-Of-Fit (GOF) of PCoA indicates the accuracy that the Euclidean distances among the samples approximate the distance matrix. We used the function ‘pcoa’ from the R ‘ape’ toolbox^34^ for PCoA sample representation.

**Figure 1:**
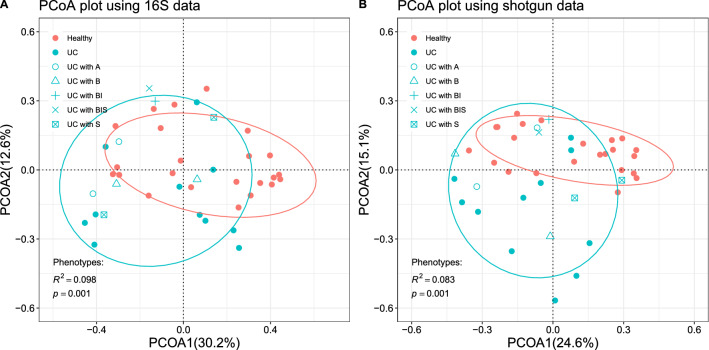
PCoA plots of the samples, with colors representing disease status and shapes representing therapies of UC patients (*A* patients taking 5-aminosalicylates, *B* patients taking biologic therapy, *I* patients taking immunomodulators, *S* patients using steroid, *BI* both B and I). (**A**) PCoA based on Bray–Curtis distance calculated from the 16S rRNA genus level abundance profiles. (**B**) PCoA based on the Bray–Curtis distance calculated from the bacterial species level abundance using the shotgun reads data.

The PCoA analysis clustered the samples into pediatric UC cases and healthy controls (Fig. 1) with good separation. We then used permutational multivariate analysis of variance (PERMANOVA)^2^ to quantify the separation of the samples into the two groups after adjusting for the age and gender as confounders.

We next investigated the joint contributions of disease status, age group and gender on beta diversity using multiple regression on matrices (MRM) as in^29^ implemented with the function ‘MRM’ in the ‘ecodist’ R package^17^. MRM performs a multiple regression of a response pairwise distance matrix of the samples on several explanatory distance matrices^25^. For this study, the response distance matrix was the beta diversity measured by the Bray–Curtis distances between the samples. Based on the disease status, the sample pairs could be divided into three categories (H, H), (H, D) and (D, D), with H corresponding to healthy controls and D corresponding to UC cases. To evaluate if the beta diversity values were different among the three categories, we defined dummy variables $(d_1, d_2)$ as (0, 0), (1, 0) and (0, 1) for (H, H), (H, D) and (D, D) pairs, respectively. We also similarly encoded samples based on gender denoted as $(g_1, g_2)$. With regards to age, we defined the dummy variable *a* with each element equals to the absolute value of the difference between the corresponding ages of the sample pairs. Let *B* denote the pairwise beta diversity between the samples measured by the Bray–Curtis distance. The MRM model was defined as

1 $$\begin{aligned} B= \beta _0 + \beta _1 d_1 + \beta _2 d_2 + \beta _3 a + \beta _4 g_1 + \beta _5 g_2 + \epsilon , \end{aligned}$$

where $(\beta _1, \cdots , \beta _5)$ are the coefficients for the different variables and $\epsilon$ is random error. The statistical significance levels of the MRM model and the regression coefficients being 0 are tested by permuting the beta diversity values while holding the explanatory variables constant.

Since sequence depths of the samples could potentially affect beta diversity, we rarefied the samples to the same number of reads and redid the same analyses as above. Similarly, different treatments of UC cases could affect the microbiome of the UC cases and in turn affect the beta diversity. Since the numbers of individuals with different treatments are small, we could not rigorously evaluate the effects of the different treatments on microbiome. To mitigate the potential confounding effects of the treatments on beta diversity analyses, we removed the UC cases with treatments and redid the same analyses as above.

### Identification of factors contributing to alpha diversity and microbial abundance in human gut microbial communities

We calculated the Shannon index^43^ as alpha diversity for each sample based on genus abundance from 16S rRNA gene and species abundance from metagenomic shotgun data.

It has been consistently found that the alpha diversity of a human gut microbial community is impacted by a variety of environmental factors such as ethnicity, age, gender, diet, and healthy status^16,30,33,35^. We investigated the joint contributions of UC disease status, age and gender on alpha diversity with both 16S rRNA sequence and shotgun sequencing data in our Caucasian samples using the following linear regression model as in^31^:

2 $$\begin{aligned} Y \sim \text {Disease Status} + \text {Age} + \text {Gender}, \end{aligned}$$

where *Y* is the log-transformed alpha diversity measured by the Shannon index^43^. The explanatory variables include disease-status, age, and gender.

Many studies identified statistical associations between the abundance levels of some microbial species or gene families with UC^16,30,31,48^. Following Thorsen et al.^44^, we assumed a negative binomial model for the count data and calculated normalization factors with trimmed mean of M values (TMM)^40^ using ‘calcNormFactors’ from the ‘edgeR’ package^39^, followed by common and tagwise dispersion estimations through the ‘estimateDisp’ function. Statistical tests were carried out using the ‘exactTest’ function.

The exact test yields a p-value for each variable. Multiple hypothesis tests were adjusted using the Benjamini and Hochberg false discovery rate (BH-FDR) procedure^4^. Significant associations were defined as those with BH-FDR below the threshold of 0.25.

It is well known that the number of reads analysed in a sample affects alpha diversity estimates. As shown in supplementary Fig. S3, the number of features increased with sequencing depth. Due to the vastly different total numbers of sequences per sample (supplementary Fig. S1), we randomly rarefied^19^ our samples at different rarefaction levels 100 times. If the number of reads in a sample is less than the rarefaction level, we directly use all the reads. This was done using the ‘feature-table rarefy’ command in QIIME2. We then calculated the alpha diversity for each sample.

### Prediction of pediatric UC disease status using microbial species or pathway abundance

We also used random forests^24^ implemented by the python package scikit-learn^36^ to predict the disease status (UC vs. healthy) of individuals. We set the number of trees at 100, 500 and 1000 and set other parameters at their default values in the random forests model since it was previously shown other parameters have minimal impacts on prediction accuracy^37^. Pathway abundance output by HUMAnN2 or trimmed mean of M-values (TMM) transformed abundance profile (genus-level abundance in 16S and species-level in shotgun data) was used as input features. We carried out 5-fold cross validation for prediction and repeated the process 50 times to evaluate the average performance. For each individual in the testing set, a score indicating the probability of having UC was obtained. We changed the threshold of this score, calculated the true positive rate (TPR) and false positive rate (FPR) under each threshold, and plotted the corresponding receiver operating characteristic (ROC) curve. Then the area under the ROC curve (AUROC) was used as a criterion measuring the prediction accuracy.

## Results

### PCoA plots show separation of samples based on disease status

We calculated the beta diversity measured by the Bray–Curtis distance^7^ between any pair of samples and used two-dimensional PCoA plots to visualize the samples based on genus abundance from 16S rRNA and species abundance from shotgun data, respectively. Figure 1 shows that the samples can be clustered into two groups according to their disease status.

We next used PERMANOVA^2^ to quantify the clustering of the samples into the two groups of pediatric UC cases and healthy controls after adjusting for age and gender information. Results for the full PERMANOVA analysis could be found in the supplementary Table S3. The resulting p-values for disease status are all less than 0.05, indicating clear separation of the samples. The $R^2$-values are 0.098 and 0.083 for the 16S rRNA gene and bacterial species, respectively, showing that the strengths of separation based on the disease status are similar.

To study the impact of sequence depth on beta diversity, we also showed the PCoA plots under different rarefaction levels based on the 16S rRNA and shotgun sequencing data in supplementary Figs. S4 and S5, respectively. The distributions of samples under different rarefaction levels remain similar. Although the p-value changes slightly, the separation between the UC cases and controls is still clear.

To assess the overall consistency of the PCoA plots between the 16S rRNA and metagenomic shotgun data, we calculated the Spearman’s correlation between the first principal coordinate (PCoA1) of the 16S genus data and that of the shotgun species data based on Bray–Curtis distance. As shown in supplementary Fig. S6, we found a highly significant correlation between amplicon genus abundance PCoA1 and shotgun species abundance PCoA1 (the Spearman’s correlation $\rho = 0.881$, p-value$<2.2\times 10^{-16}$). Correlation coefficients calculated separately for UC cases and healthy controls are similar to the overall correlation coefficient and show no significant difference between each other (the Spearman’s correlation $\rho = 0.943$, p-value$<2.9\times 10^{-6}$ for healthy controls; the Spearman’s correlation $\rho = 0.742$, p-value$=4.1\times 10^{-4}$ for UC cases).

To investigate the potential confounding effects of treatments for some of the UC cases on the PCoA results, we then removed the UC cases with treatments. The resulting PCoA plots are shown in supplementary Fig. S7. To mitigate the potential effects of sequencing depth on beta diversity, we then rarefied the samples to the same sequencing depth at different levels and the corresponding PCoA plots are shown in supplementary Figs. S8 and S9 for the 16S rRNA and shotgun sequencing data, respectively. The PCoA plots based on all the data are highly similar to that by rarefaction and removing the UC cases with treatments. The first principal coordinate (PCoA1) using the 16S rRNA data continues to be highly correlated with that using the shotgun data as shown in supplementary Fig. S10.

### Disease status contributes significantly to beta diversity

We used multiple regression on matrices (MRM)^29^ to further investigate the relative contributions of disease status, age and gender to beta diversity between samples using equation 1. Based on the shotgun data, the MRM model 1 explains a significant proportion ($R^2 = 12.1\%$; p-value $= 0.011$) of the variability of beta diversity between the samples. Table 2 shows the coefficients and the corresponding statistical significance (p-value) for the dummy variables related to disease status $(d_1, d_2)$, age *a*, and gender $(g_1, g_2)$. The two significant coefficients $\beta _1 = 0.249$ and $\beta _2 = 0.189$ are for the two dummy variables related to disease status indicating its most important contributions to beta diversity. The results show that the beta diversity between UC samples or between UC and healthy samples is much higher than that between health samples.

The corresponding results using 16S genus abundance are similar to that using shotgun data. But the disease status $d_1$ is not significant in this result. Based on the 16S data, the MRM (Table 2) model including all three variables explained a proportion ($R^2 = 4.4\%$; p-value $= 0.121$) for the genus-level abundance.

**Table 2:** Coefficients of variables for multiple regression on matrices. The coefficient for disease status $d_1$ indicates the difference of beta diversity between (D, H) samples versus (H, H) samples and the coefficient for disease status $d_2$ indicates the beta diversity difference between (D, D) samples and (H, H) samples, where *D* and *H* represent UC and healthy individuals, respectively. The coefficient for age *a* measures the contribution of age to beta diversity. The coefficient for gender $g_1$ measures the beta diversity difference between (M, F) individuals versus (M, M) individuals. Similarly, the coefficient for gender $g_2$ measures the beta diversity difference between (F, F) individuals versus (M, M) individuals, where *M* and *F* represent male and female, respectively. If a partial regression coefficient is reported, its significance level is < 0.05. *P $\le$ 0.01 and **P $\le$ 0.001.

	16S genus level abundance	Species level abundance
Disease status $d_1$	Not significant	0.249(*)
Disease status $d_2$	0.090	0.189(**)
Age group *a*	Not significant	Not significant
Gender $g_1$	Not significant	Not significant
Gender $g_2$	Not significant	Not significant

Since sequencing depth and UC treatments do not markedly affect beta diversity, we did not study the contributing factors for beta diversity by rarefaction and removing UC cases with treatments.

### Gut microbiota of pediatric UC cases is less diverse than that of healthy controls

We next investigated the joint effects of disease status, age and gender on alpha diversity of the gut samples using equation 2. The coefficients and the corresponding significance using the 16S rRNA gene and bacterial species abundance profiles are given in Table 3. For the 16S rRNA gene and shotgun sequencing data, disease status contributes significantly to alpha diversity. However, age and gender do not significantly affect alpha diversity using both 16S rRNA gene and shotgun reads data.

**Table 3:** Joint contributions of disease status, age and gender on alpha diversity. Coefficients and statistical significance of the factors are quantified by a linear model 2. If a regression coefficient is reported, its significance level (t test) is < 0.05. *P $\le$ 0.01 and  **P $\le$ 0.001.

	16S genus level abundance	Species level abundance
Disease status	− 0.223 (**)	− 0.140 (*)
Age	Not significant	Not significant
Gender	Not significant	Not significant

**Figure 2:**
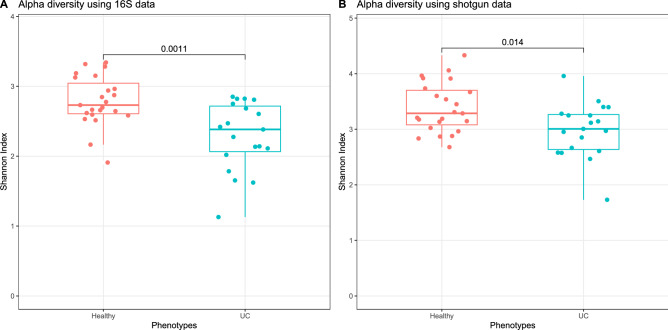
Box plots of Shannon indices for gut samples of pediatric UC cases and healthy controls stratified by disease status. P-values were calculated using the Wilcoxon rank sum test. (**A**) Shannon indices were calculated based on the 16S genus level abundance. (**B**) Shannon indices were calculated using the bacterial species abundance based on shotgun reads data.

To visually investigate the differences of alpha diversity between pediatric UC cases and heathy controls, we divided the samples into two groups based on their health status (UC and control) and then compared the alpha diversity of the gut samples within the two groups using the Wilcoxon rank sum test since age and gender were not significantly associated with alpha diversity. Figure 2 shows the boxplots of the Shannon indices for the two groups together with the resulting p-value based on the 16S rRNA gene and bacterial species abundance. Based on the 16S rRNA gene data, the alpha diversities of healthy individuals are significantly higher than that of pediatric UC patients with p-value of $1.1\times 10^{-3}$, which is consistent with previous studies.

With shotgun sequencing data, we can also observe the alpha diversity differences between pediatric UC cases and healthy controls with p-value of $1.4\times 10^{-2}$ based on bacterial species abundance. This p-value is higher than the corresponding p-value based on the 16S rRNA data. This result indicates that the differences in alpha diversity of the gut of UC cases compared to that of healthy controls based on 16S rRNA gene is more pronounced than that based on the shotgun data.

Then we calculated the correlation between the Shannon indices of the samples based on the 16S rRNA genus data and shotgun metagenomic species data. We found a significant correlation between amplicon genus Shannon indicies and that of shotgun species abundance Shannon indicies (the Spearman’s correlation $\rho = 0.633$, p-value$=1.1\times 10^{-5}$) (supplementary Fig. S11).

We next investigated the effects of sequencing depth on the alpha diversity results by rarefying the samples to the same number of reads. The results are presented as supplementary Figs. S12 and S13 based on the 16S rRNA and shotgun metagenomic reads, respectively. To mitigate the potential treatment effects on alpha diversity, we redid the analyses by removing the UC samples with treatments and the results are presented as supplementary Figs. S14 and S15 based on the 16S rRNA and shotgun reads, respectively. These results show that the sequencing depth differences and UC treatments do not change our results on alpha diversity. After removing UC cases with treatments, the alpha diversity using the 16S rRNA data and the alpha diversity using the shotgun data continue to be highly correlated as shown in supplementary Fig. S16.

### Taxa differentially abundant between pediatric UC cases and controls

We next used the exact test from ‘edgeR’ package to identify taxa associated with pediatric UC. The tested taxa were from the species to the phylum levels and the taxa with Benjamini-Hochberg false discovery rate (BH-FDR) less than 0.25 are shown in supplementary Tables S4-S6. Following the procedures from^26^, taxa with very low variance (below half the median of all feature-wise variances) or with $\ge 90\%$ zero values were removed in this analysis.

**Figure 3:**
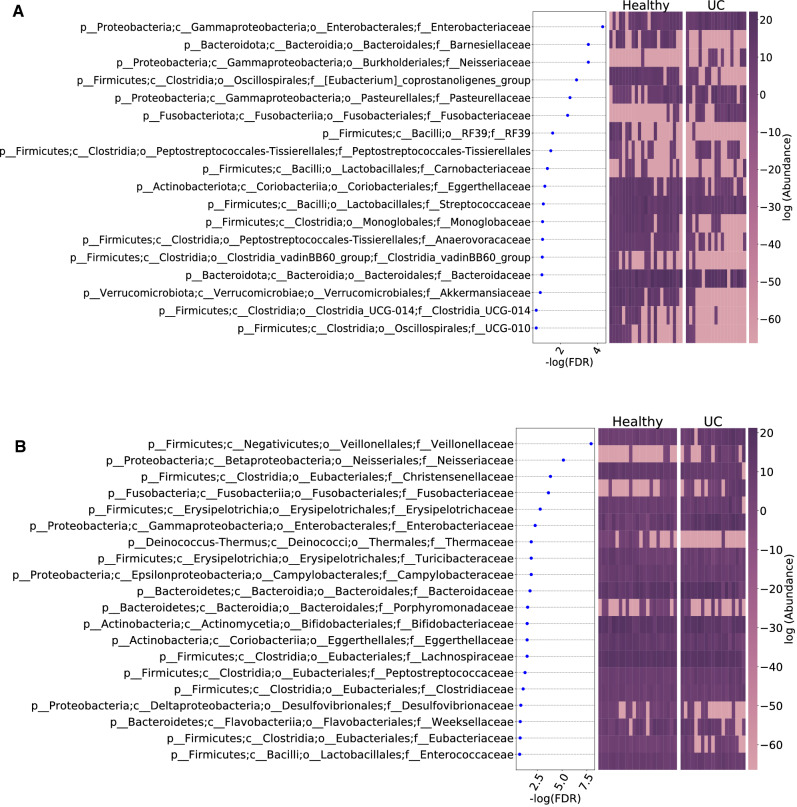
Bacterial families associated with pediatric UC ranked according to their statistical significance and heatmap using the (**A**) 16S, (**B**) shotgun profiling data. Each row represents a bacteria family. The left panel is dot plot of negative log10 transformed FDR value and the right two panels are heatmaps of abundance with log transformation for healthy controls and UC cases. Purple indicates high abundance, while pink indicates low abundance.

Eighteen and 24 microbial families were identified as associated with pediatric UC based on the 16S data and shotgun data, respectively. The corresponding $-\log (\text {FDR})$ and the distribution of the relative abundance levels of the associated families in the pediatric UC cases and controls are shown in Fig. 3 A. Similar results based on the shotgun reads data are given in Fig. 3 B. In addition, we also identified the microbial species associated with pediatric UC. Sixty and 68 species were identified as associated with UC using 16S and shotgun sequencing data, respectively.

**Figure 4:**
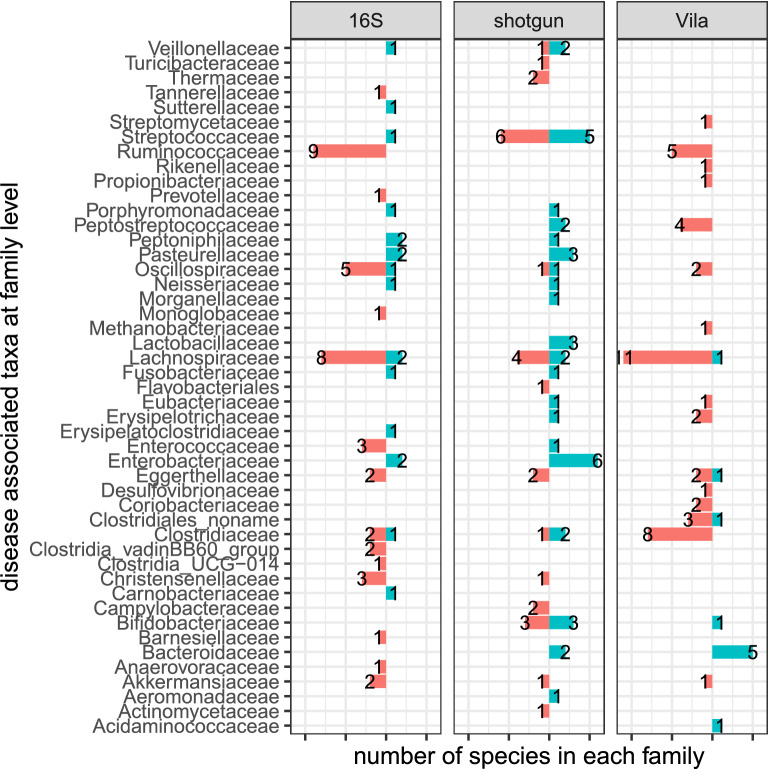
Comparison of UC associated species using 16S rRNA gene and shotgun reads data with the associated species from Vila et al.^46^. For each microbial family, the numbers of species that were increased (green) or decreased (red) are shown.

Although UC children and UC adults may have different associated microbial species, we investigated if they share some common differentially abundant species by comparing our findings with the result of Vila et al.^46^ which studied the association of microbial composition with IBD including both CD and UC and irritable bowel syndrome (IBS) in adults. The study contained 126 UC cases and 1025 controls. Figure 4 shows the comparison of associated species from^46^ and from our study using the 16S and shotgun sequencing data. The figure clearly shows that most of the associated species are depleted in UC cases versus healthy controls for both children and adults, consistent with the observation of low diversity in the gut of UC cases compared to that of healthy controls. Some species from five families: *Akkermansiaceae, Clostridiaceae, Eggerthellaceae, Lachnospiraceae,* and *Oscillospiraceae*, are found to be depleted in UC cases based on all three data sets. In addition to these five families, we also found that *Bacteroidaceae* and *Bifidobacteriaceae* contain species enriched in UC cases in Vila et al.^46^, and *Ruminococcaceae* contains depleted species in UC cases in Vila et al.^46^, and were confirmed by either the 16S or the shotgun sequencing data. Supplementary Table S7 shows details of the differentially abundance species from Vila et al.^46^, the shotgun data and 16S data from this study, respectively. These results clearly show that there are marked overlaps between differentially abundant species for pediatric UC and adult UC cases.

To see the effects of UC treatments on the identified differentially abundant taxa, we removed the UC samples with treatments and redid the same analyses. The results are shown in supplementary Fig. S17. After removing the UC cases with treatments, 27 and 28 microbial families were identified as associated with pediatric UC based on the 16S rRNA data and shotgun data, respectively. Similarly, species from *Akkermansiaceae*, *Clostridiaceae*, *Eggerthellaceae*, *Lachnospiraceae*, and *Oscillospiraceae*, are still found to be depleted in UC cases using both 16S rRNA data and shotgun data. After removing the UC cases with treatments, species from the *Sutterellaceae*, *Peptoniphilaceae*, and *Erysipelatoclostridiaceae* families were no longer associated with UC using 16S rRNA data. In contrast, species from *Firmicutes RF39* and *Hungateiclostridiaceae* were found to be decreased in UC cases without treatments using 16S data, indicating the potential influence of the treatments. Besides, we also identified species in *Acidaminococcaceae*, *Firmicutes noname*, and *Synergistaceae* that were depleted in UC cases without treatments using shotgun data. Two species from *Bacteroidaceae* were found to be increased in UC cases when concentrated on the patients without treatments.

**Figure 5:**
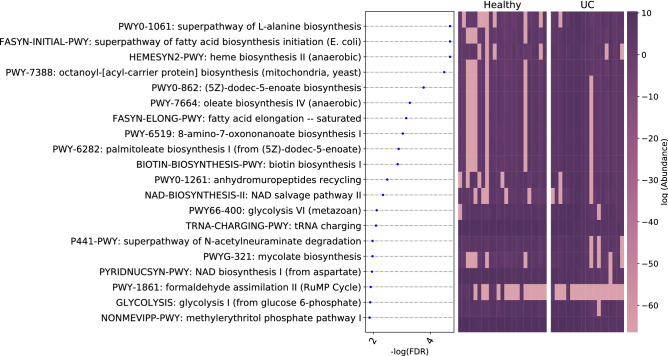
Pathways associated with pediatric UC ranked according to their statistical significance and heatmap using the shotgun pathway abundance.

The differentially abundant pathways are shown in Fig. 5. L-alanine biosynthesis, heme biosynthesis and oleate biosynthesis are enriched in UC samples. Constante et al.^10^ showed that heme significantly changed the microbial composition of mice, characterized by a decrease in alpha diversity, a reduction of *Firmicutes* and an increase of *Proteobacteria*, which is consistent with our results of family-level differential analysis. Moreover, Wiese et al.^49^ discovered that UC subjects had increased oleic acid intake, which could alter the inflammation severity. Conversely, the results (Table S6) show that tRNA charging and formaldehyde assimilation pathways are decreased in UC.

### Pediatric UC disease status can be accurately predicted based on either 16S or shotgun sequencing data

We finally used microbial relative abundance profiles from both 16S rRNA gene and shotgun reads data as well as pathway abundance profiles to predict UC disease status using random forests. Table 4 shows the average AUROC together with the 95% confidence intervals with 5-fold cross-validation using 16S genus-level abundance, shotgun species-level abundance and pathway abundance, respectively, based on 500 trees. The average AUROC scores are all higher than 0.90, indicating that the UC status can be accurately predicted using either the 16S or shotgun data. We also added covariates such as gender and age in the random forests models. It can be seen that age and gender do not markedly impact the AUROC score.

**Table 4:** The prediction performance of random forests algorithm measured by AUROC and its 95% confidence interval based on the genus level abundance profiles of 16S rRNA gene, microbial species and pathway abundance from shotgun sequencing data.

Type of abundance	AUROC	95% CI	Age involved	Gender involved
16S genus level abundance	0.908	(0.810,1)	FALSE	FALSE
	0.929	(0.843,1)	TRUE	TRUE
Shotgun species-level abundance	0.910	(0.813, 1)	FALSE	FALSE
	0.903	(0.802, 1)	TRUE	TRUE
Shotgun pathway abundance	0.951	(0.878, 1)	FALSE	FALSE
	0.964	(0.902, 1)	TRUE	TRUE

The AUROC scores based on 100 and 1000 trees in the random forests model are given in supplementary tables S8 and S9, respectively. Compared to the classification model with 500 trees, the AUROC score from the random forest model with 100 trees has higher variance. The AUROC scores from random forests models with 100, 500 and 1000 trees do not have significant differences (p-values of the Mann-Whitney U test for different types of abundance profiles are all greater than 0.05 as shown in supplementary Table S10). For computational efficiency and AUROC stability, we used 500 trees in our final models.

We did the same analyses by removing UC samples with treatments and the results are given in supplementary Table S11. All the AUROC values are slightly higher compared to those including UC cases with treatments. The results suggest that some of the treatments may affect the microbiome.

### Validation using an independent set of pediatric UC cases and healthy controls

**Figure 6:**
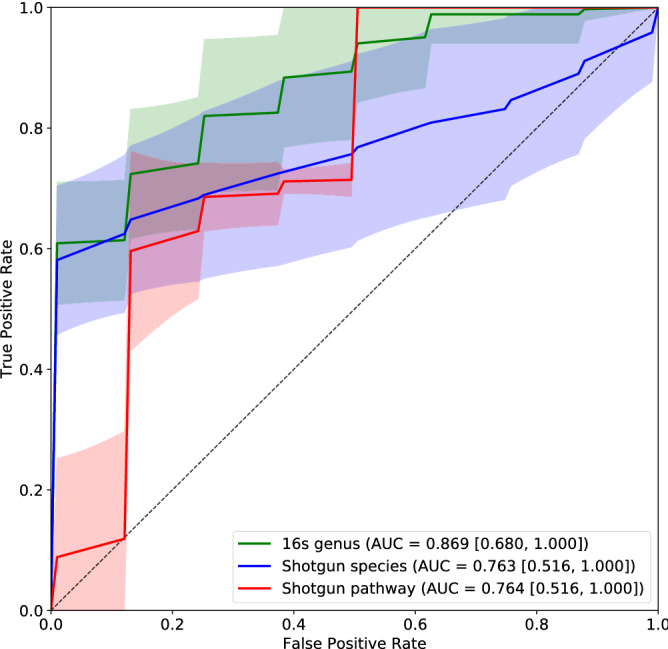
Validation ROC curves based on the random forests models with 500 trees developed from the training data. Numbers in the square brackets represent the $95\%$ confidence interval of the AUROC score.

We validated the above results using an independent set of 7 pediatric cases ages from 7 to 17 years old and 8 healthy controls who were not used in the above analyses. However, all the healthy controls were adults of ages 21 or older. Figures S18 and S19 in the supplementary material show the PCoA plots based on the Bray–Curtis dissimilarity and the alpha diversity values in the cases and controls, respectively. Figure S18 shows a similar clustering pattern of the cases and controls as in Fig. 1. Figure S19 confirms that the alpha diversity in the cases is lower than that in the controls with all the p-values less than 0.10 based on two types of data. The relatively higher p-values compared to that in Fig. 2 can be attributed to the small sample sizes in the validation data set. The ROC curves based on the prediction results of models with 500 trees trained using the data given above are given in Fig. 6. Since gender and age do not contribute much in the model, we excluded these two factors in this part. The corresponding AUROC score based on 16S rRNA data is the highest, which is 0.869. The performance on the independent validation data set is not as good as the result of cross validation. This is partially due to the different distribution of sequence depths between the training set and the independent validation set.

The validation results based on the random forests models with 100 and 1000 trees are given in supplementary Fig. S20. These results further validate our conclusions on the training set. The ROC curves of the random forests model with 100 trees have much wider error band compared to those with 500 and 1000 trees, suggesting higher variance of the AUROC scores based on the 100 trees model. Supplementary Table S12 shows the p-values comparing the AUROC scores from the random forests models with 100, 500 and 1000 trees and the results show significant differences between the AUROCs with 100 trees and the AUROCs with 500 or 1000 trees. The ROC curves from random forests models with 500 and 1000 trees show a similar trend and have roughly the same width of error brand, indicating that there is not significant difference between the results of these two models (p-values of the Mann–Whitney U two-tailed test for all three types of abundance are greater than 0.05).

The 8 healthy controls are not ideal as they are older than the 7 pediatric cases in the validation set. The 8 healthy controls were chosen because we did not have additional metagenomic data from healthy children available. To see if the microbiota of the 8 healthy controls is significantly different from that of healthy children, we present the PCoA plots of the 8 healthy controls and 23 pediatric healthy controls based on 16S rRNA species profiles as shown in supplementary Fig. S21. The p-values corresponding to testing if the two groups are separated using PERMANOVA are also included in the figure. Figure S19 shows that the 8 adult and 23 pediatric healthy controls mix with each other well and all the p-values are higher than 0.2. The box plots of the alpha diversity values within the two groups are given in Fig. S21 and the corresponding p-values comparing the distribution of diversity values between the two groups using the Kolmogorov–Smirnov test are also given. Both figures S21 and S22 show that the microbiota of the adults and that of children are not significantly different, at least in our data set.

## Discussion

In this study, using both 16S rRNA gene and shotgun sequencing data, we investigated the differences between gut microbiome of 19 pediatric UC cases and 23 age-matched healthy controls. Our study has several novelties compared to previous pediatric UC studies^11,23,26,28,41,42^. First, compared to the studies^11,23,28,42^ that used 16S, 18S or the 16S-23S inter-space region, we used both 16S and shotgun sequencing, which makes it possible to compare the results from these two types of data. Second, compared to the iHMP study^26^ that investigated 15 pediatric UC cases and 14 healthy controls, the sample sizes of our study are relatively larger. To the best of our knowledge, our study has the largest sample size among pediatric UC studies involving both 16S and shotgun sequencing although the sample sizes of our study are still relatively small. In addition, the fecal samples of the UC cases in the iHMP study were collected without considering whether UC was active. On the other hand, the UC was active at the time of fecal sample collection in our study.

Several interesting results were obtained based on the 16S RNA gene, and the microbial species and pathway abundance profiles based on shotgun data. First, we confirmed previous results that the gut microbiota of pediatric UC cases is less diverse than that of healthy controls based on Shannon index. Second, principal coordinate analyses (PCoA) based on the two types of abundance profiles showed similar patterns of separation of pediatric UC cases from healthy controls. Third, several bacterial taxa were identified in association with pediatric UC. Comparing our results to those reported by Vila et al.^46^, we see marked overlap of differentially abundant bacterial families for both pediatric and adult UC cases including *Akkermansiaceae, Clostridiaceae, Eggerthellaceae, Lachnospiraceae*, and *Oscillospiraceae*. Some differentially abundant taxa unique to either pediatric or adult UC were also found. For example, 3 species from the *Bifidobacteriaceae* family were found to be depleted in UC children using our shotgun data, which is consistent with^1^ that showed the *Bifidobacteriaceae* family was depleted in adult UC. However, only one species from this family was found to be enriched in adult UC in^46^.

Finally, we applied random forests to predict pediatric UC cases and controls with 5-fold cross validation AUROC scores close to 0.9 based on bacterial taxa abundance using either 16S rRNA gene or shotgun data. Unlike in previous studies, we did not find that age or gender played important roles in any of these results. One potential explanation is that the age range of 7–21 within our cohort is narrow resulting in no association of the gut microbiome with age. Within this age range, gender probably does not affect the gut microbiome either. One surprising finding from our study is that shotgun data does not provide much more accurate information in terms of predicting pediatric UC cases from controls and differences in alpha and beta diversity between pediatric UC cases and controls. Obviously, shotgun data can provide differentially abundant pathway information that 16S cannot.

A small number of the pediatric UC cases were under several treatments. Due to the small number of individuals under treatments, a rigorous statistical analysis on the impacts of the treatments on human gut could not be carried out. Therefore, we analyzed the data with and without the UC cases who were under treatments separately. The results from our study were approximately similar.

In this study, we focused on comparative studies of pediatric UC cases versus healthy controls using 16S rRNA gene and shotgun sequencing data. Therefore, we only mapped the reads from shotgun data to bacterial genomes. Shotgun data provide information on other molecules within human gut such as viruses, plasmids, etc, that were not investigated in this study. Reads that cannot be mapped to known bacterial genomes were also not considered. These are topics for further studies.

## Conclusions

In this study we demonstrated that pediatric ulcerative colitis subjects harbor a dysbiotic and less diverse gut microbial population with distinct differences from healthy children. We also show in this population, 16S rRNA data yield accurate prediction results in comparison to shotgun data, which can be more expensive and laborious.

## Supplementary Information

Supplementary Information 1.

Supplementary Information 2.

Supplementary Information 3.

## Data Availability

The dataset presented in this study has been deposited at NCBI SRA under Accession ID PRJNA759642. Code used to analyze data and generate figures can be obtained from https://github.com/zuowx/IBD_analysis and https://github.com/wbb121/IBD-data-analysis.
