# Supplementary material for: 16S rRNA and metagenomic shotgun sequencing data revealed consistent patterns of gut microbiome signature in pediatric ulcerative colitis
Source: Sci Rep. 2022 Apr 19;12:6421. doi: 10.1038/s41598-022-07995-7 (PMC9018687; doi:10.1038/s41598-022-07995-7)
Supplement: Supplementary file 2 — Supplementary Information 2. [file 41598_2022_7995_MOESM2_ESM.pdf]

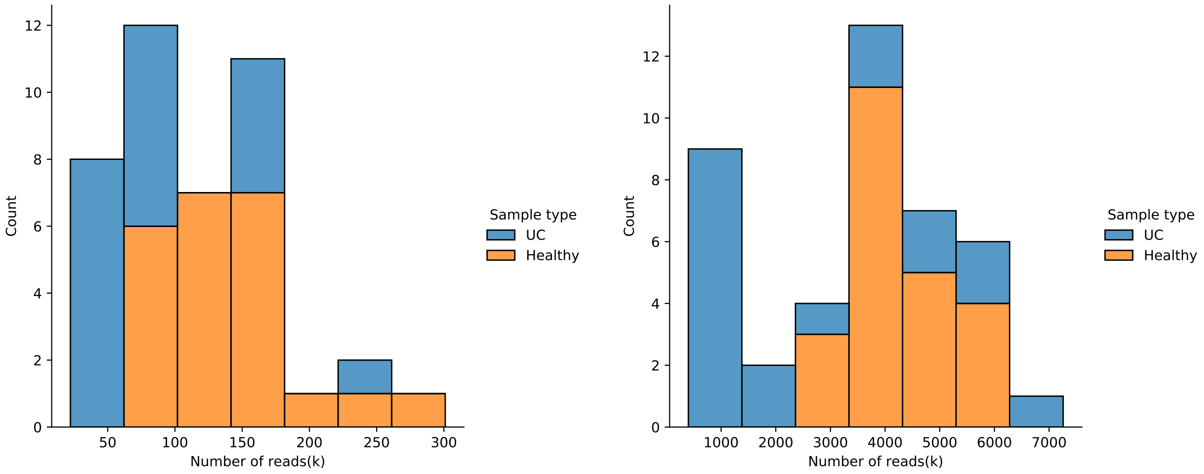

A **B**

Figure S1: Histograms of the numbers of reads in the samples for 16S rRNA gene (**A**) and metagenomic shotgun data (**B**).

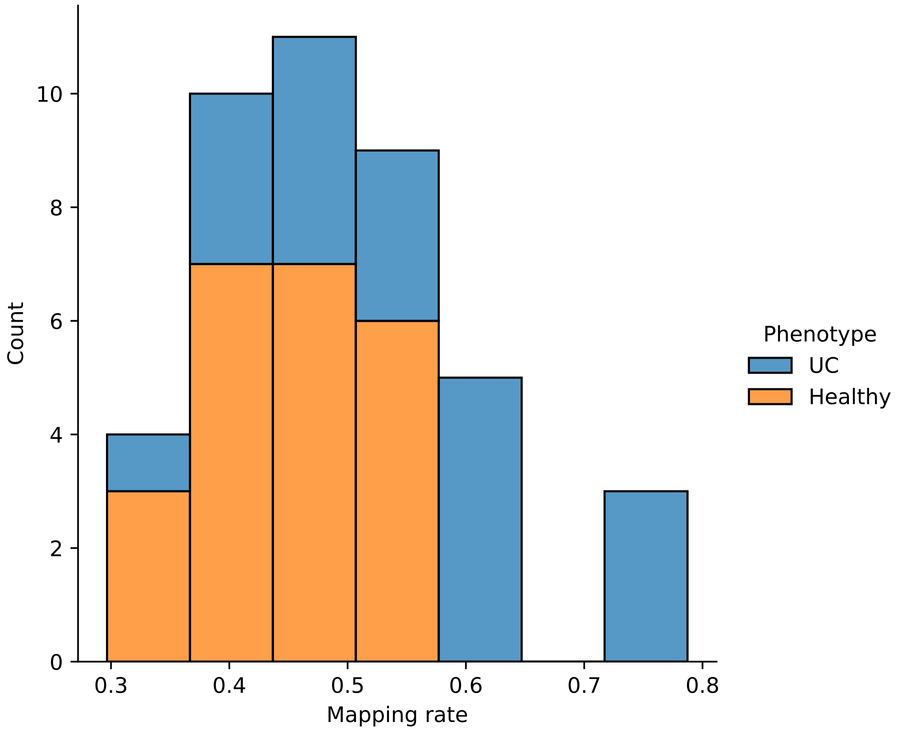

Figure S2: The distribution of mapping rates for the metagenomic shotgun data.

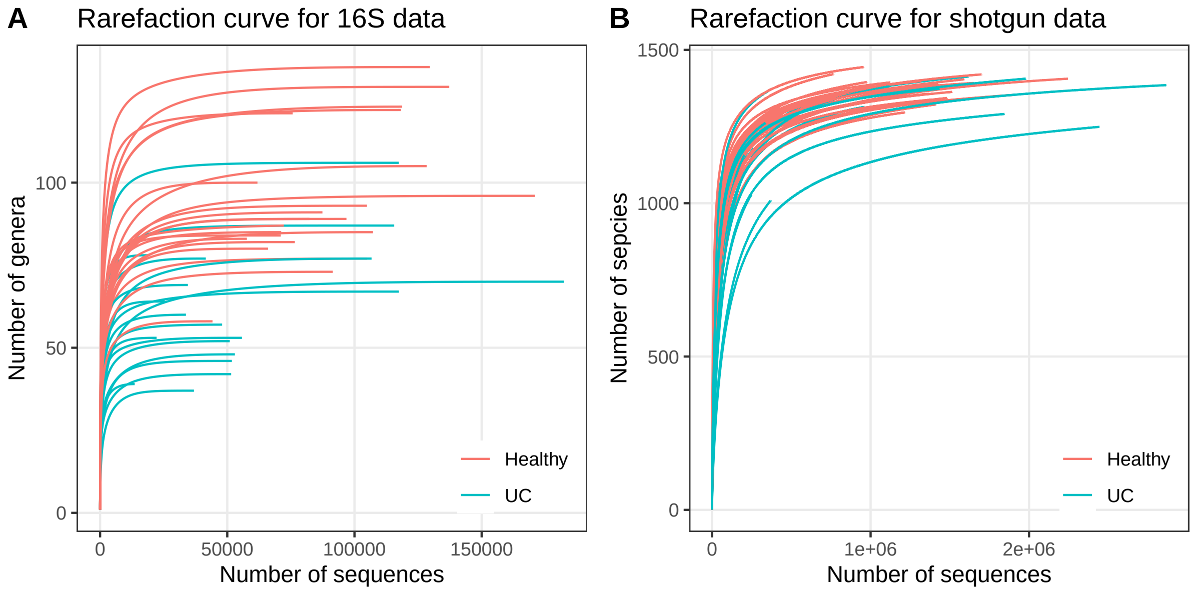

Figure S3: Rarefaction curves for (**A**) the 16S rRNA and (**B**) the shotgun sequence data.

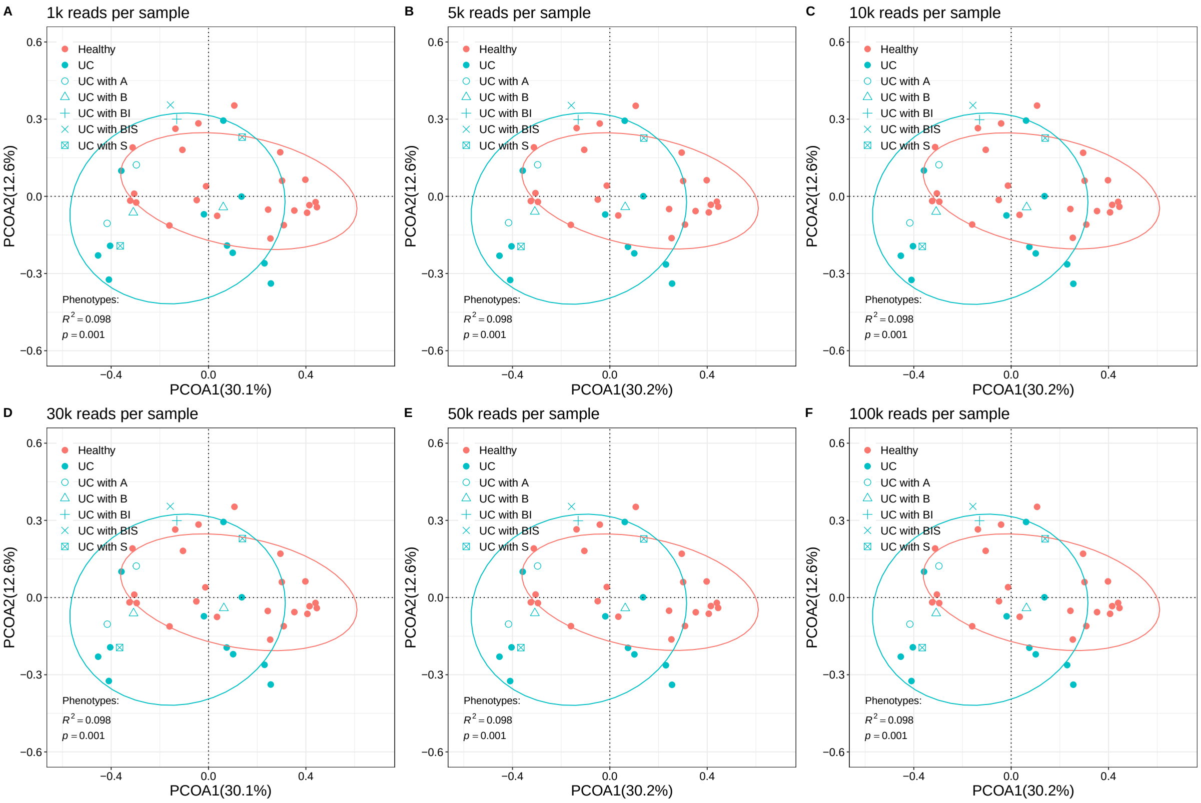

Figure S4: PCoA plots of the samples at different rarefaction levels using genus abundance from the 16S rRNA data, with colors representing disease status and shapes representing therapies of UC patients (A, patients taking 5-aminosalicylates; B, patients taking biologic therapy; I, patients taking immunomodulators; S, patients using steroid). The p-values for the effect of phenotypes after removing the influence of age and gender were calculated using PERMANOVA.

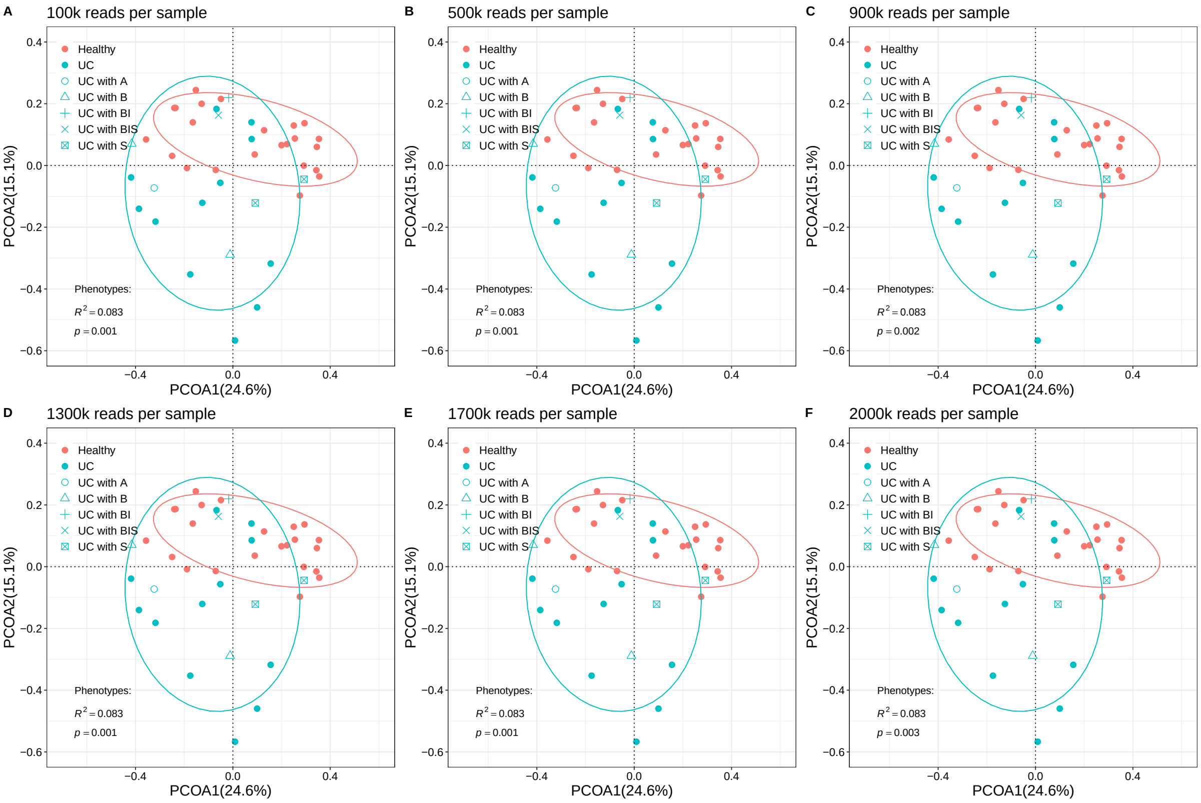

Figure S5: PCoA plots of the samples at different rarefaction levels using species abundance from the metagenomic shotgun data, with colors representing disease status and shapes representing therapies of UC patients (A, patients taking 5-aminosalicylates; B, patients taking biologic therapy; I, patients taking immunomodulators; S, patients using steroid.). The p-values for the effect of phenotypes after removing the influence of age and gender were calculated using PERMANOVA.

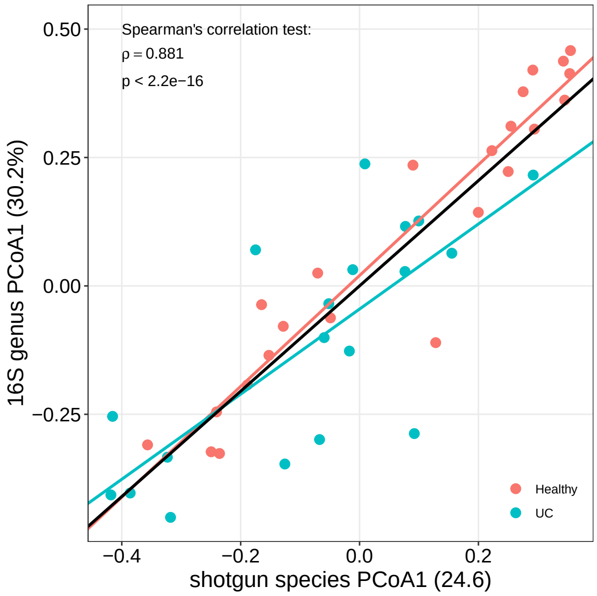

Figure S6: Correlation between the first principal coordinate (PCoA1) based on the 16S genus data and that of the metagenomic shotgun species data using the Bray-Curtis distance. Green, red, and black lines represent linear trends for the UC, healthy, and overall groups, respectively. The Spearman's correlation coefficient and p-value were also included.

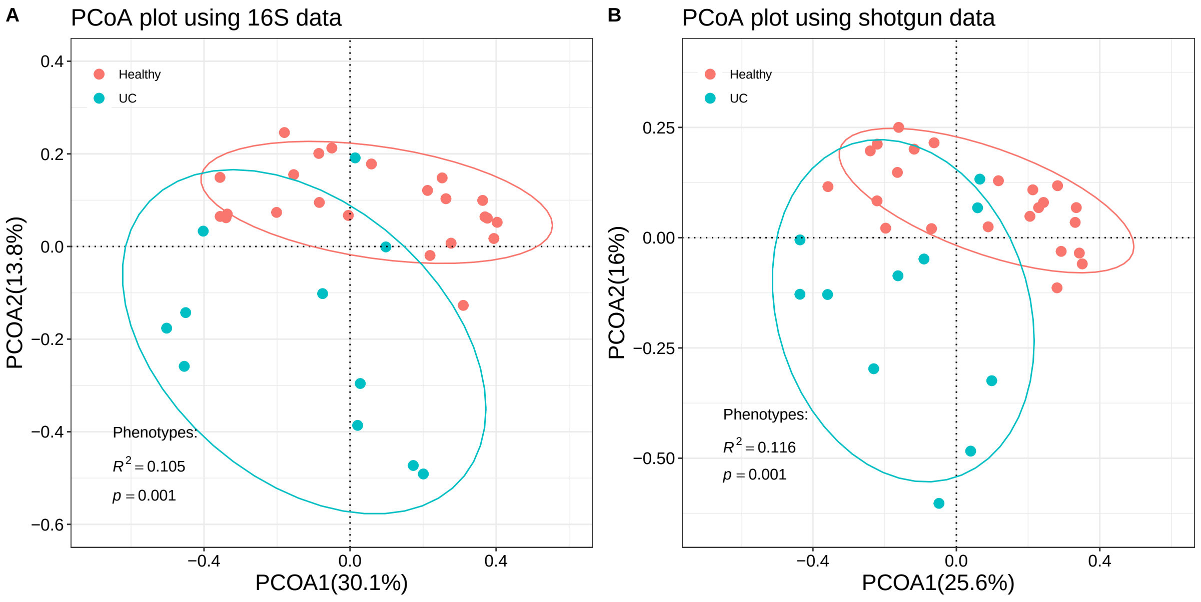

Figure S7: PCoA plots of the samples, with colors representing disease status. The p-values for the effect of phenotypes after removing the influence of age and gender were calculated using PERMANOVA. (**A**) PCoA based on the Bray-Curtis distance calculated from the 16S rRNA genus level abundance profiles. (**B**) PCoA based on the Bray-Curtis distance calculated from the bacterial species level abundance using the shotgun reads data. UC cases with treatments were removed in this figure.

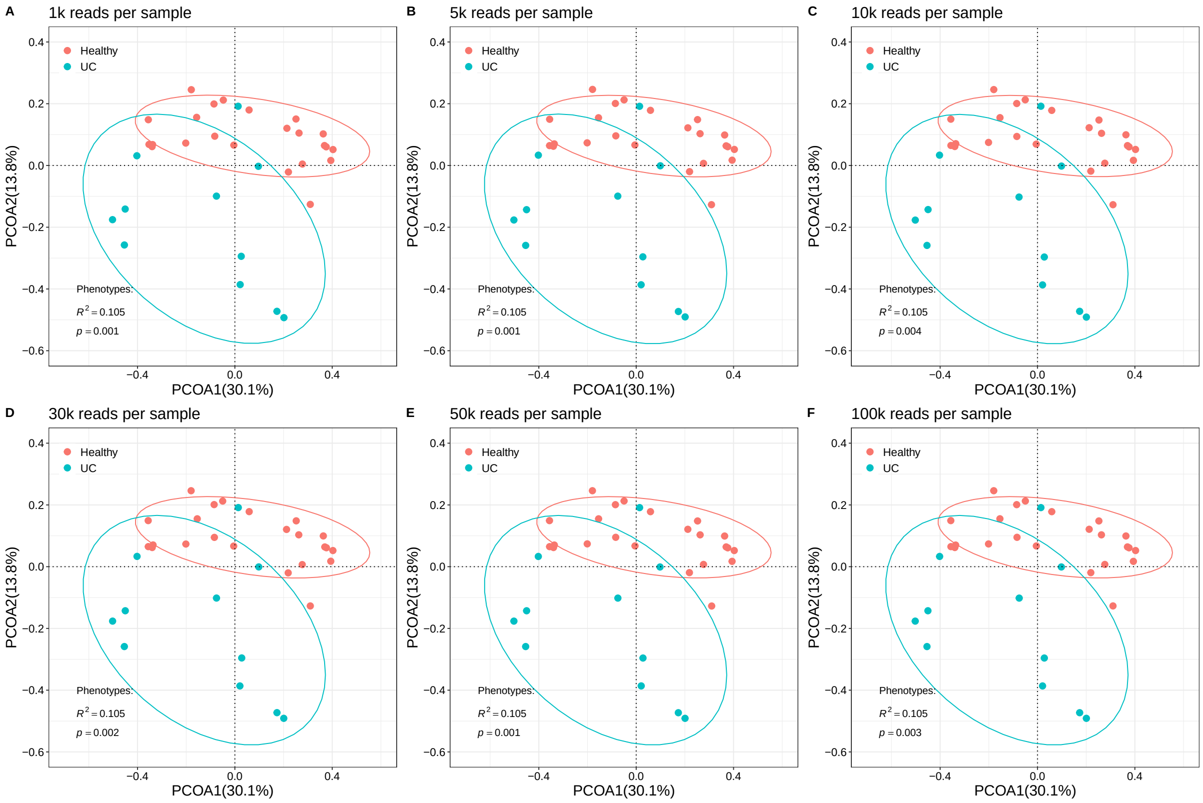

Figure S8: PCoA plots of the samples at different rarefaction levels using genus abundance from the 16S rRNA data, with colors representing disease status. The p-values for the effect of phenotypes after removing the influence of age and gender were calculated using PERMANOVA. UC cases with treatments were removed in this figure.

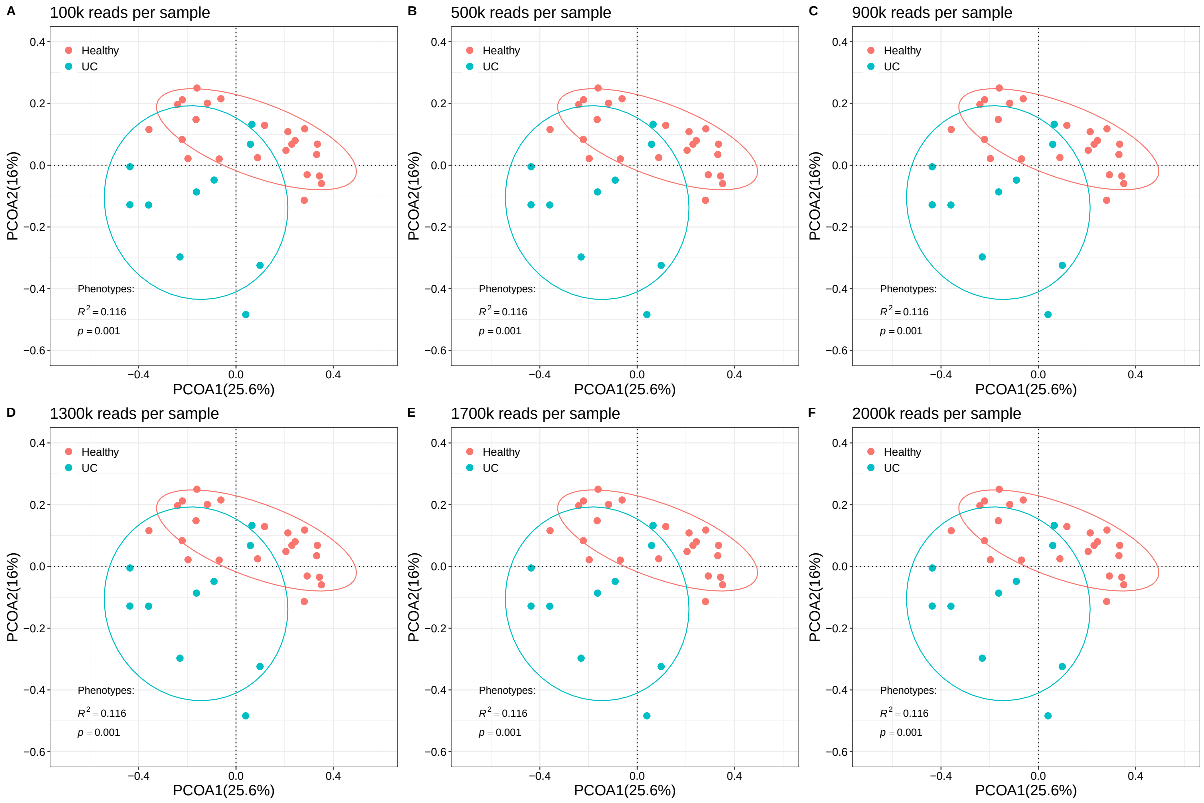

Figure S9: PCoA plots of the samples at different rarefaction levels using species abundance from the shotgun data, with colors representing disease status. The p-values for the effect of phenotypes after removing the influence of age and gender were calculated using PERMANOVA. UC cases with treatments were removed in this figure.

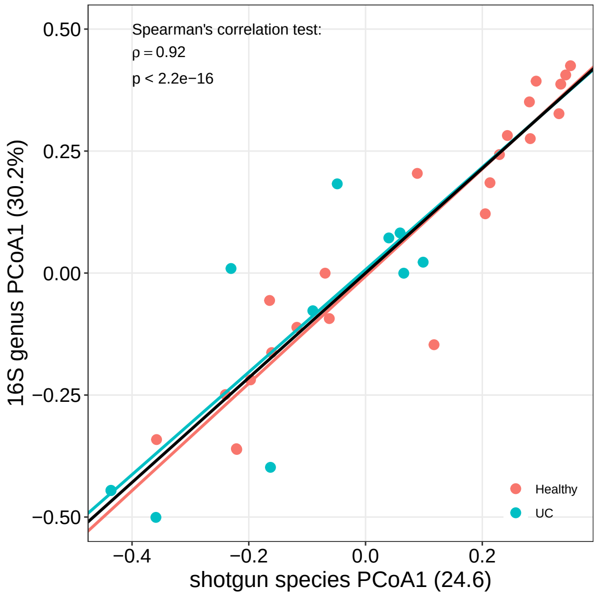

Figure S10: Correlation between the first principal coordinate (PCoA1) based on the 16S genus data and that of the shotgun species data using the Bray-Curtis distance. Green, red, and black lines represent linear trends for the UC, healthy, and overall groups, respectively. The Spearman's correlation coefficient and p-value were also included. UC cases with treatments were removed in this figure.

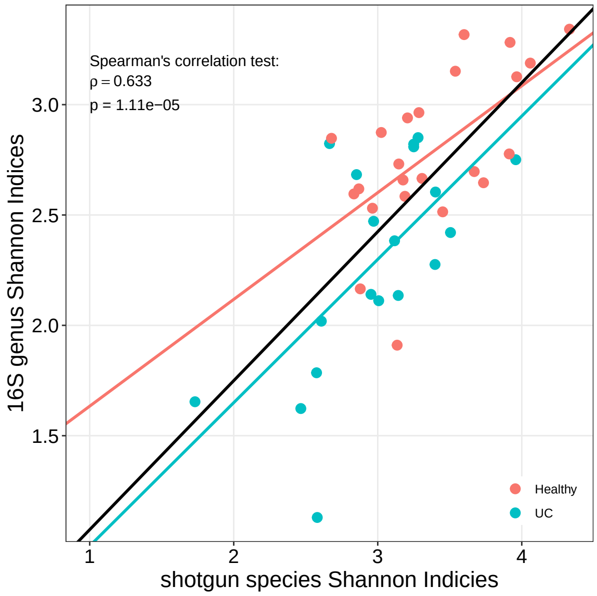

Figure S11: Correlation between the alpha diversity based on the 16S genus data and the shotgun species data using the Shannon index. Green, red, and black lines represent linear trends for the UC, healthy, and overall groups, respectively. The Spearman's correlation coefficient and p-value were also included.

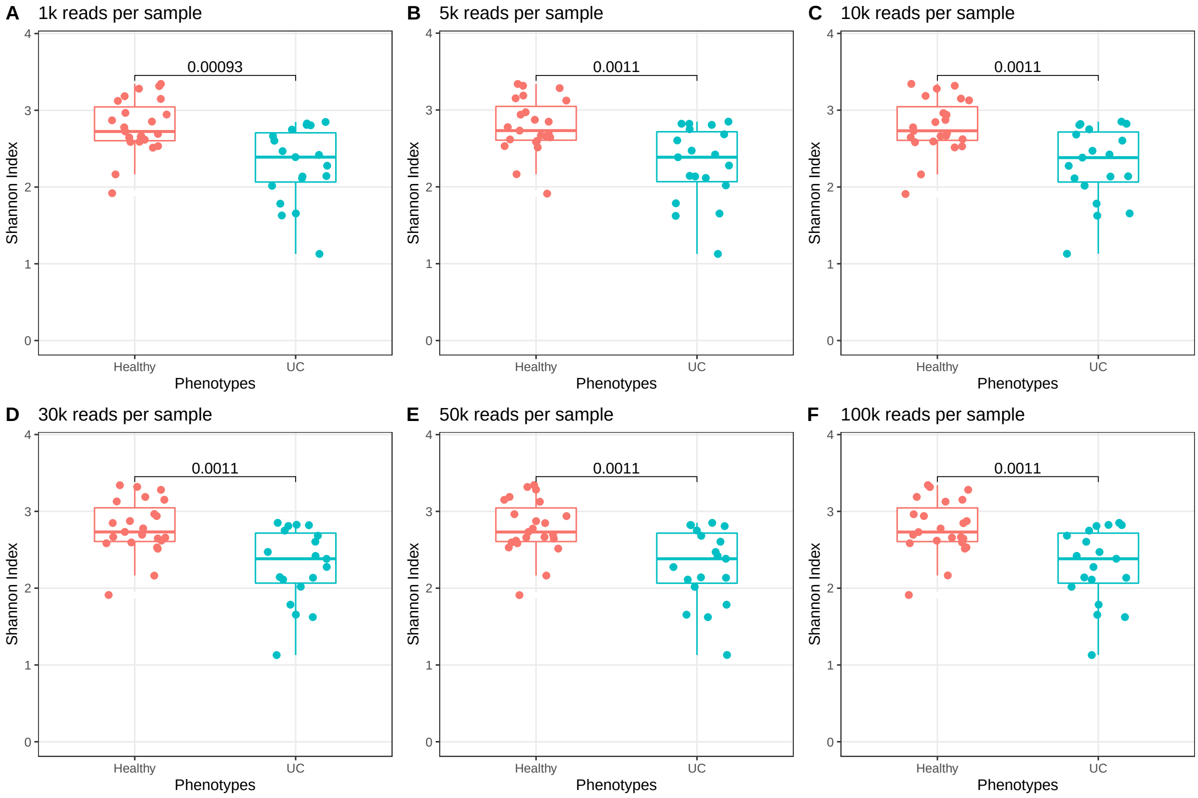

Figure S12: Box plots of Shannon indices for gut samples of pediatric UC cases and healthy controls at different rarefaction levels using genus abundance from the 16S rRNA data. The p-values were calculated using the two-sided Wilcoxon tests. Alpha diversities were not markedly influenced by the rarefaction levels.

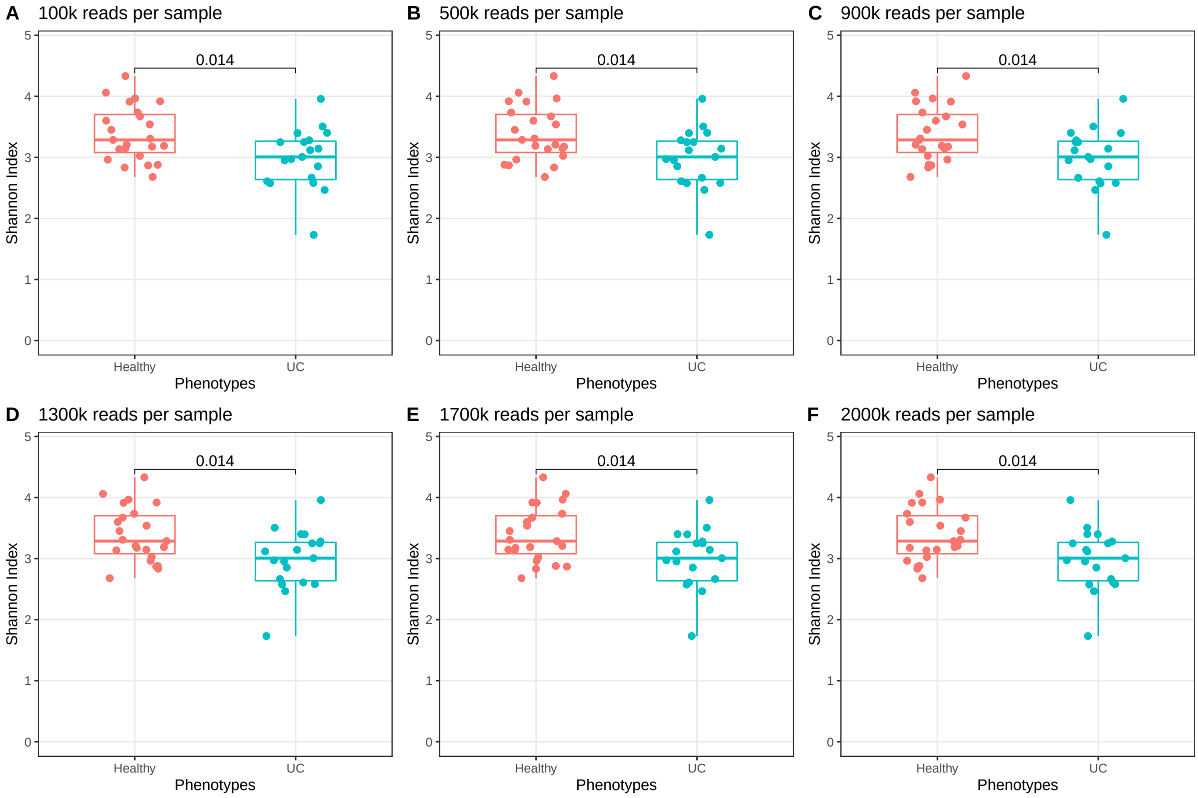

Figure S13: Box plots of Shannon indices for gut samples of pediatric UC cases and healthy controls at different rarefaction levels using species abundance from metagenomic shotgun data. The p-values were calculated using the two-sided Wilcoxon tests. Alpha diversity was not markedly influenced by the rarefaction levels.

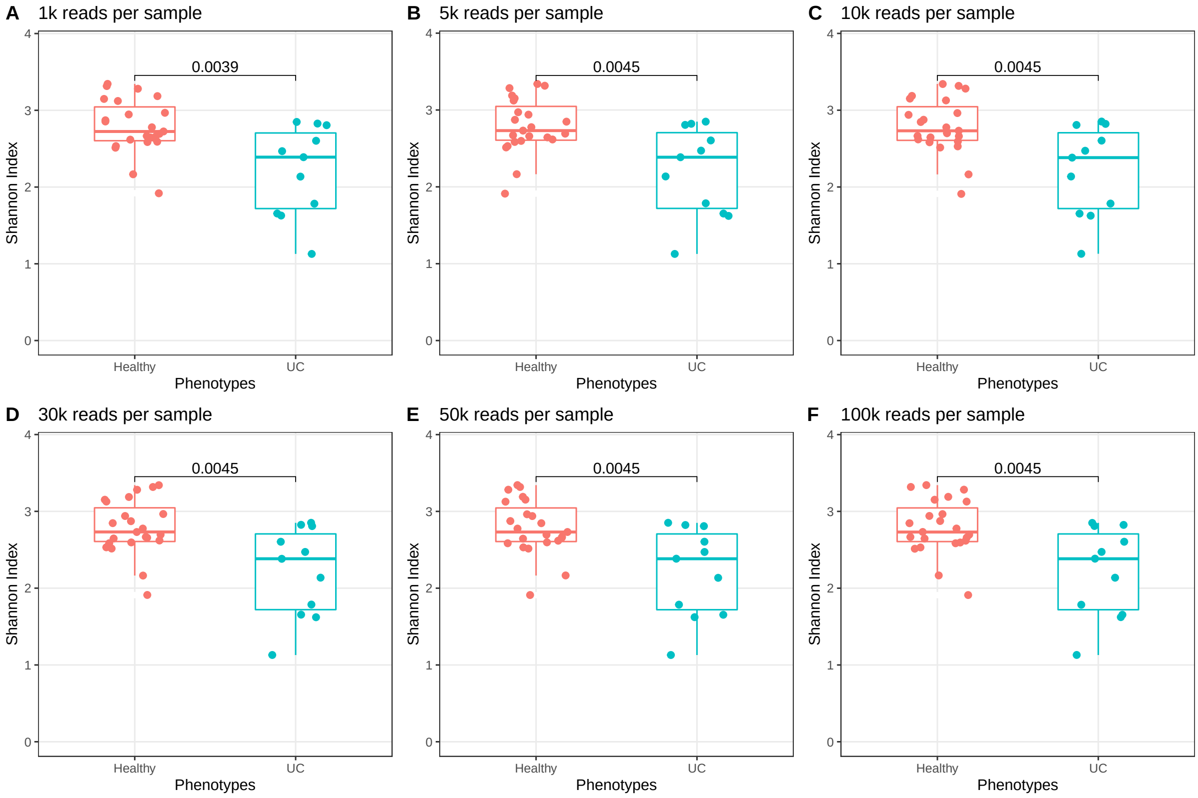

Figure S14: Box plots of Shannon indices for the gut samples of pediatric UC cases without treatments and healthy controls at different rarefaction levels using genus abundance from the 16S rRNA data. The p-values were calculated using the two-sided Wilcoxon tests. Alpha diversity was not markedly influenced by the rarefaction levels.

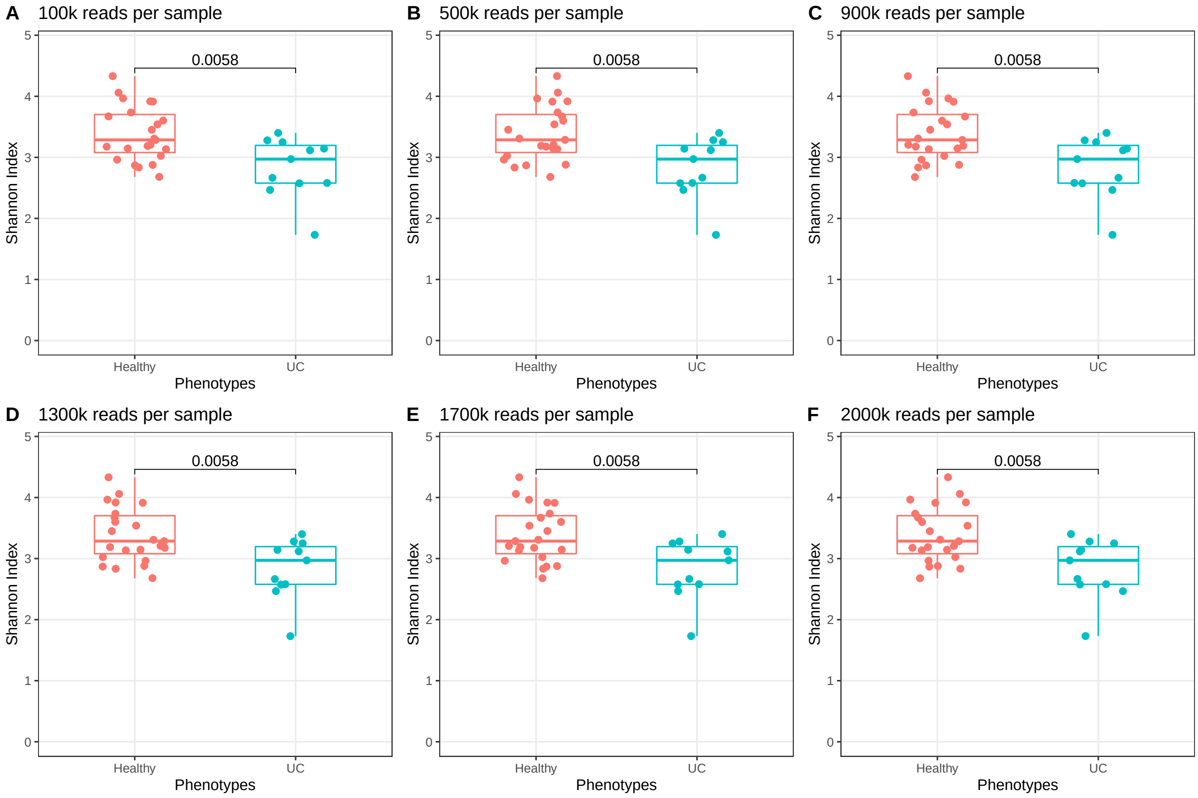

Figure S15: Box plots of Shannon indices for the gut samples of pediatric UC cases without treatments and healthy controls at different rarefaction levels using species abundance from shotgun data. The p-values were calculated using the two-sided Wilcoxon tests. Alpha diversity was not markedly influenced by the rarefaction levels.

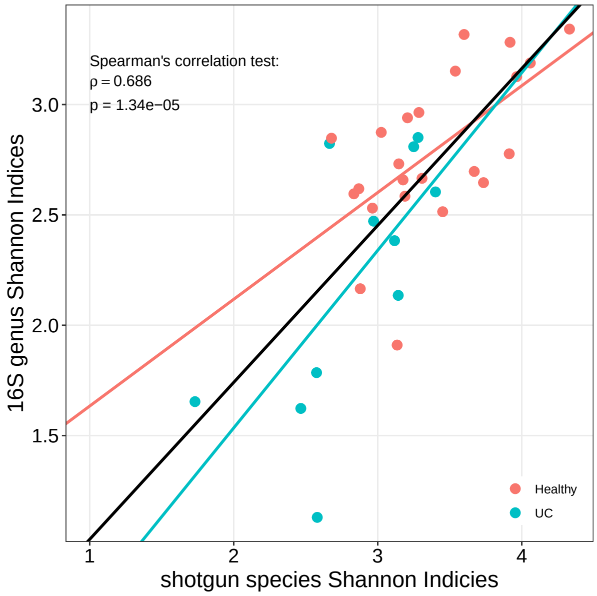

Figure S16: Correlation between the alpha diversities based on the 16S genus data and the shotgun species data using the Shannon index. Green, red, and black lines represent linear trends for the UC, healthy, and overall groups, respectively. The Spearman's correlation coefficient and p-values were also included. UC cases with treatments were removed in this figure.

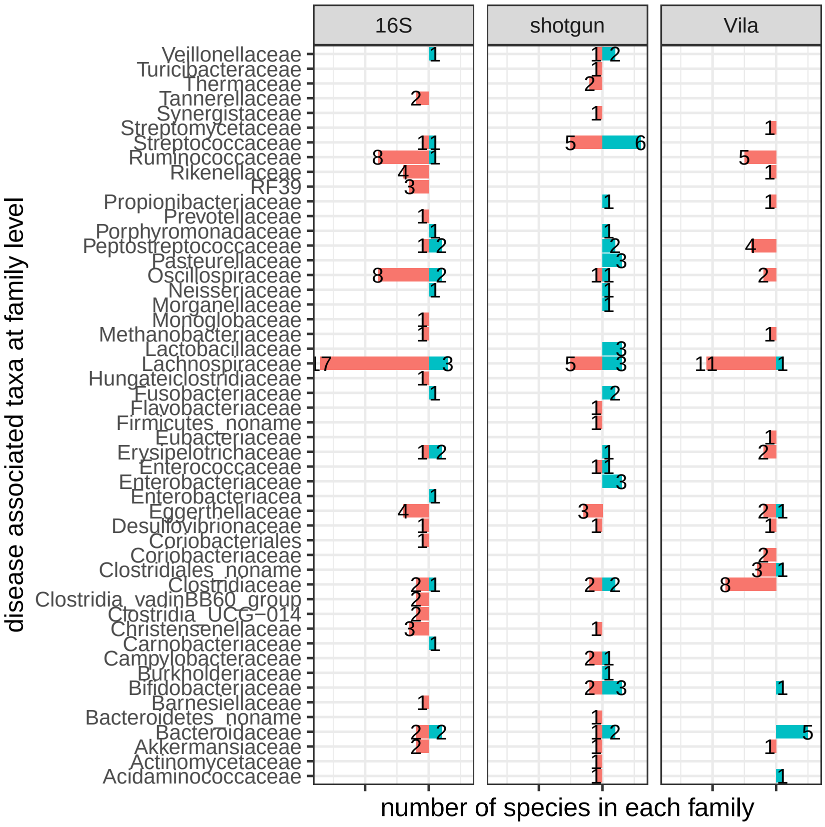

Figure S17: Comparison of UC associated species using 16S rRNA gene and shotgun short reads data with the associated species from Vila et al. after removing UC cases with treatments. For each microbial family, the numbers of species that were increased (green) or decreased (red) are shown.

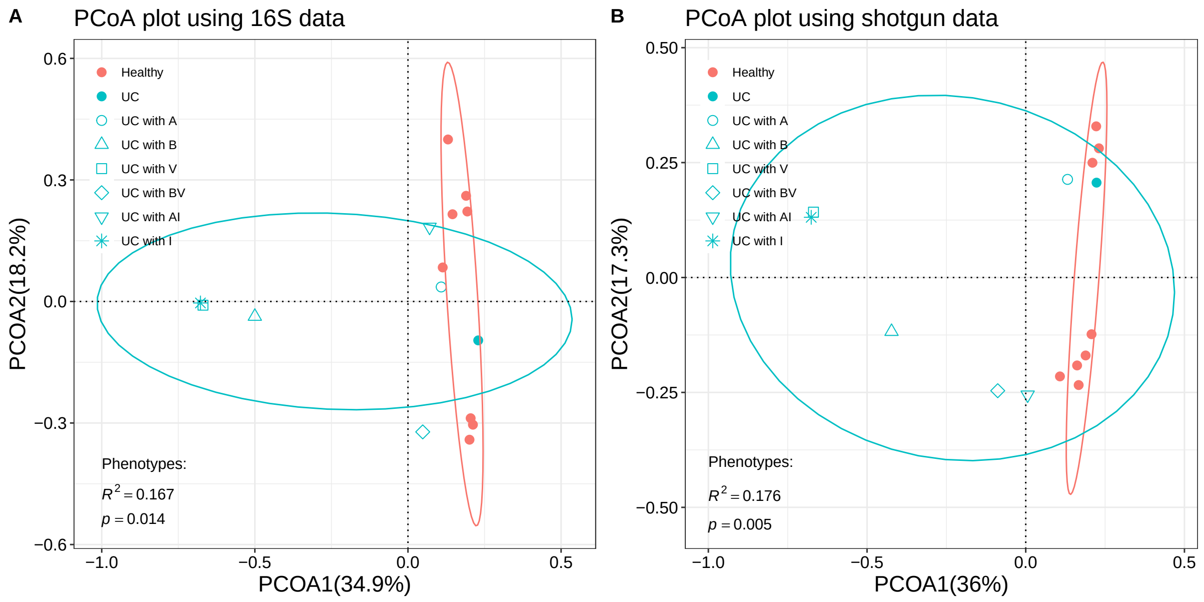

Figure S18: PCoA clustering of the samples using the independent validation data, with color for disease status and shapes for therapies of UC patients (A, patients taking 5-aminosalicylates; B, patients taking biologic therapy; I, patients taking immunomodulators; S, patients using steroid; V, patients taking vancomycin (antibiotics)). (**A**) PCoA based on Bray-Curtis distance calculated from the 16S genus level validation abundance profiles (PERMANOVA p-value 0.014). (**B**) PCoA based on Bray-Curtis distance calculated from bacterial species level abundance using the shotgun validation data (PERMANOVA p-value 0.005).

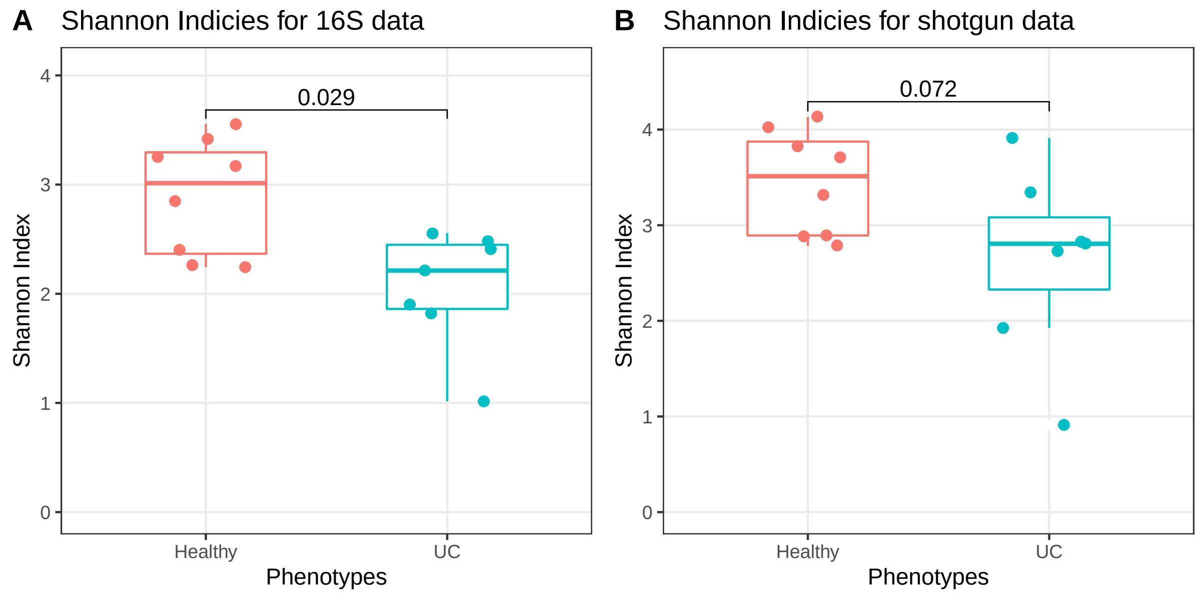

Figure S19: Box plots of Shannon indices for the validation gut samples of UC cases and healthy controls. The p-values were calculated by the Wilcoxon rank sum tests. (**A**) Shannon indices were calculated based on the genus level abundance of the 16S validation data. (**B**) Shannon indices were calculated using the bacterial species abundance based on shotgun validation data.

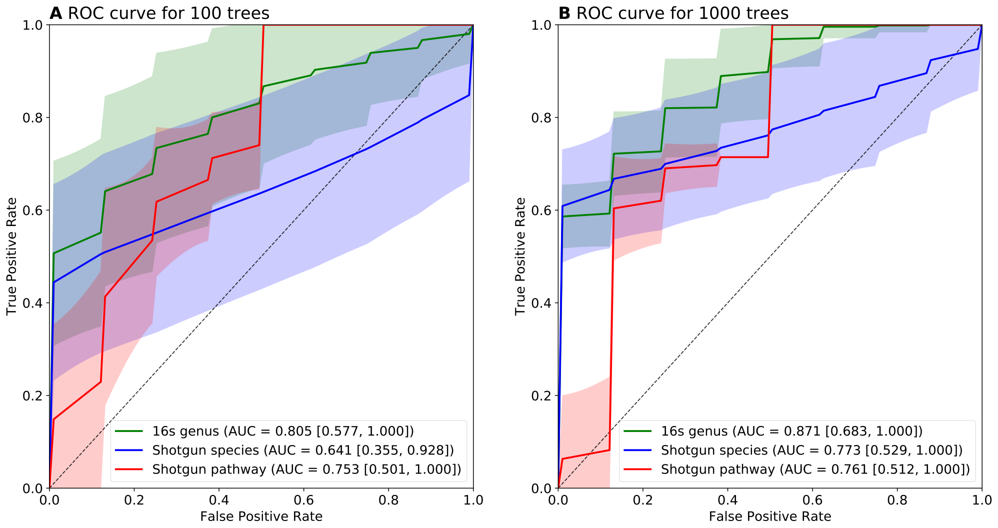

Figure S20: Validation ROC curves based on the random forests models with (**A**) 100 and (**B**) 1000 trees developed from the training data. Numbers in the square brackets represent the 95% confidence interval of the AUROC score.

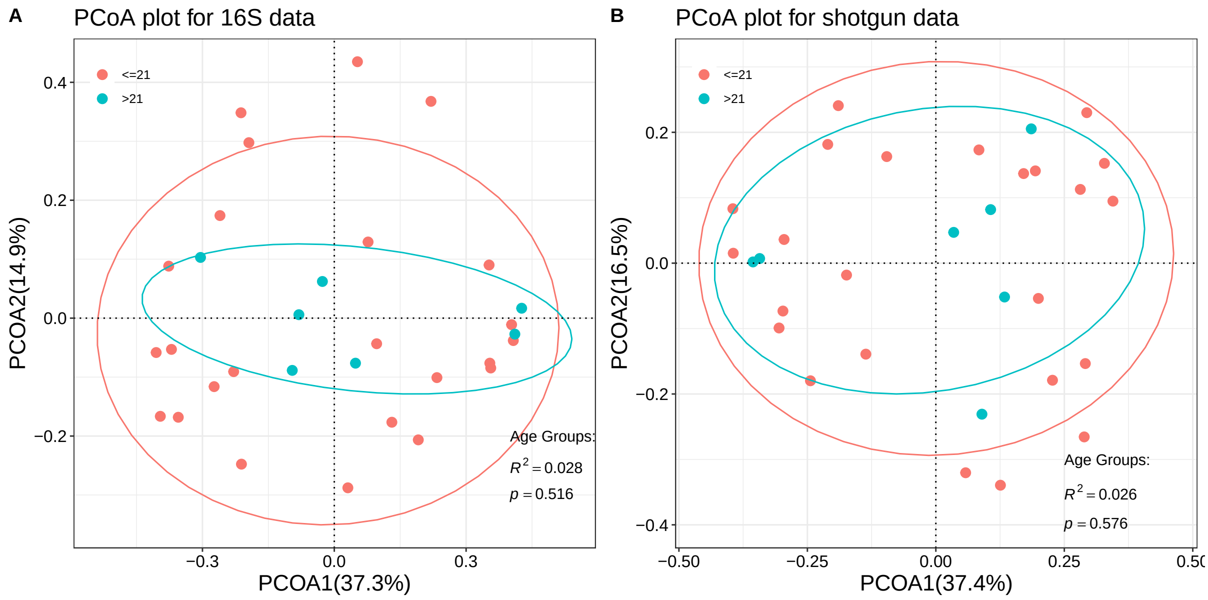

Figure S21: PCoA plots of all healthy controls in both training and validation data sets. (**A**) PCoA based on the Bray-Curtis distance calculated from the 16S genus level abundance profiles of healthy samples (PERMANOVA p-value 0.515). (**B**) PCoA based on Bray-Curtis distance calculated from bacterial species level abundance using healthy controls in the shotgun reads data (PERMANOVA p-value 0.001).

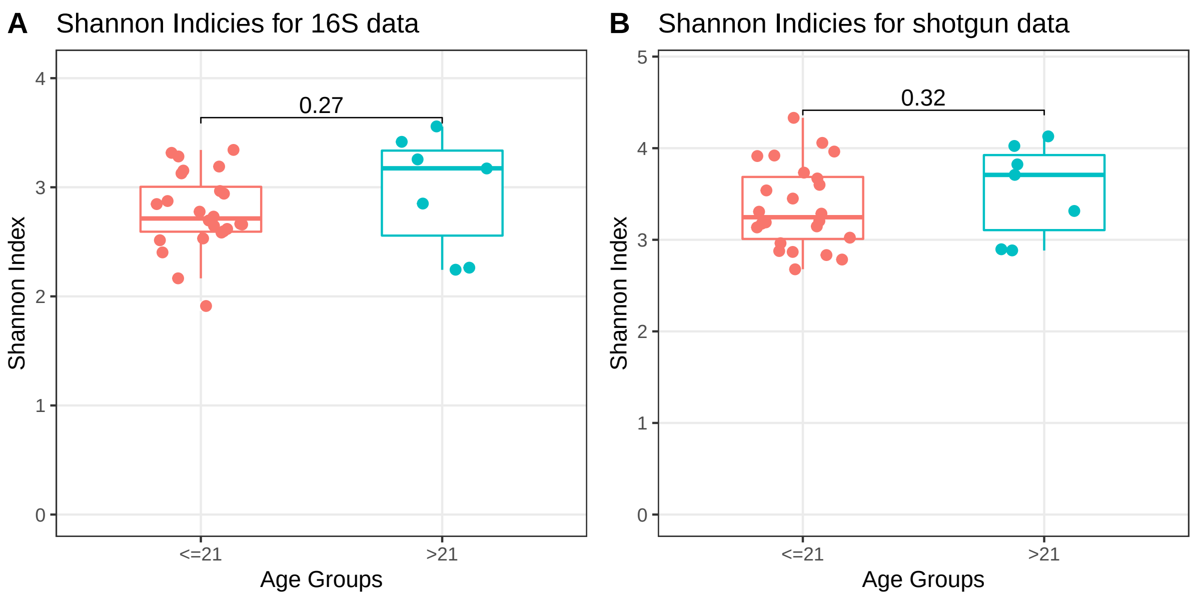

Figure S22: Box plots of Shannon indices for gut samples of healthy controls in both training and validation data sets stratified by their age groups. The p-values were calculated by the Wilcoxon rank sum test. (**A**) Shannon indices were calculated based on the genus level abundance of 16s healthy controls. (**B**) Shannon indices were calculated using the bacterial species abundance based on healthy controls in the shotgun reads data.
