# Supplementary material for: 16S rRNA and metagenomic shotgun sequencing data revealed consistent patterns of gut microbiome signature in pediatric ulcerative colitis
Source: Sci Rep. 2022 Apr 19;12:6421. doi: 10.1038/s41598-022-07995-7 (PMC9018687; doi:10.1038/s41598-022-07995-7)
Supplement: Supplementary file 3 — Supplementary Information 3. [file 41598_2022_7995_MOESM3_ESM.zip › supplementary_tex/amplicon_rarefied_shannon_boxplots.pdf]

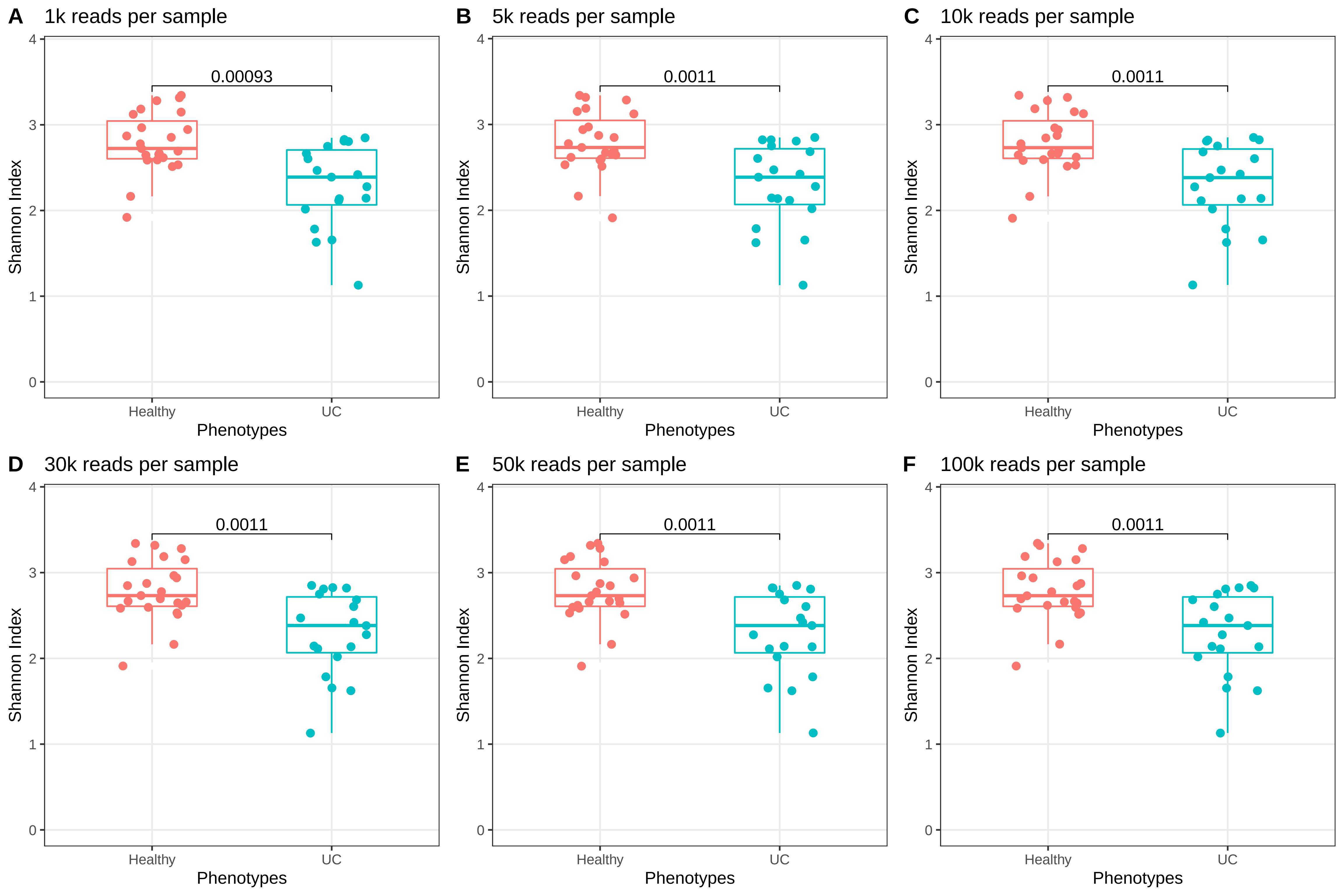

A
1k reads per sample
0.00093
B
5k reads per sample
0.0011
C
10k reads per sample
0.0011
D
30k reads per sample
0.0011
E
50k reads per sample
0.0011
F
100k reads per sample
0.0011
Shannon Index
Phenotypes
Healthy
UC
0
1
2
3
4
