# Supplementary material for: 16S rRNA and metagenomic shotgun sequencing data revealed consistent patterns of gut microbiome signature in pediatric ulcerative colitis
Source: Sci Rep. 2022 Apr 19;12:6421. doi: 10.1038/s41598-022-07995-7 (PMC9018687; doi:10.1038/s41598-022-07995-7)
Supplement: Supplementary file 3 — Supplementary Information 3. [file 41598_2022_7995_MOESM3_ESM.zip › supplementary_tex/amplicon_rarefied_pcoa.pdf]

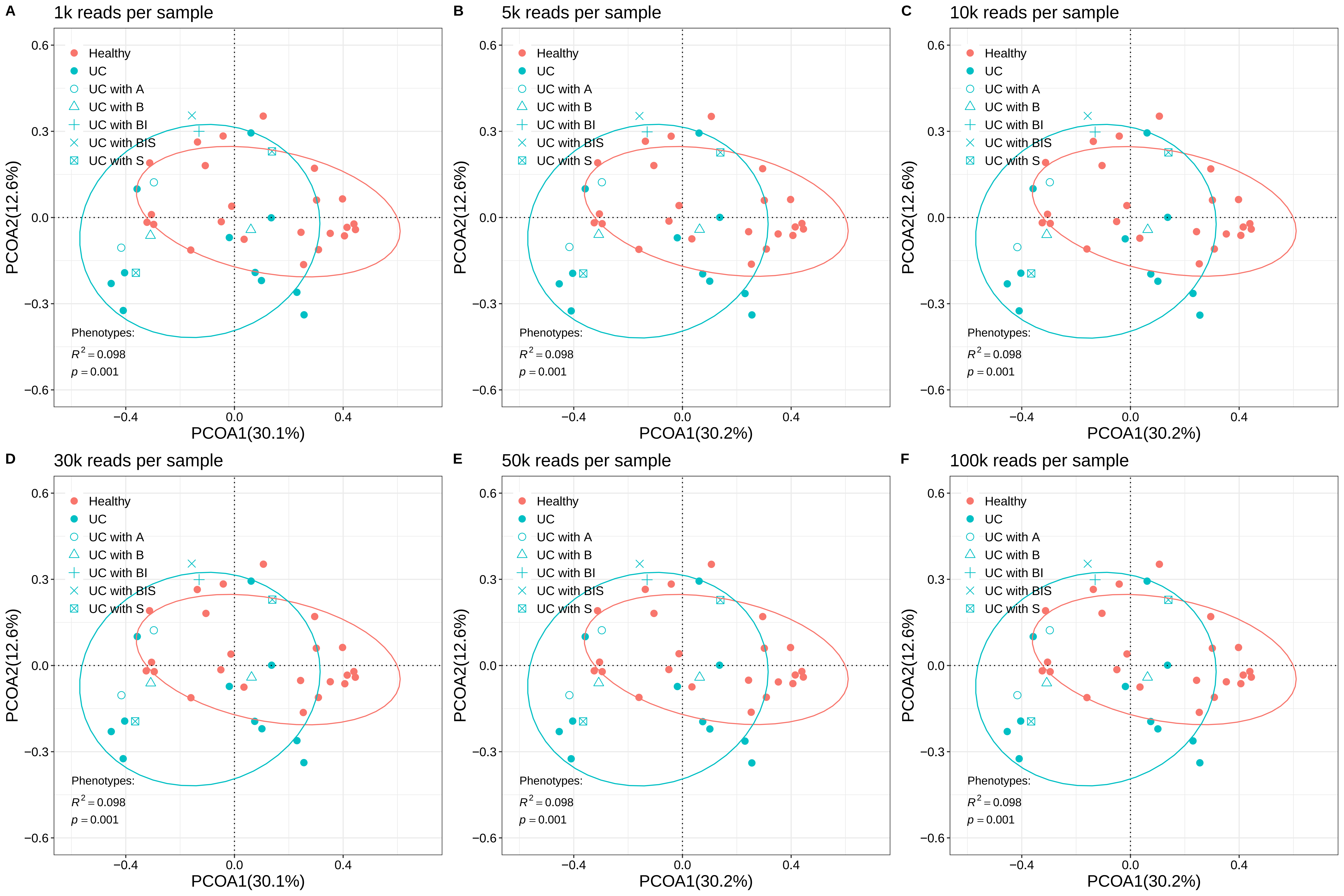
A
1k reads per sample
B
5k reads per sample
C
10k reads per sample
D
30k reads per sample
E
50k reads per sample
F
100k reads per sample
Healthy
UC
UC with A
UC with B
UC with BI
UC with BIS
UC with S
Phenotypes:
$R^2 = 0.098$
$p = 0.001$
PCOA1(30.1%)
PCOA1(30.2%)
PCOA2(12.6%)
0.6
0.3
0.0
−0.3
−0.6
−0.4
0.0
0.4
