# Supplementary material for: 16S rRNA and metagenomic shotgun sequencing data revealed consistent patterns of gut microbiome signature in pediatric ulcerative colitis
Source: Sci Rep. 2022 Apr 19;12:6421. doi: 10.1038/s41598-022-07995-7 (PMC9018687; doi:10.1038/s41598-022-07995-7)
Supplement: Supplementary file 3 — Supplementary Information 3. [file 41598_2022_7995_MOESM3_ESM.zip › supplementary_tex/amplicon_rarefied_pcoa_remove_uc_therapy.pdf]

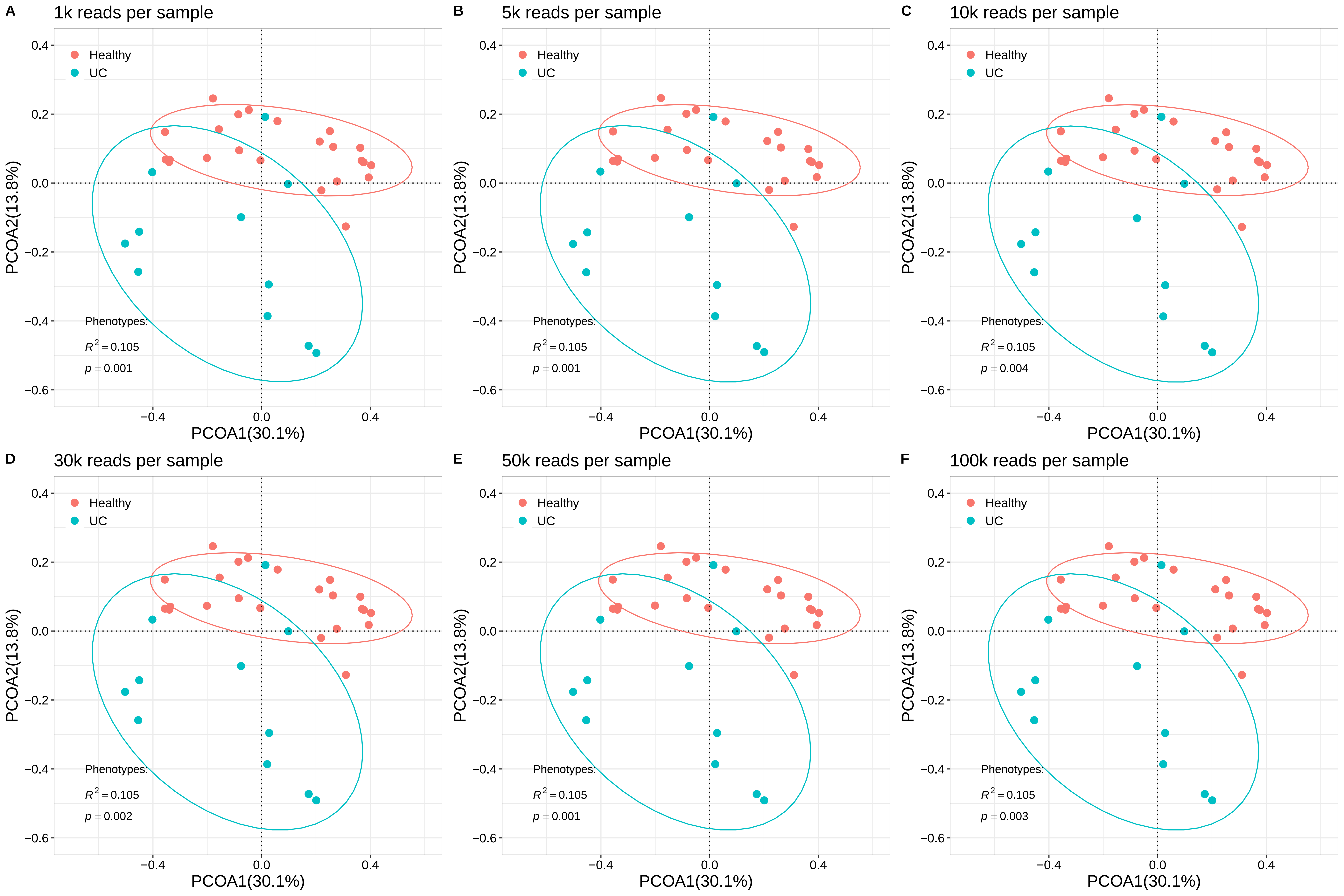
A
1k reads per sample
Healthy
UC
Phenotypes:
$R^2 = 0.105$
$p = 0.001$
PCOA1(30.1%)
PCOA2(13.8%)
B
5k reads per sample
Healthy
UC
Phenotypes:
$R^2 = 0.105$
$p = 0.001$
PCOA1(30.1%)
PCOA2(13.8%)
C
10k reads per sample
Healthy
UC
Phenotypes:
$R^2 = 0.105$
$p = 0.004$
PCOA1(30.1%)
PCOA2(13.8%)
D
30k reads per sample
Healthy
UC
Phenotypes:
$R^2 = 0.105$
$p = 0.002$
PCOA1(30.1%)
PCOA2(13.8%)
E
50k reads per sample
Healthy
UC
Phenotypes:
$R^2 = 0.105$
$p = 0.001$
PCOA1(30.1%)
PCOA2(13.8%)
F
100k reads per sample
Healthy
UC
Phenotypes:
$R^2 = 0.105$
$p = 0.003$
PCOA1(30.1%)
PCOA2(13.8%)
