# Supplementary figures and images for: 16S rRNA and metagenomic shotgun sequencing data revealed consistent patterns of gut microbiome signature in pediatric ulcerative colitis
Source: Sci Rep. 2022 Apr 19;12:6421. doi: 10.1038/s41598-022-07995-7 (PMC9018687; doi:10.1038/s41598-022-07995-7)

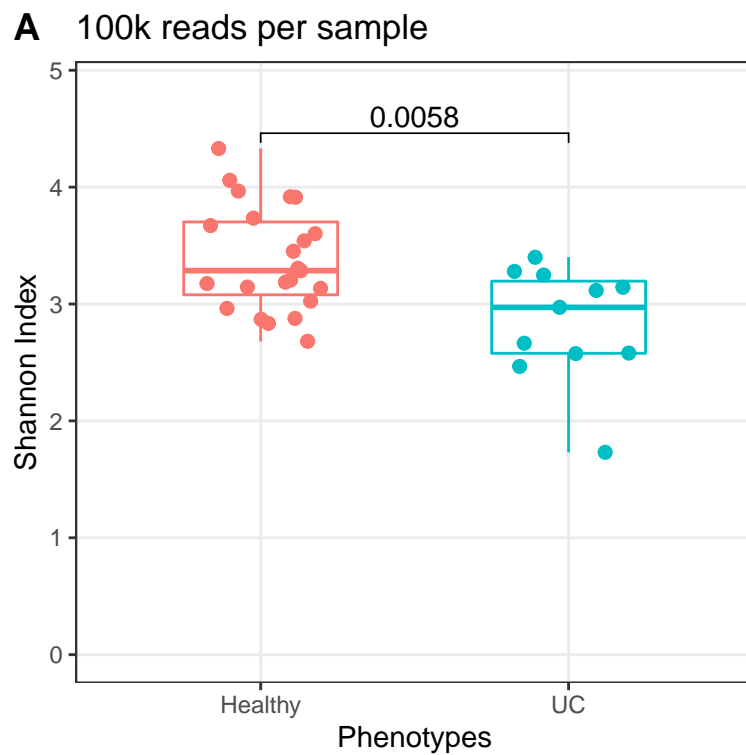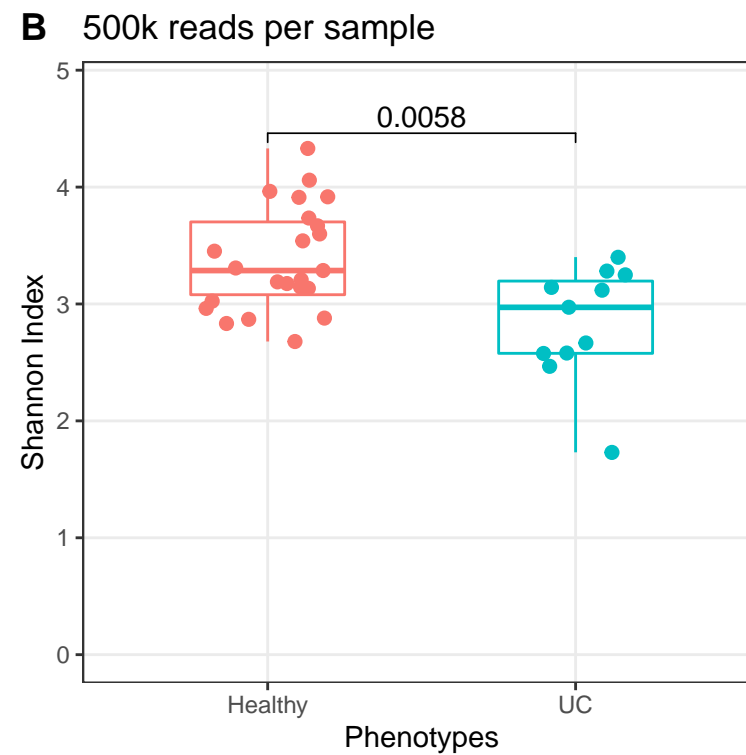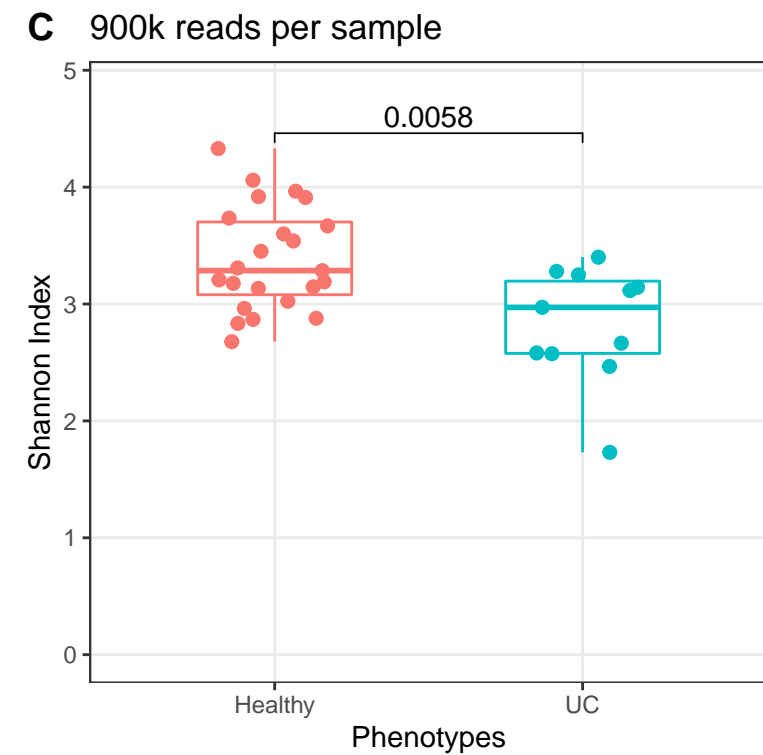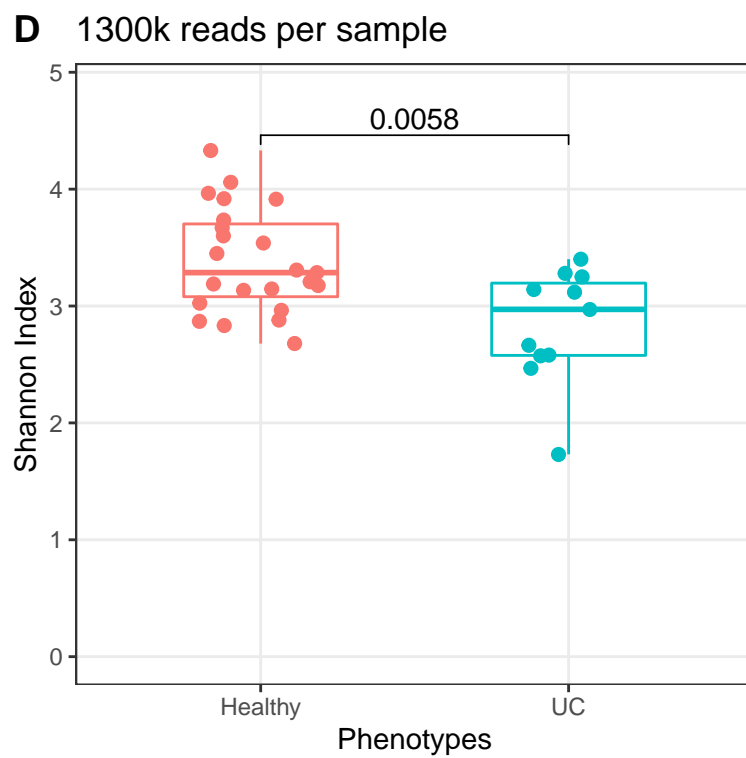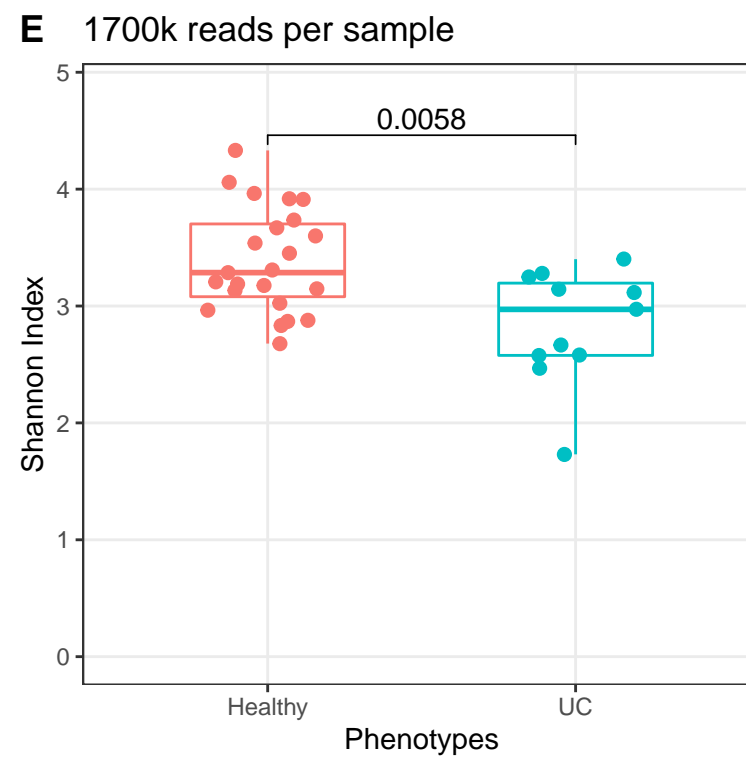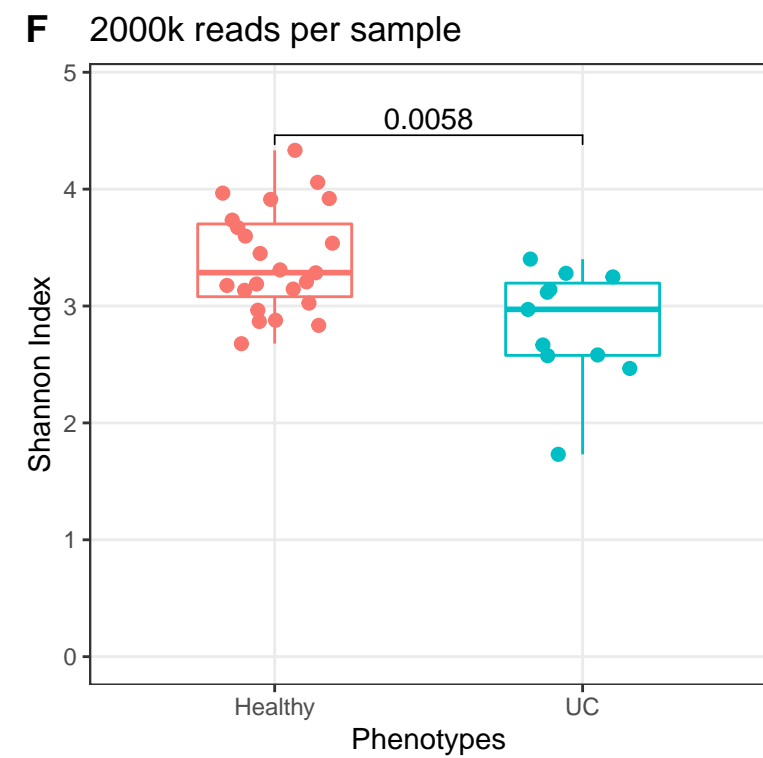

Supplement: Supplementary file 3 — Supplementary Information 3. [file 41598_2022_7995_MOESM3_ESM.zip › supplementary_tex/shotgun_rarefied_shannon_boxplots_remove_uc_therapy.pdf]

disease associated taxa at family level

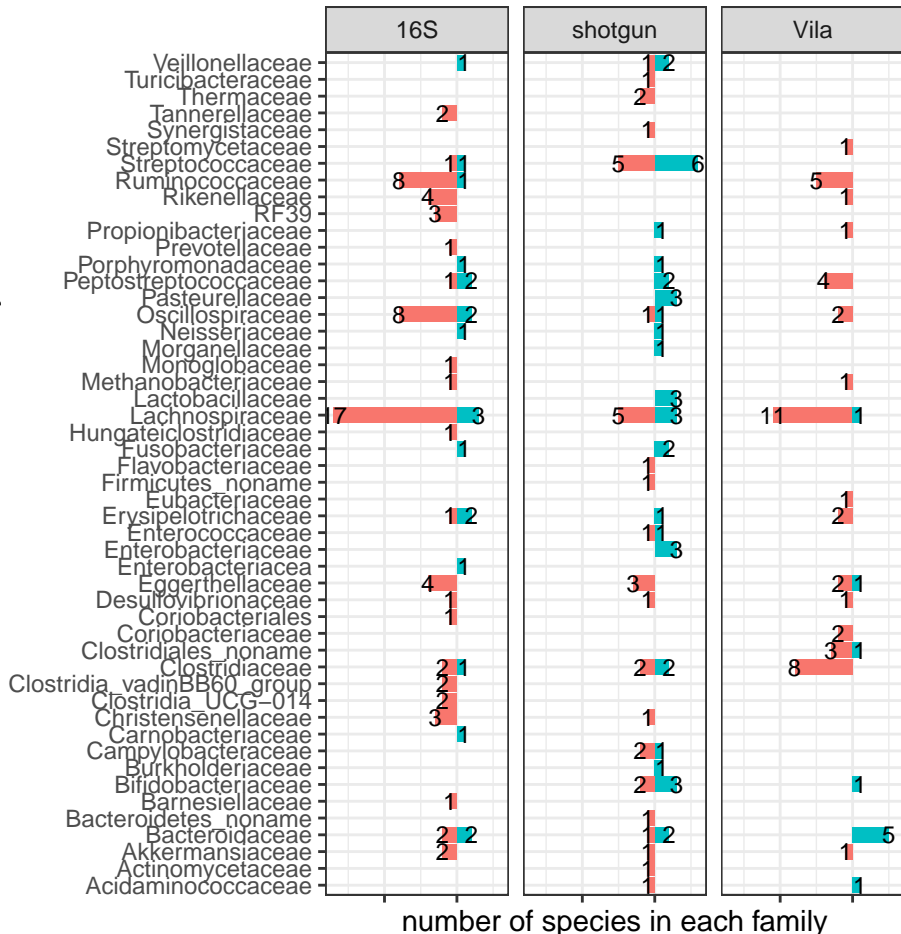

Supplement: Supplementary file 3 — Supplementary Information 3. [file 41598_2022_7995_MOESM3_ESM.zip › supplementary_tex/comparison_remove_uc_therapy.pdf]

**A** Rarefaction curve for 16S data

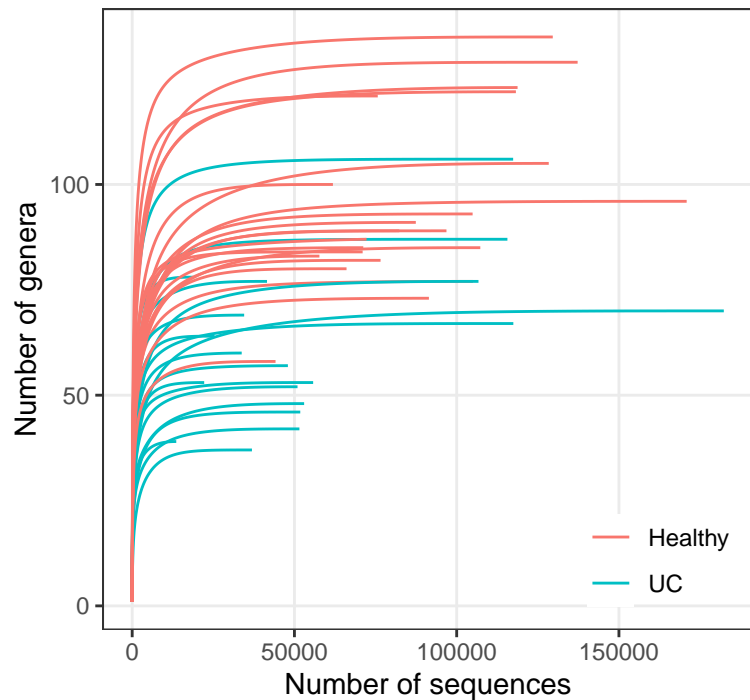

**B** Rarefaction curve for shotgun data

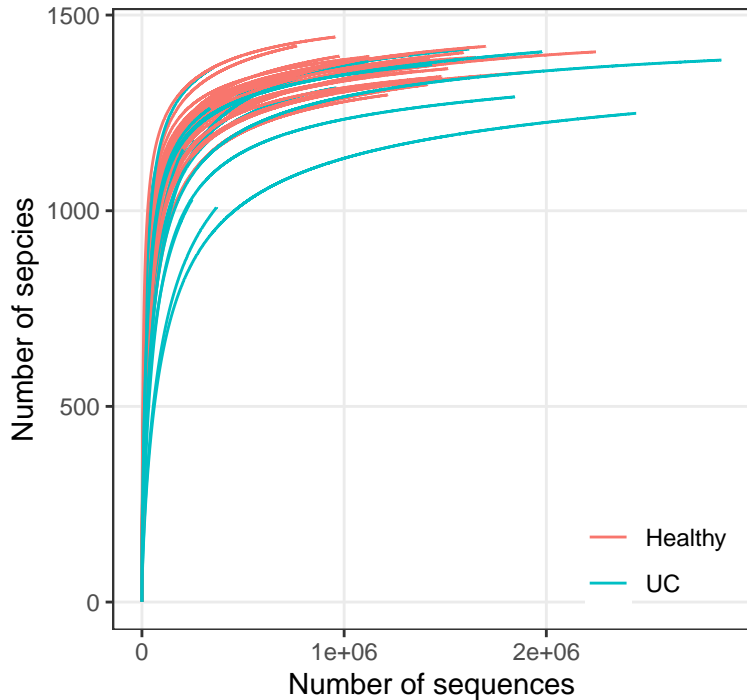

Supplement: Supplementary file 3 — Supplementary Information 3. [file 41598_2022_7995_MOESM3_ESM.zip › supplementary_tex/rarefaction_curve.pdf]

**A** PCoA plot using 16S data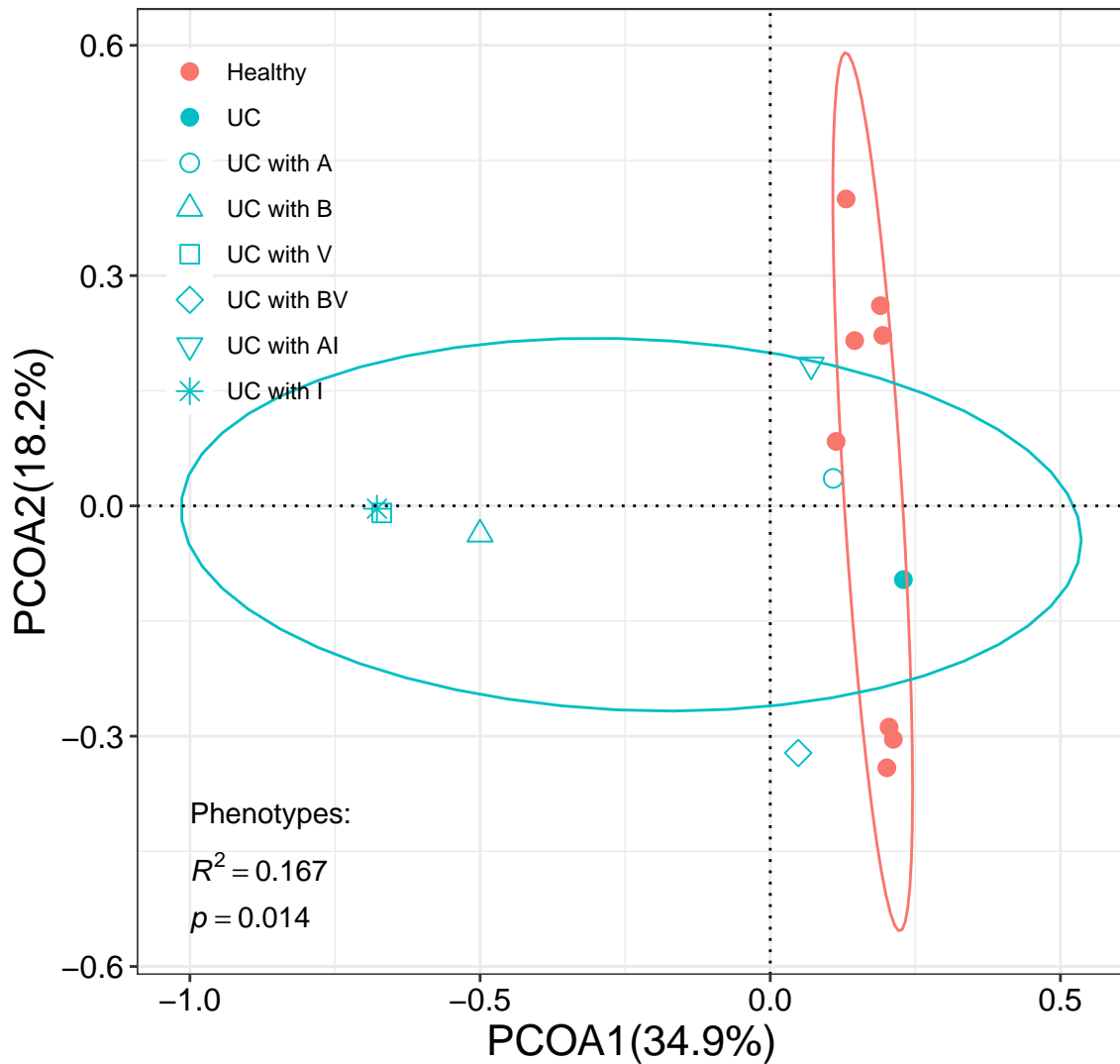**B** PCoA plot using shotgun data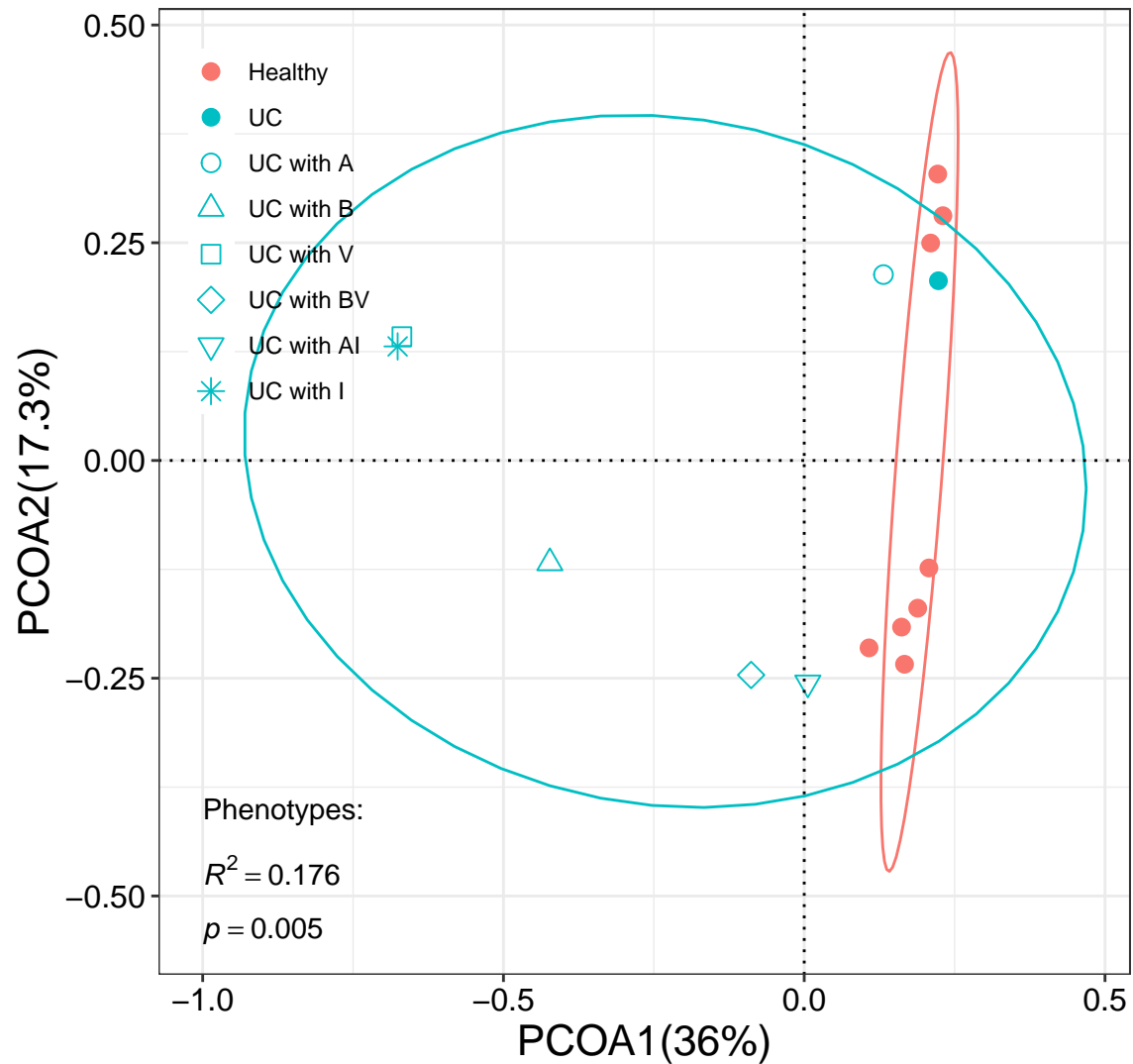

Supplement: Supplementary file 3 — Supplementary Information 3. [file 41598_2022_7995_MOESM3_ESM.zip › supplementary_tex/validation_pcoa.pdf]

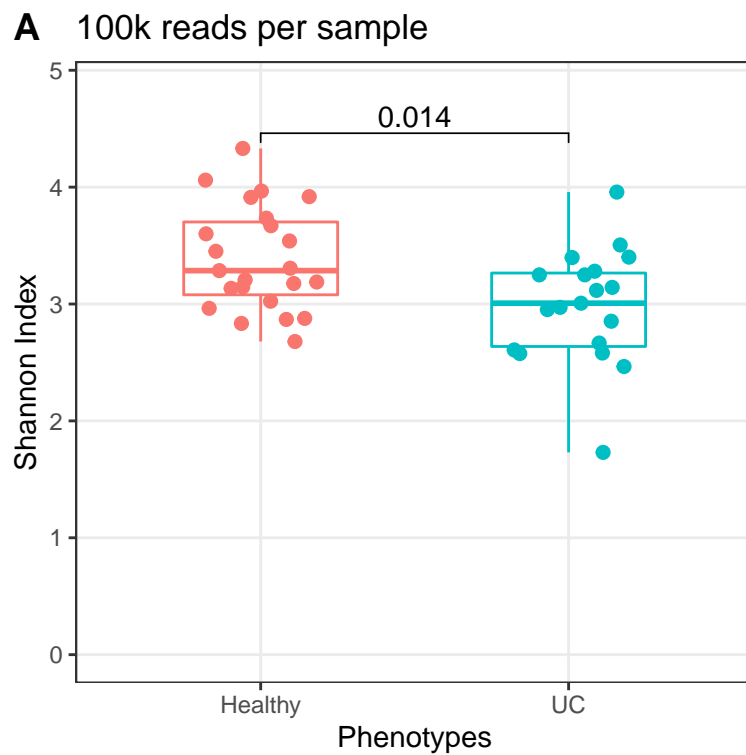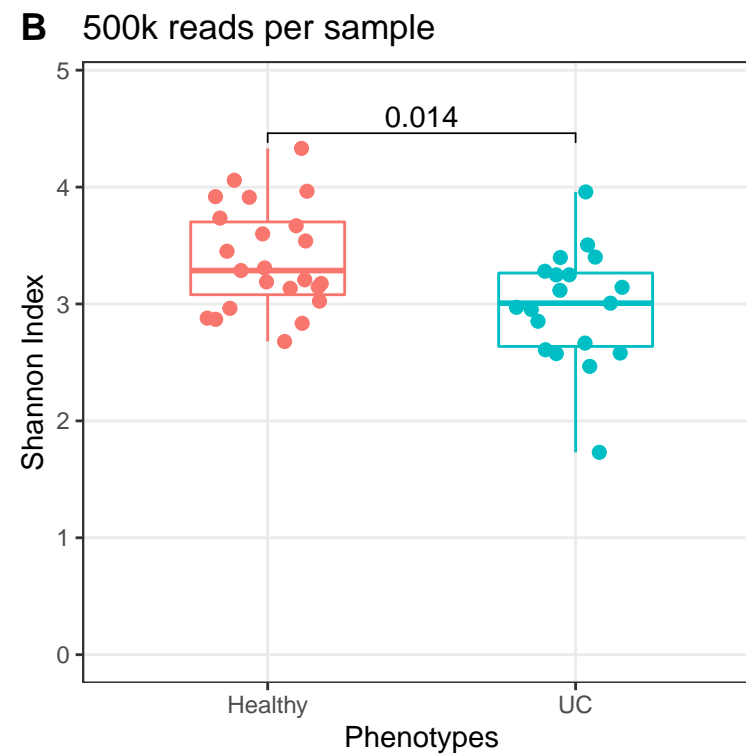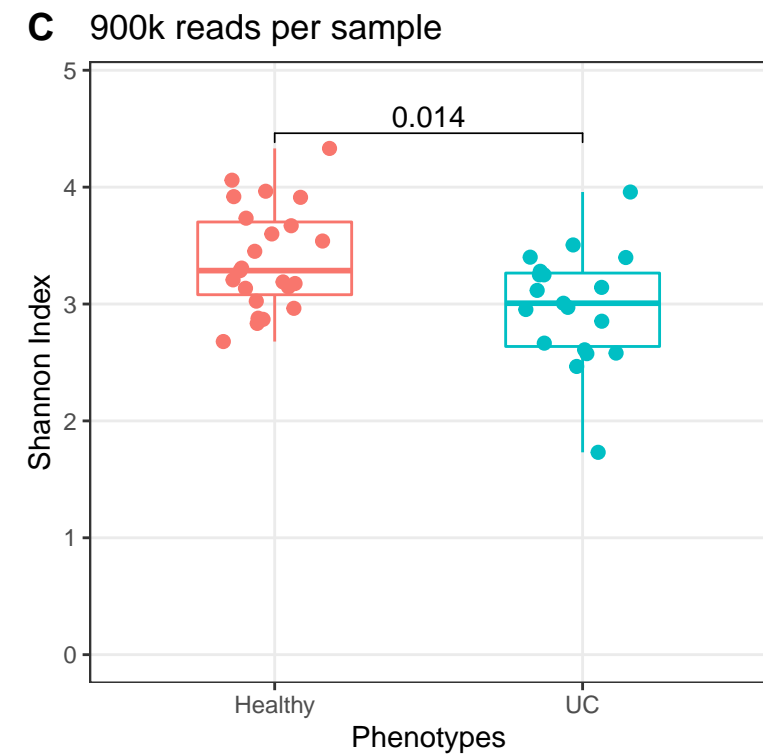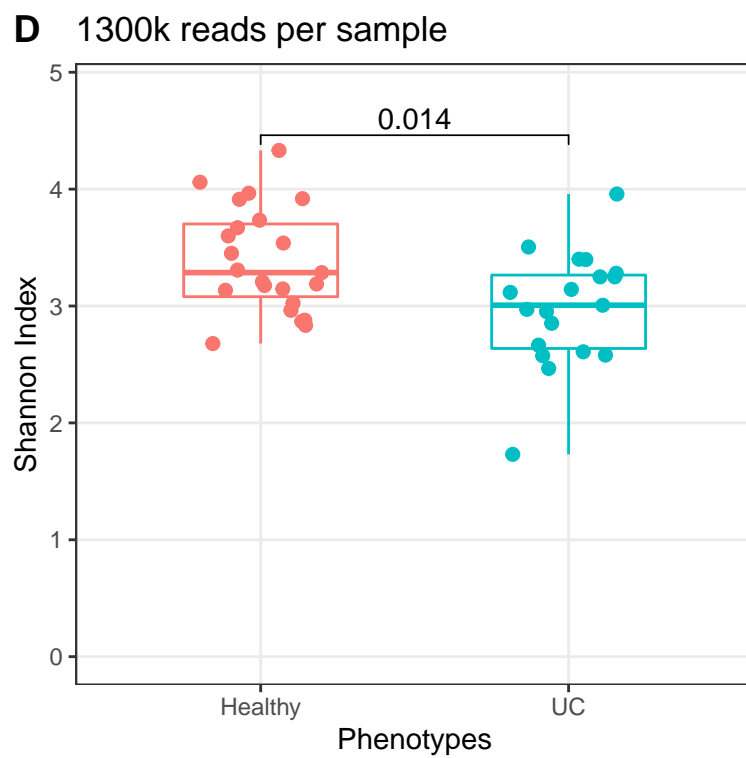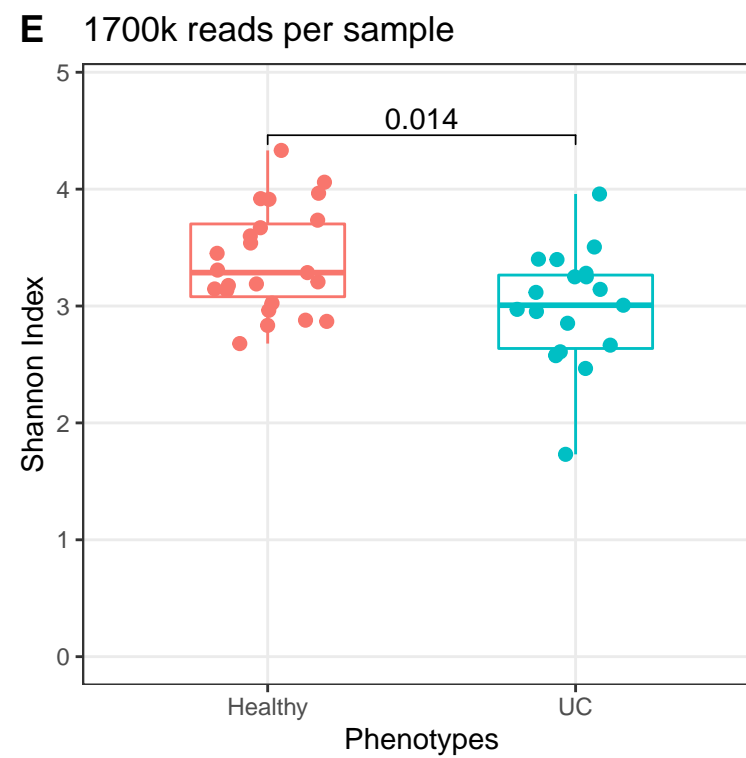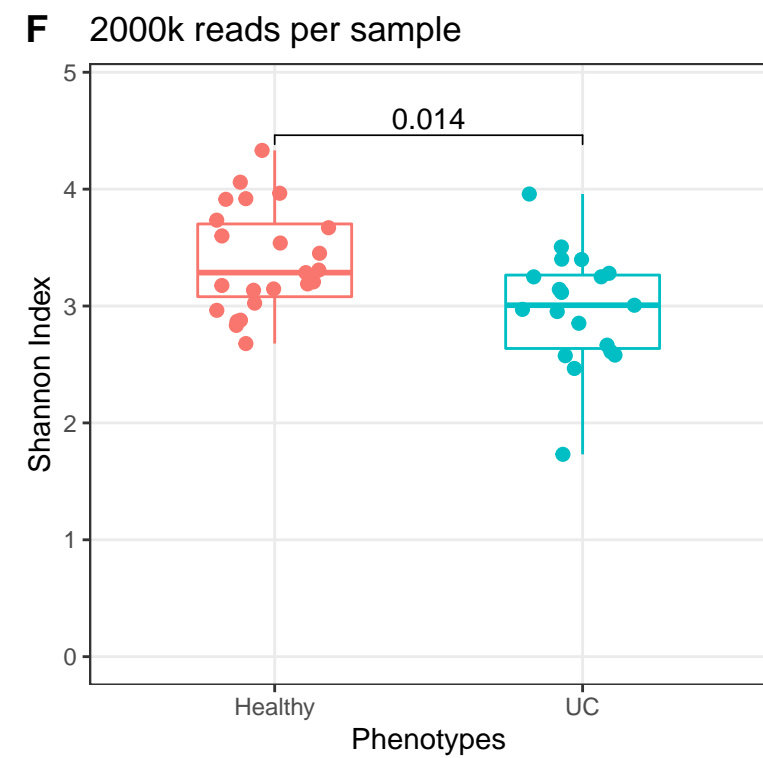

Supplement: Supplementary file 3 — Supplementary Information 3. [file 41598_2022_7995_MOESM3_ESM.zip › supplementary_tex/shotgun_rarefaction.pdf]

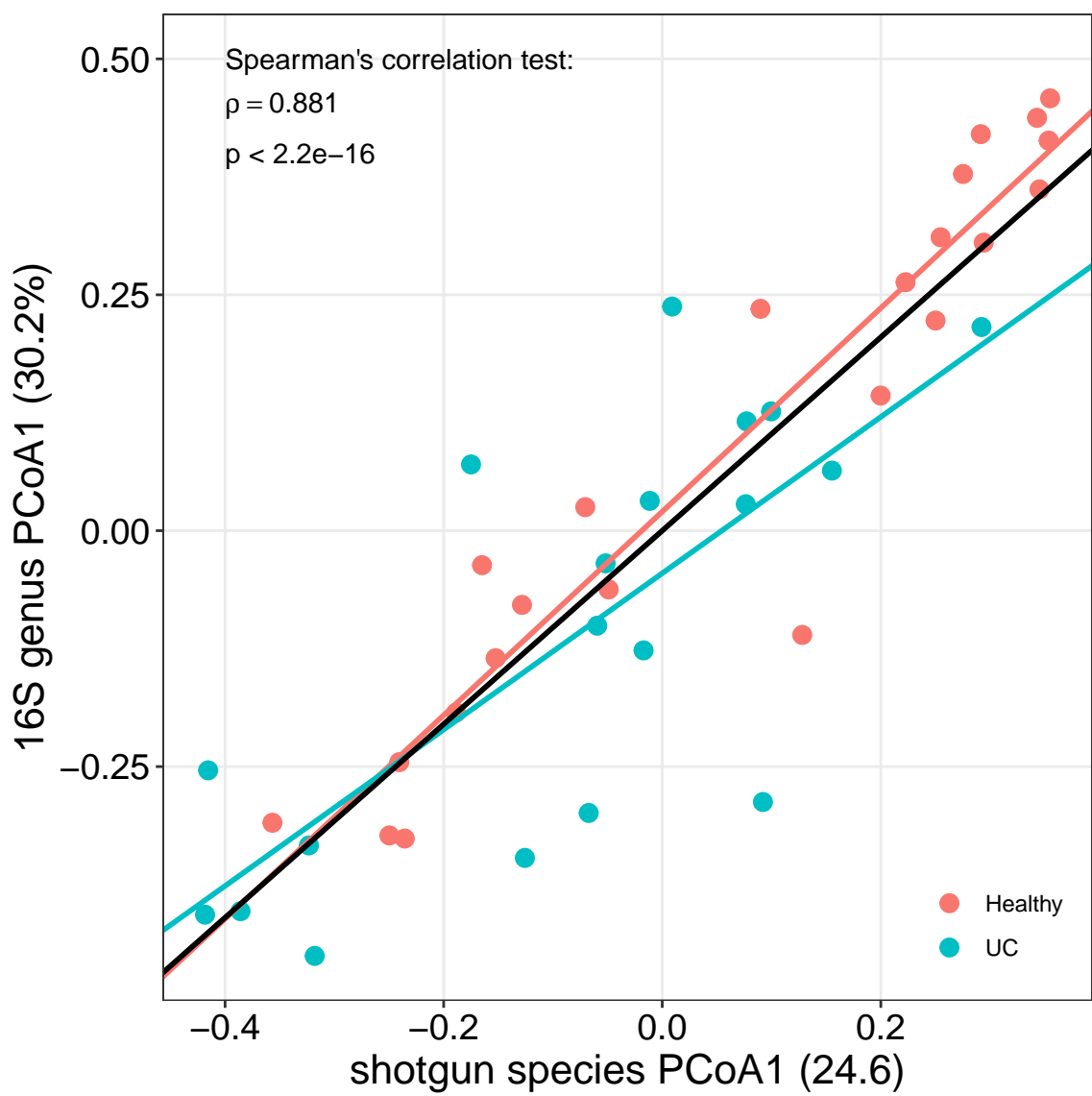

Supplement: Supplementary file 3 — Supplementary Information 3. [file 41598_2022_7995_MOESM3_ESM.zip › supplementary_tex/corr_pcoa1.pdf]

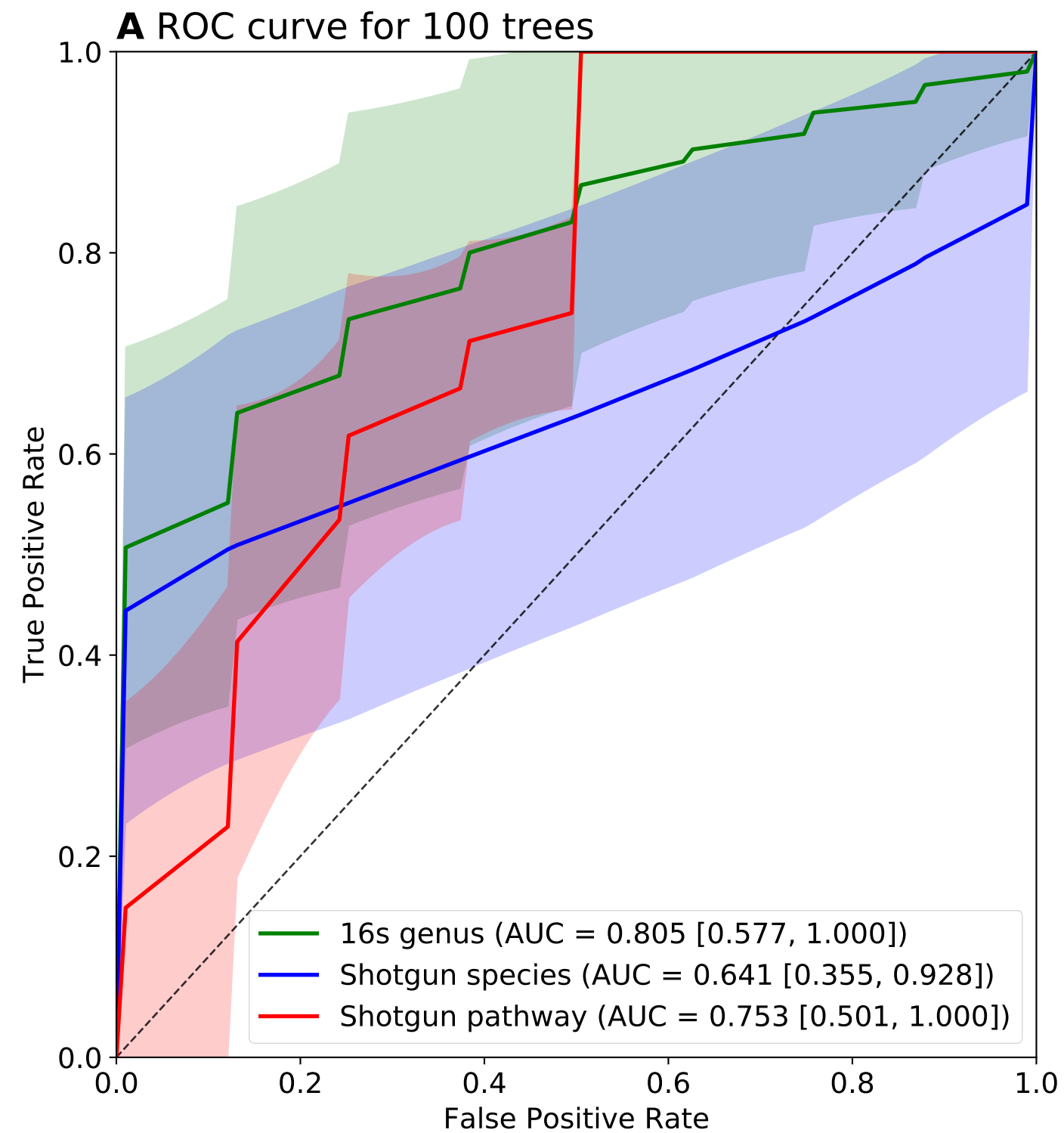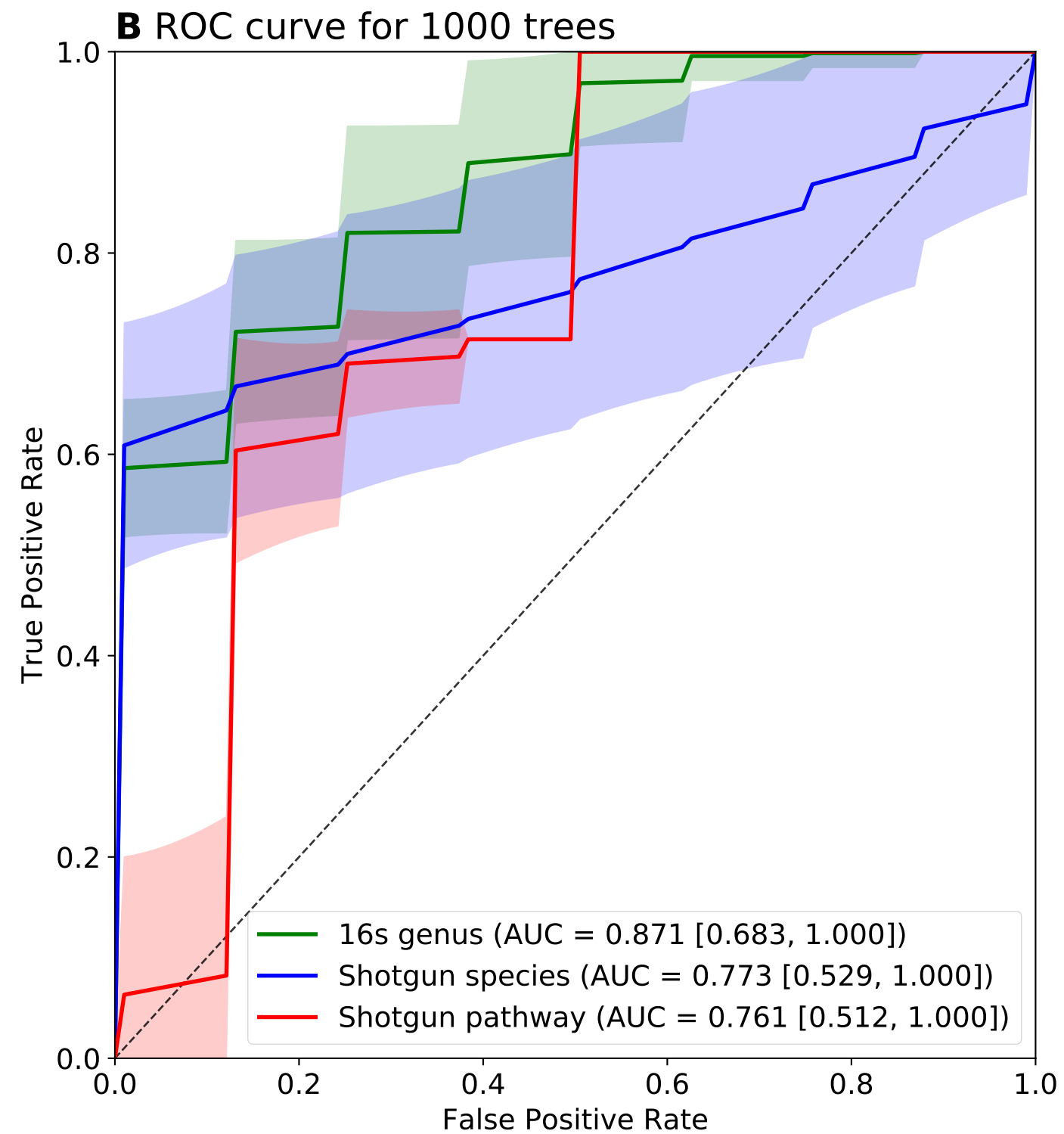

Supplement: Supplementary file 3 — Supplementary Information 3. [file 41598_2022_7995_MOESM3_ESM.zip › supplementary_tex/validation_roc_curve_100_1000.pdf]

**A** Shannon Indices for 16S data

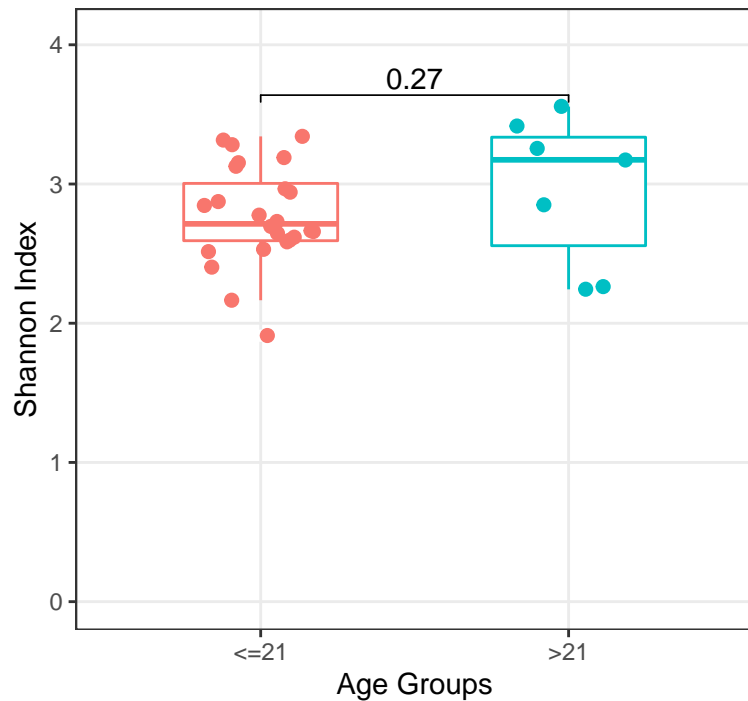

**B** Shannon Indices for shotgun data

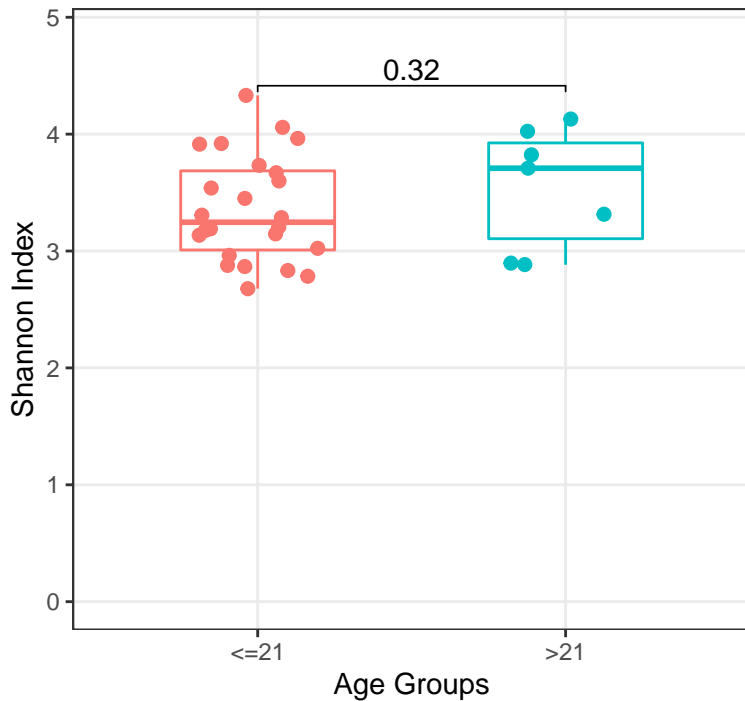

Supplement: Supplementary file 3 — Supplementary Information 3. [file 41598_2022_7995_MOESM3_ESM.zip › supplementary_tex/validation_healthy_shannon.pdf]

**A** PCoA plot using 16S data

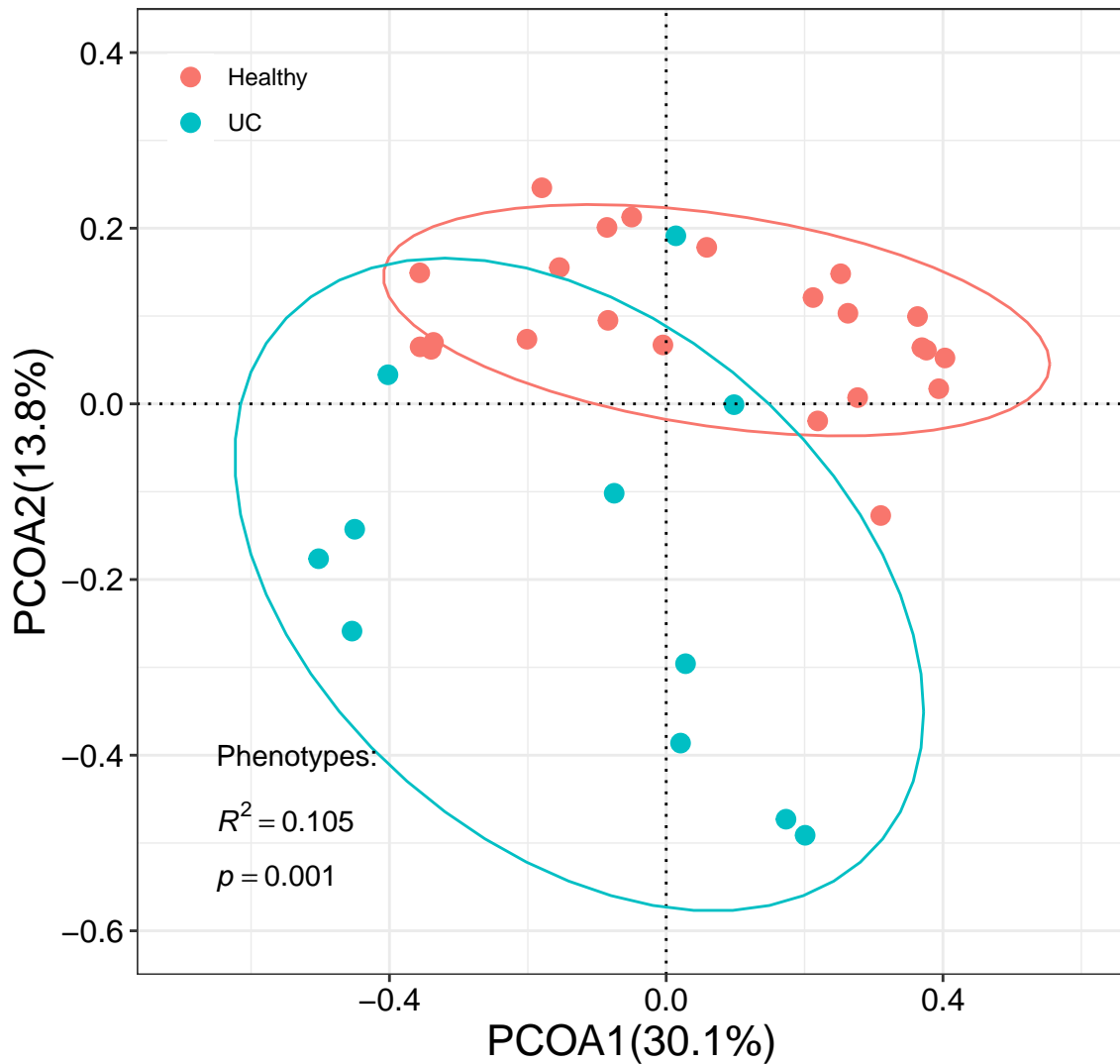

**B** PCoA plot using shotgun data

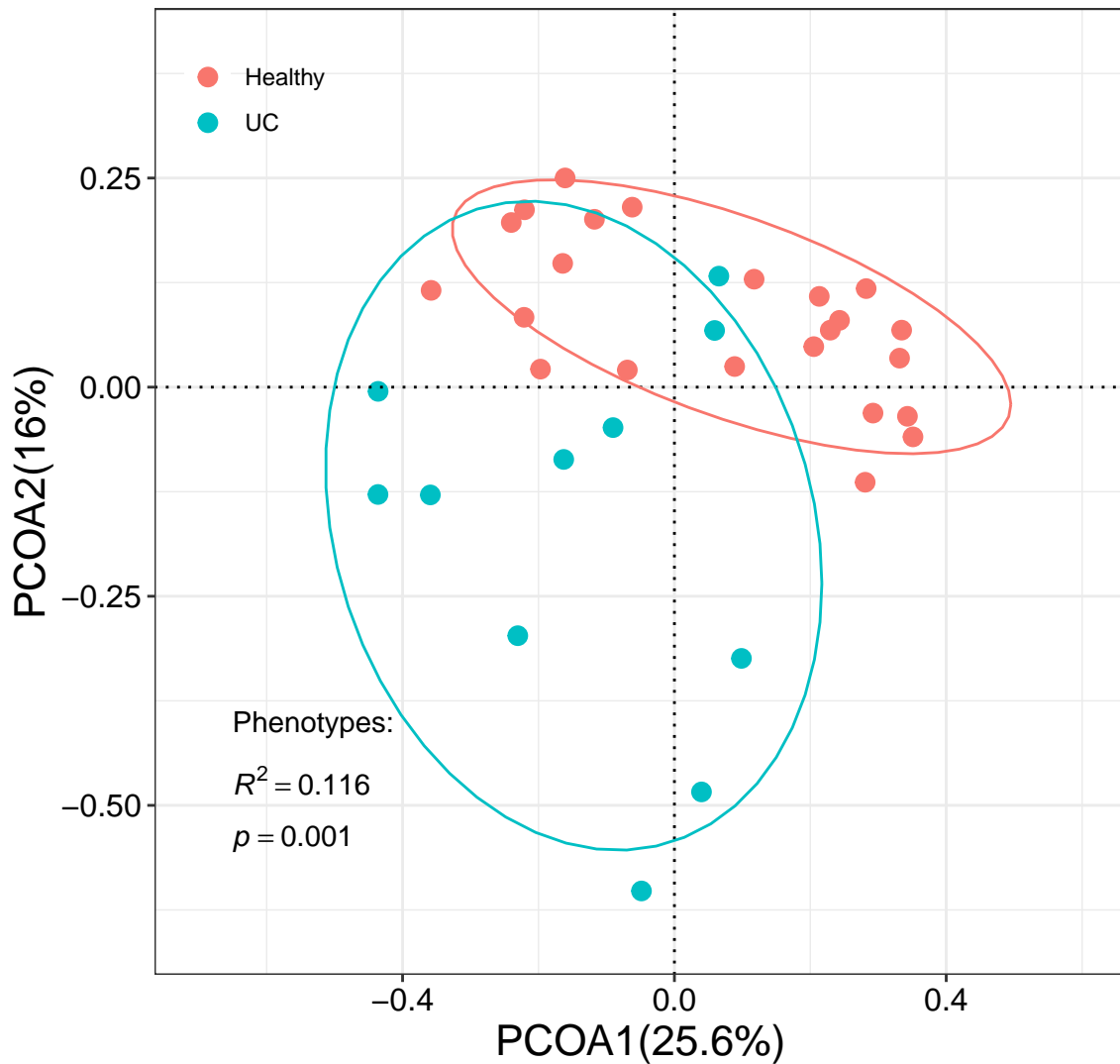

Supplement: Supplementary file 3 — Supplementary Information 3. [file 41598_2022_7995_MOESM3_ESM.zip › supplementary_tex/pcoa_remove_uc_therapy.pdf]

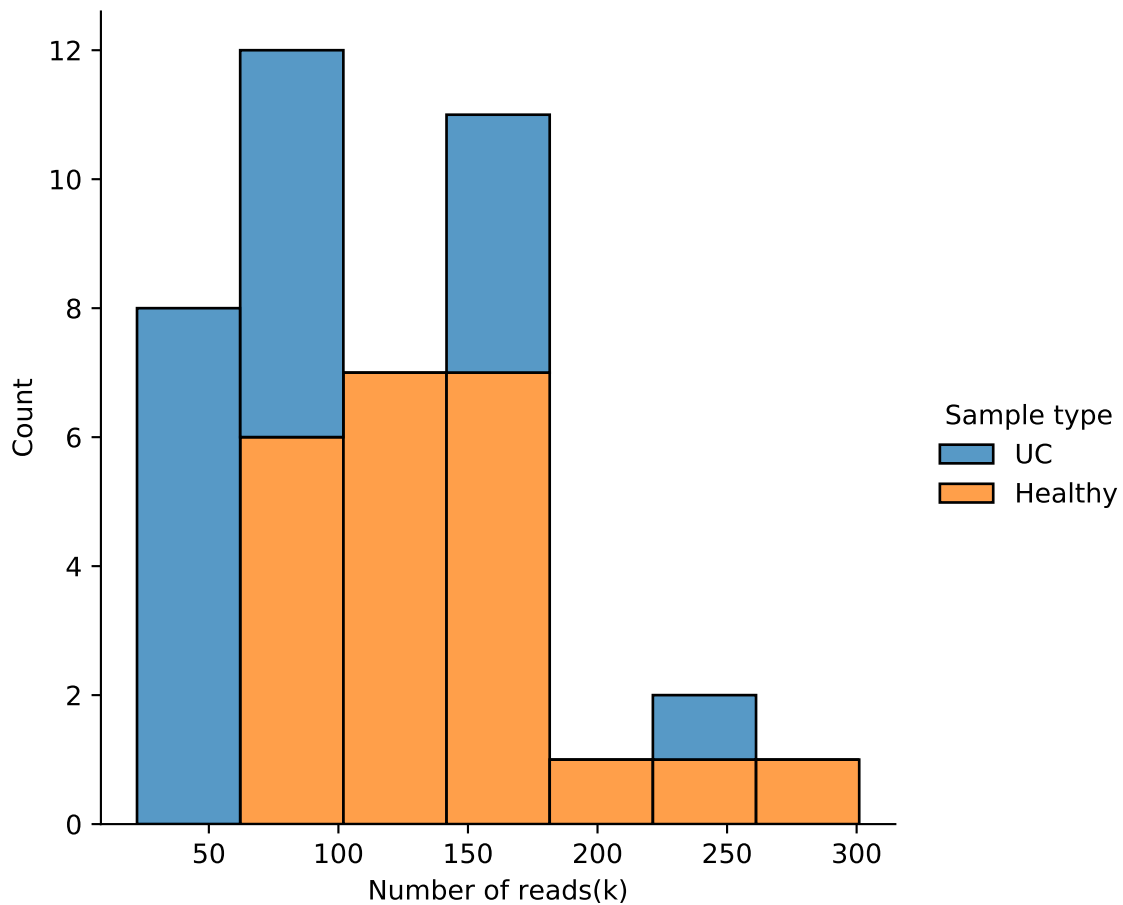

Supplement: Supplementary file 3 — Supplementary Information 3. [file 41598_2022_7995_MOESM3_ESM.zip › supplementary_tex/seq_depth_dist_16s.pdf]

16S genus Shannon Indices

Spearman's correlation test:

$\rho = 0.686$

$p = 1.34\text{e-}05$

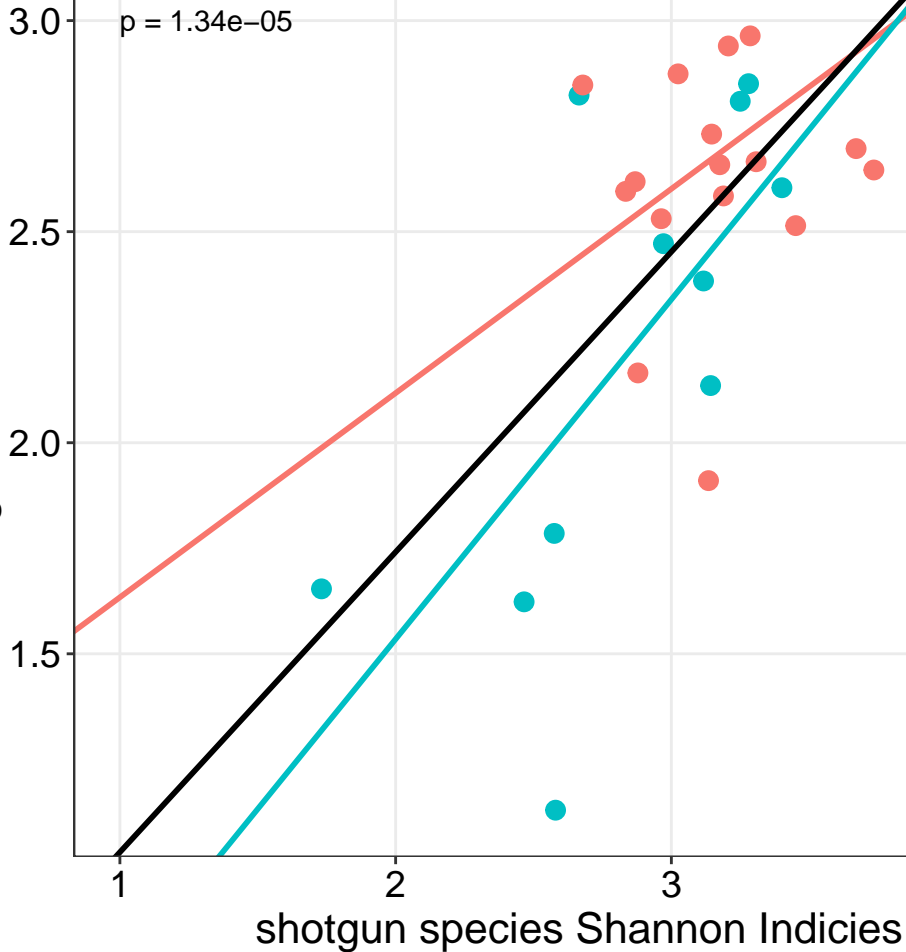

Healthy  
UC

Supplement: Supplementary file 3 — Supplementary Information 3. [file 41598_2022_7995_MOESM3_ESM.zip › supplementary_tex/corr_shannon_remove_uc_therapy.pdf]

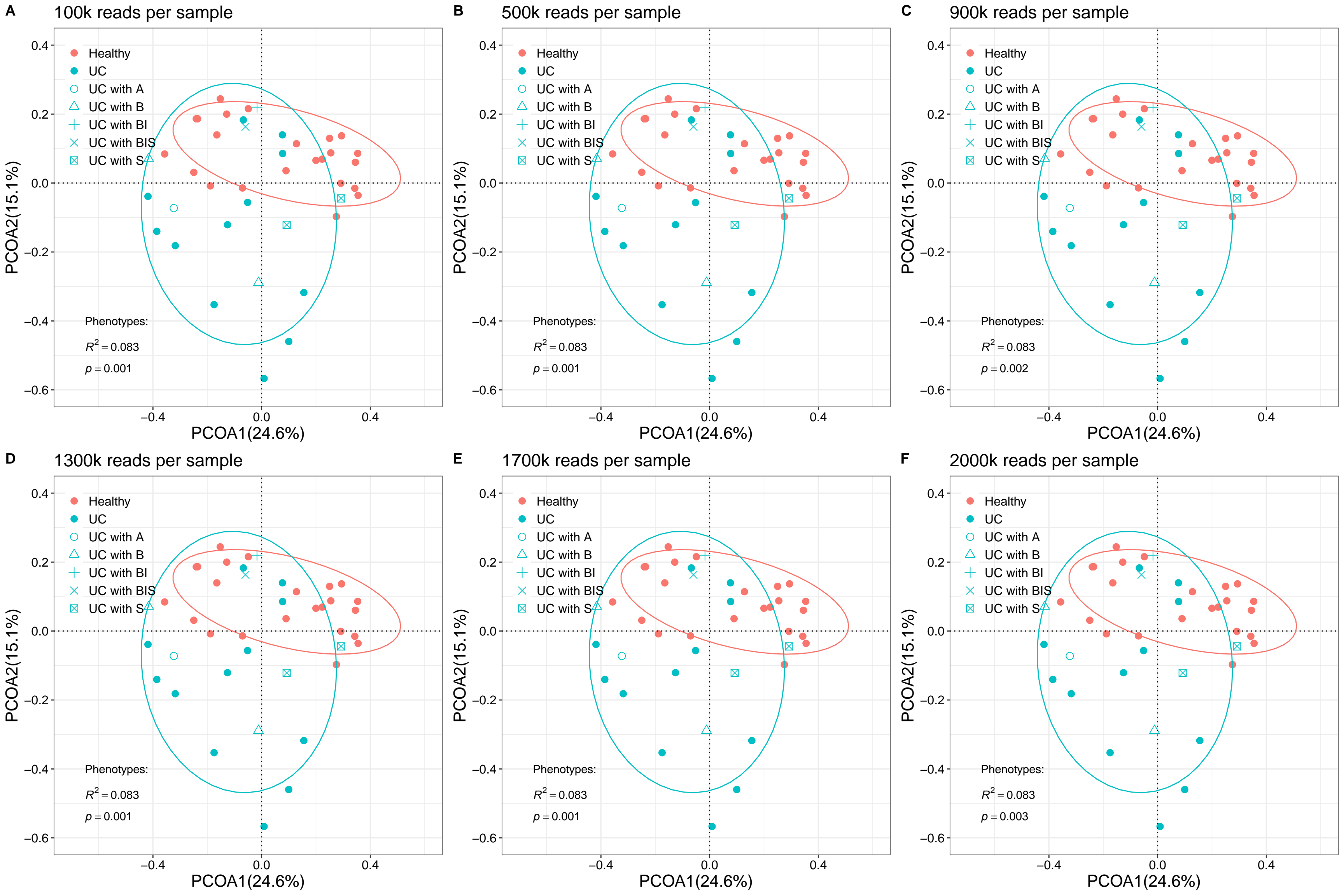

Supplement: Supplementary file 3 — Supplementary Information 3. [file 41598_2022_7995_MOESM3_ESM.zip › supplementary_tex/shotgun_rarefied_pcoa.pdf]

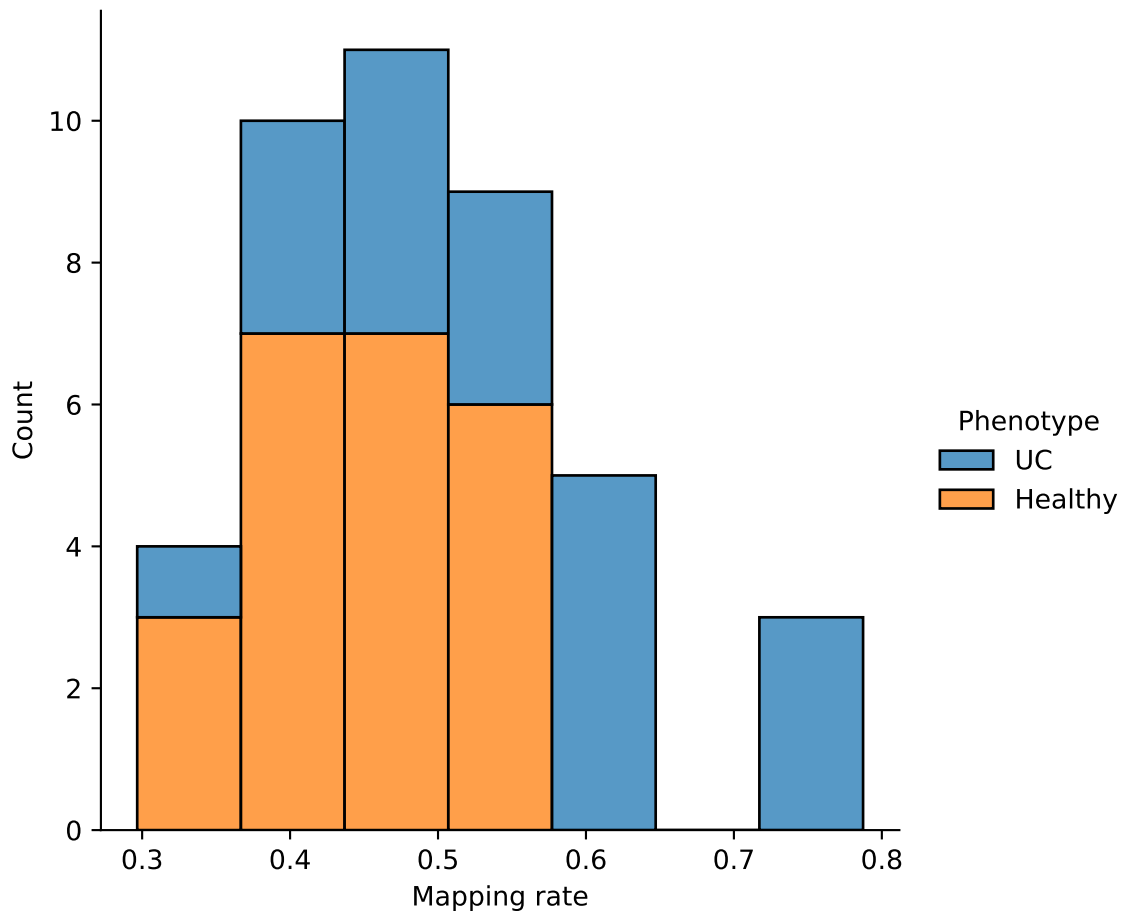

Supplement: Supplementary file 3 — Supplementary Information 3. [file 41598_2022_7995_MOESM3_ESM.zip › supplementary_tex/mapping_rate.pdf]

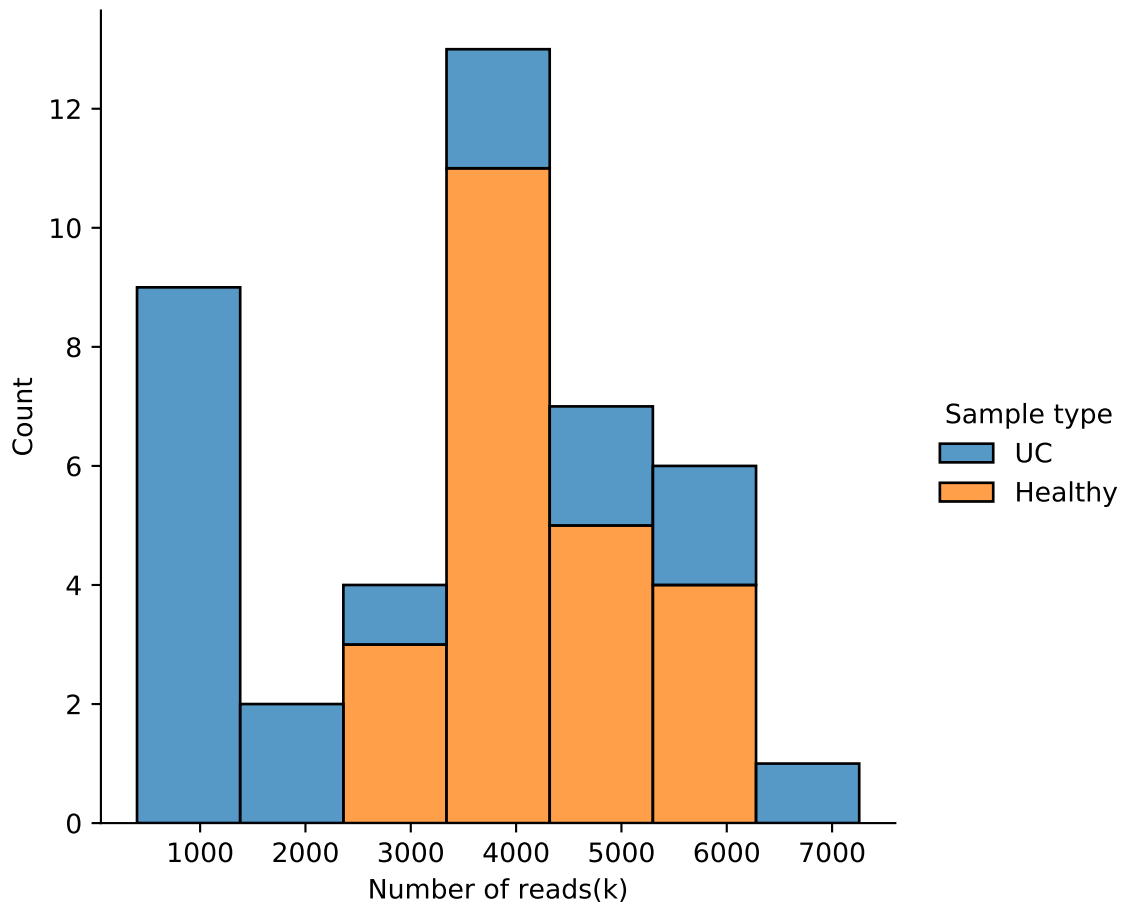

Supplement: Supplementary file 3 — Supplementary Information 3. [file 41598_2022_7995_MOESM3_ESM.zip › supplementary_tex/seq_depth_dist_sg.pdf]

**A** PCoA plot for 16S data

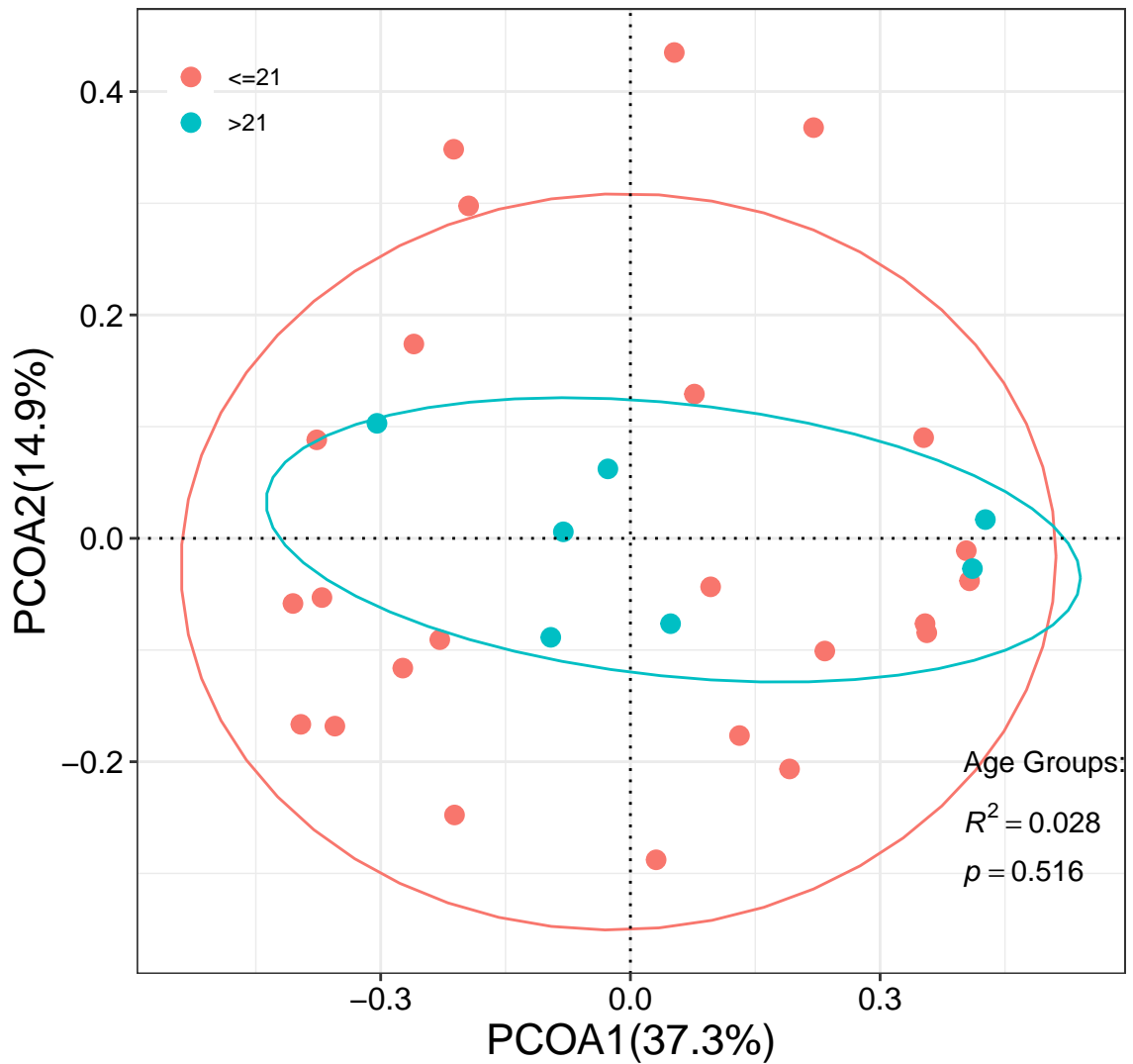

**B** PCoA plot for shotgun data

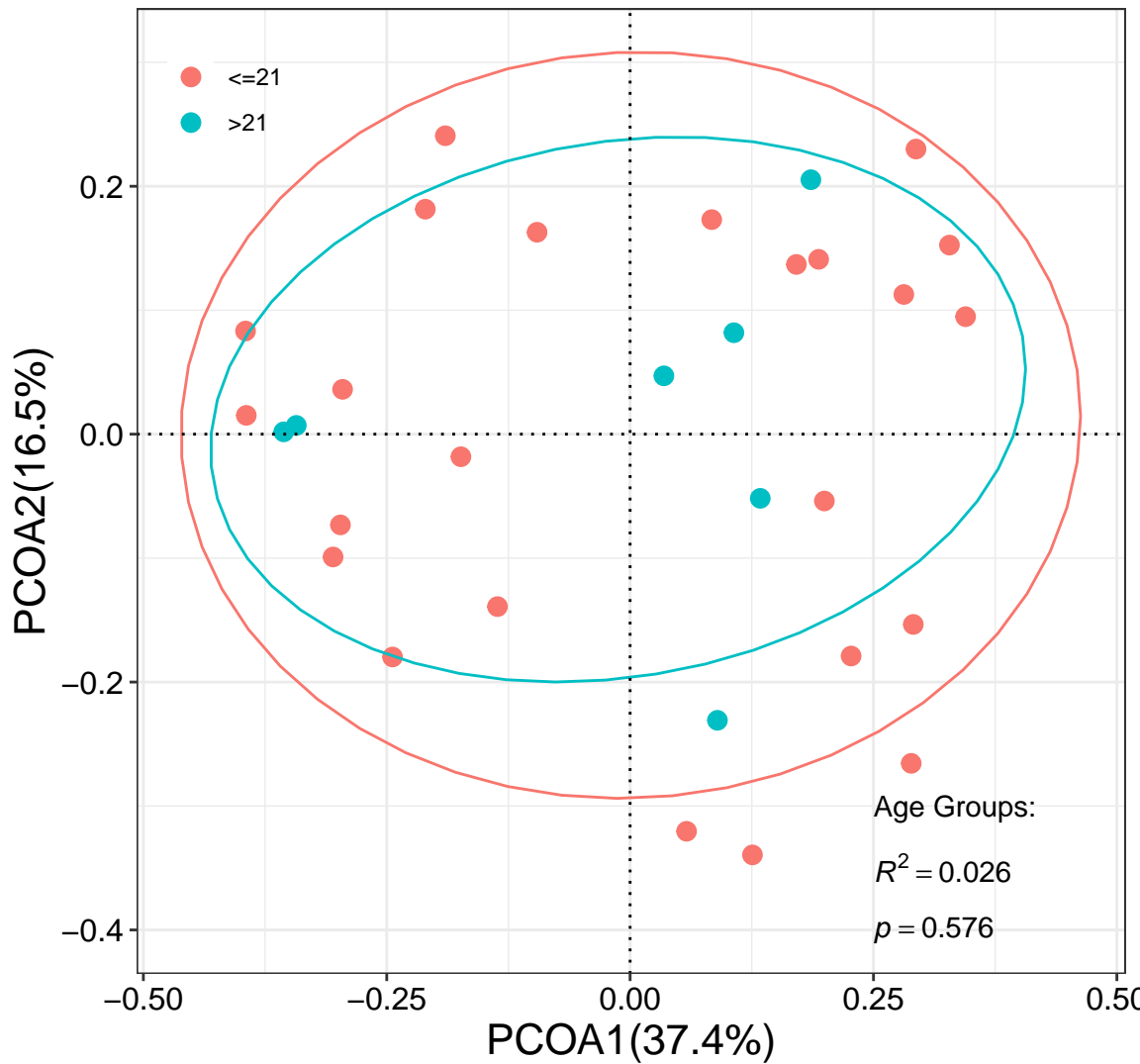

Supplement: Supplementary file 3 — Supplementary Information 3. [file 41598_2022_7995_MOESM3_ESM.zip › supplementary_tex/validation_healthy_pcoa.pdf]

16S genus Shannon Indices

Spearman's correlation test:  
 $\rho = 0.633$   
 $p = 1.11\text{e-}05$

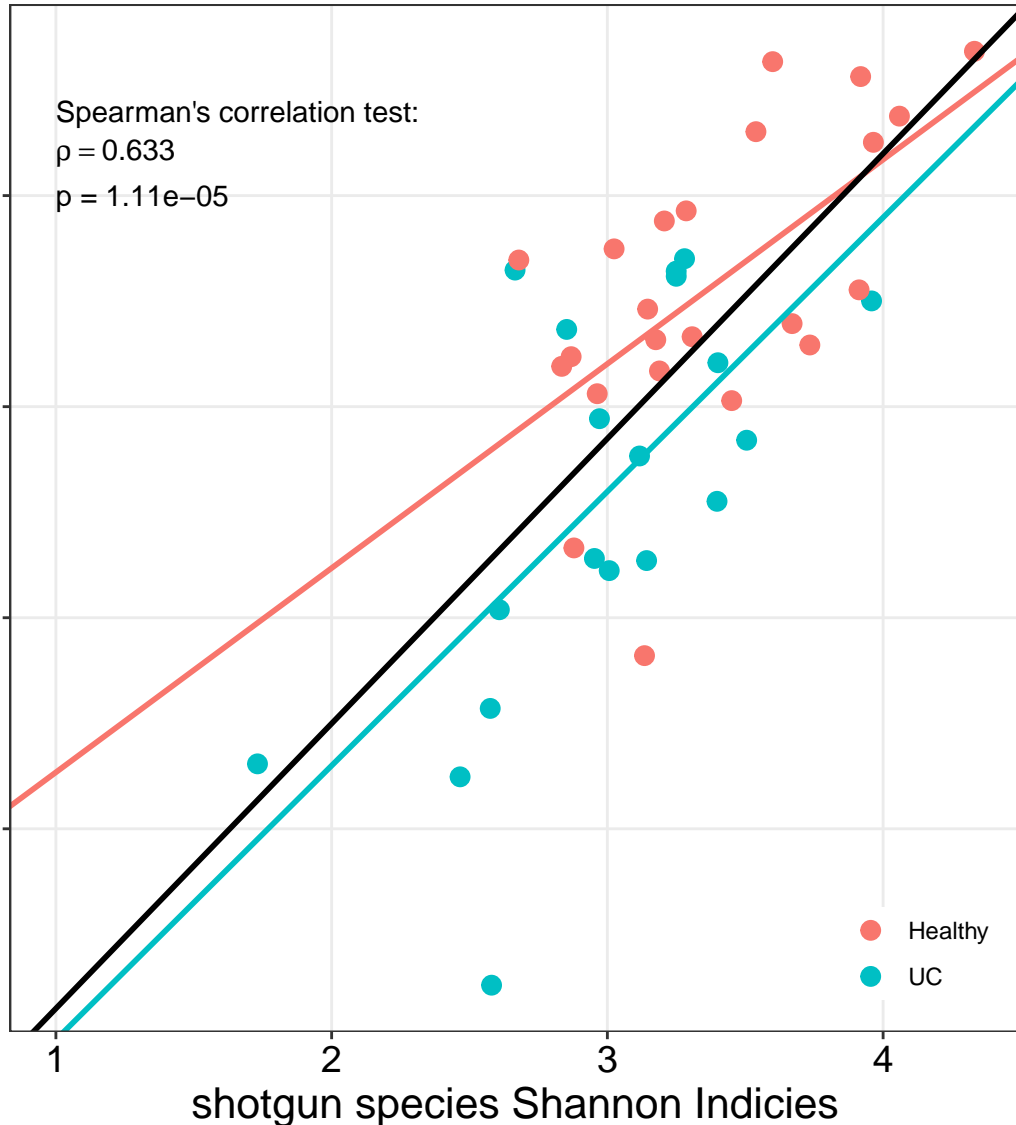

Supplement: Supplementary file 3 — Supplementary Information 3. [file 41598_2022_7995_MOESM3_ESM.zip › supplementary_tex/corr_shannon.pdf]

**A** Shannon Indices for 16S data

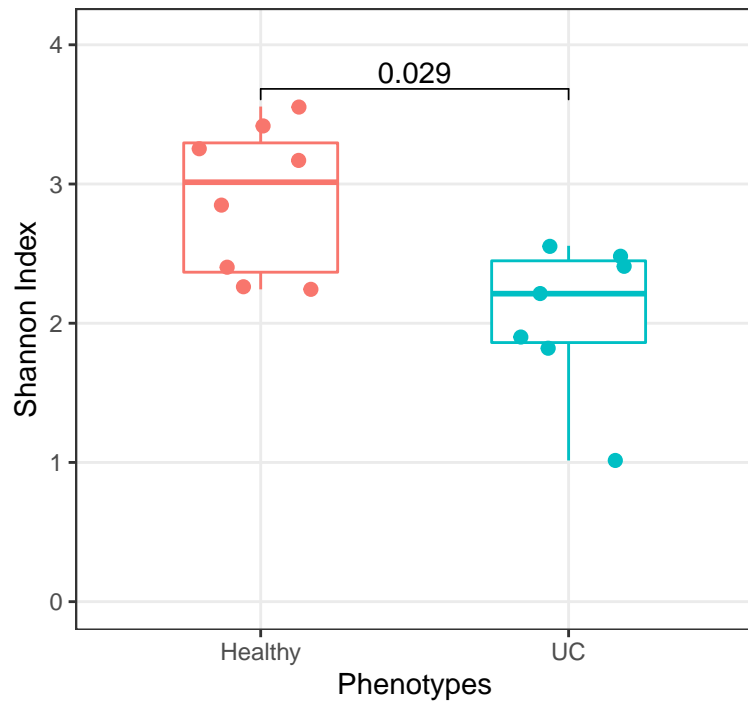

**B** Shannon Indices for shotgun data

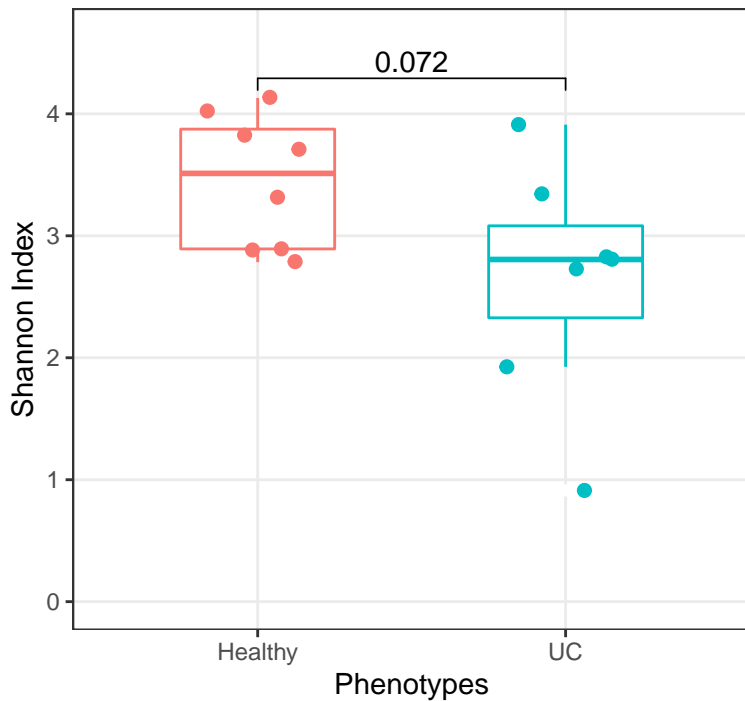

Supplement: Supplementary file 3 — Supplementary Information 3. [file 41598_2022_7995_MOESM3_ESM.zip › supplementary_tex/validation_shannon_boxplot.pdf]

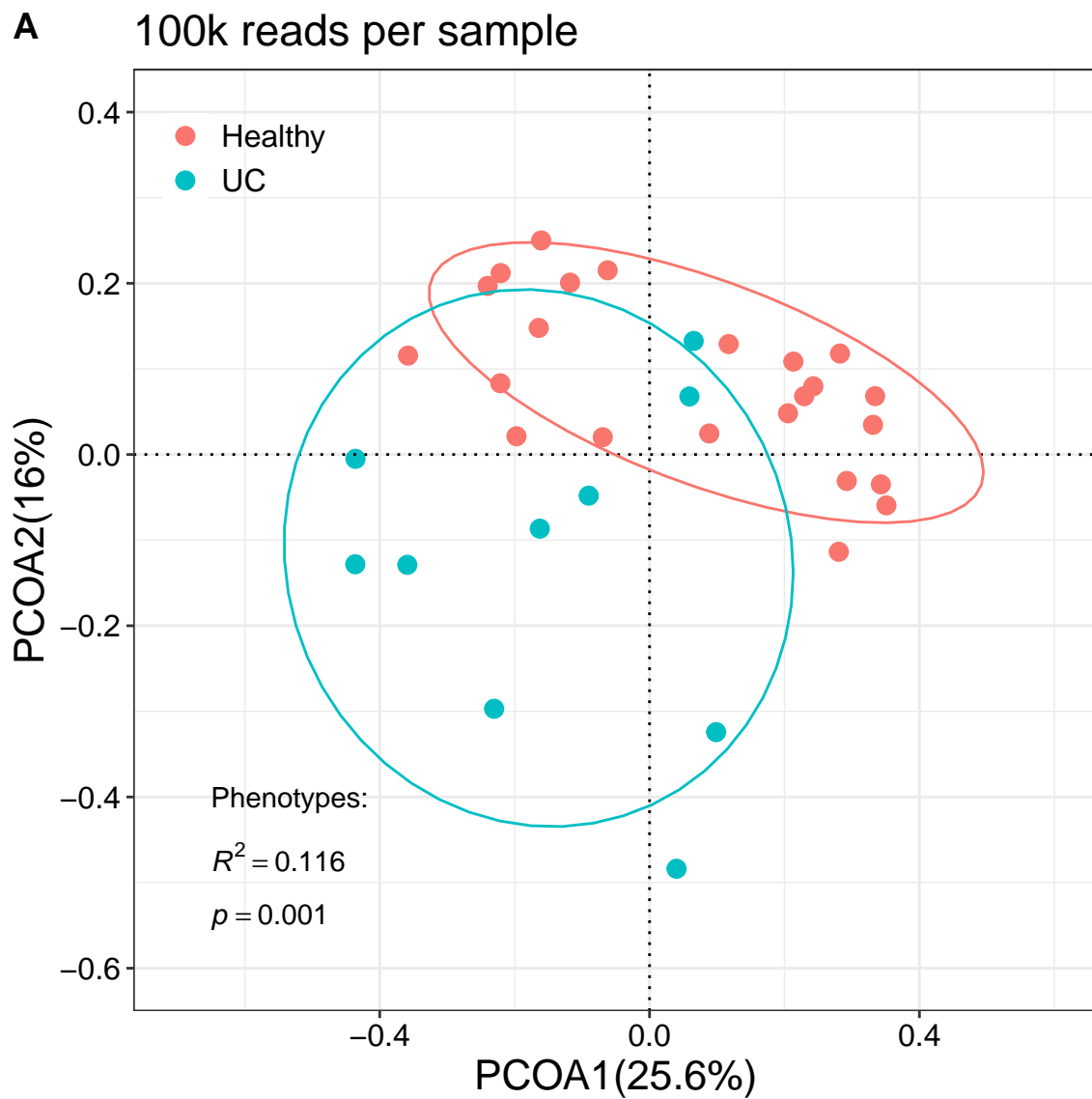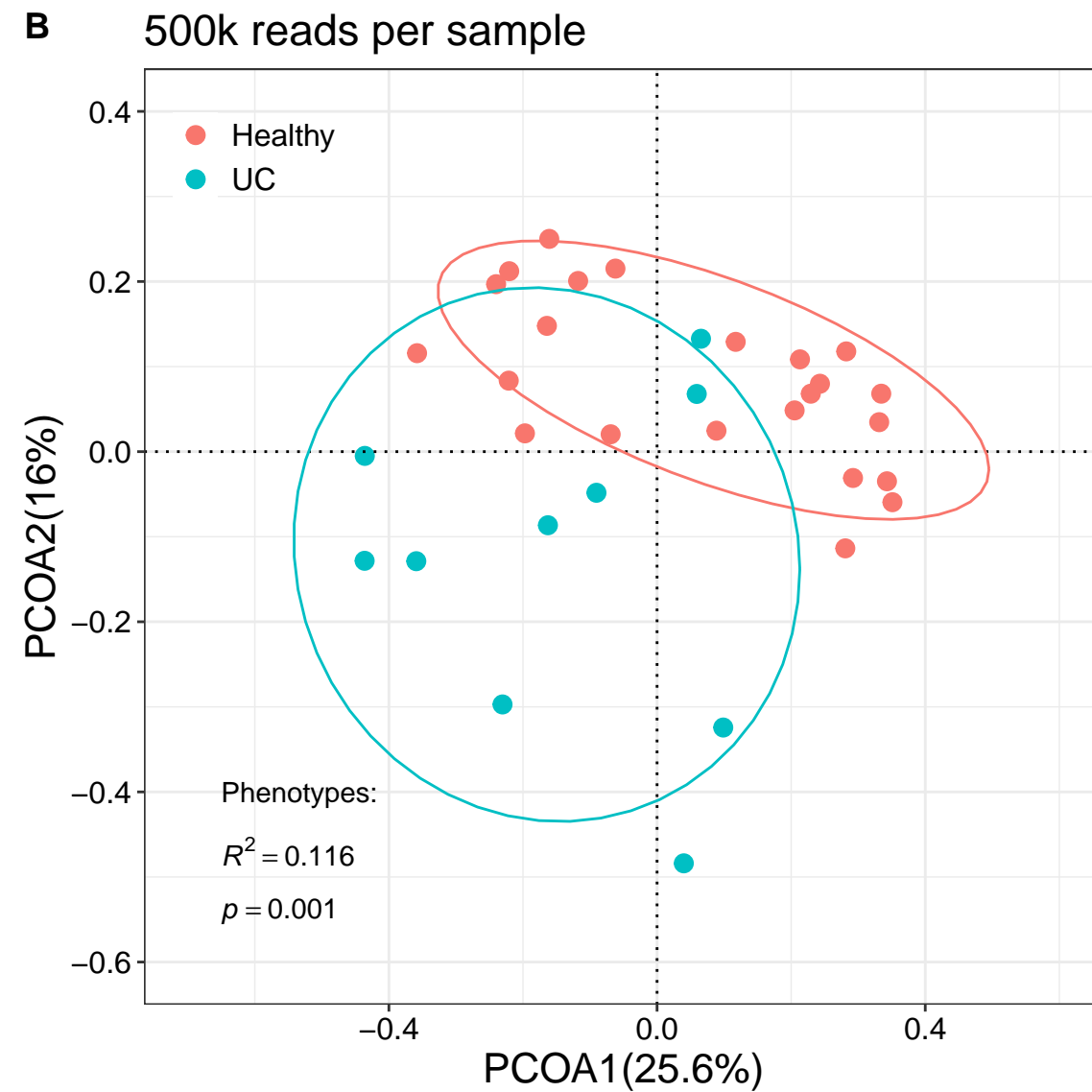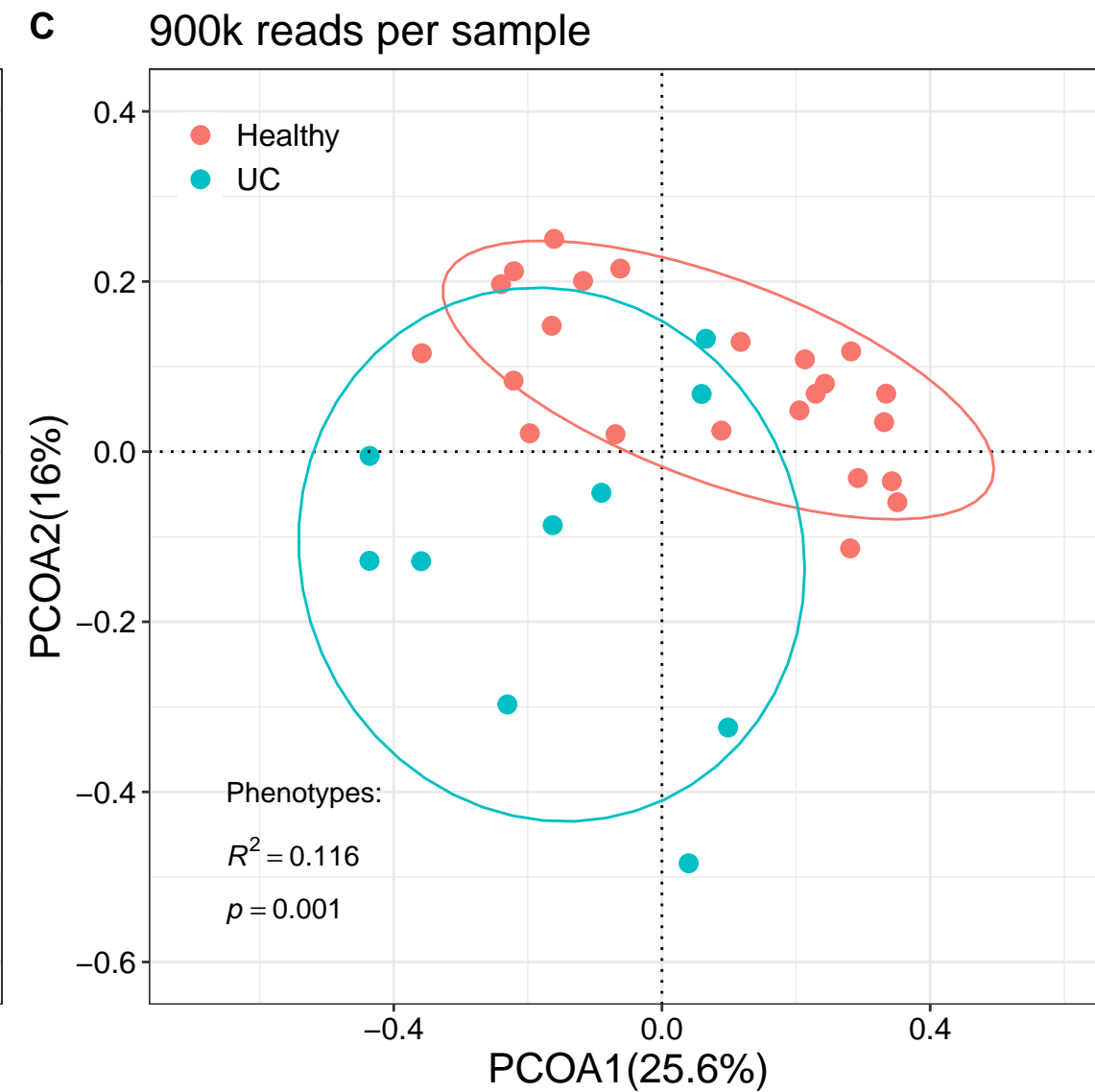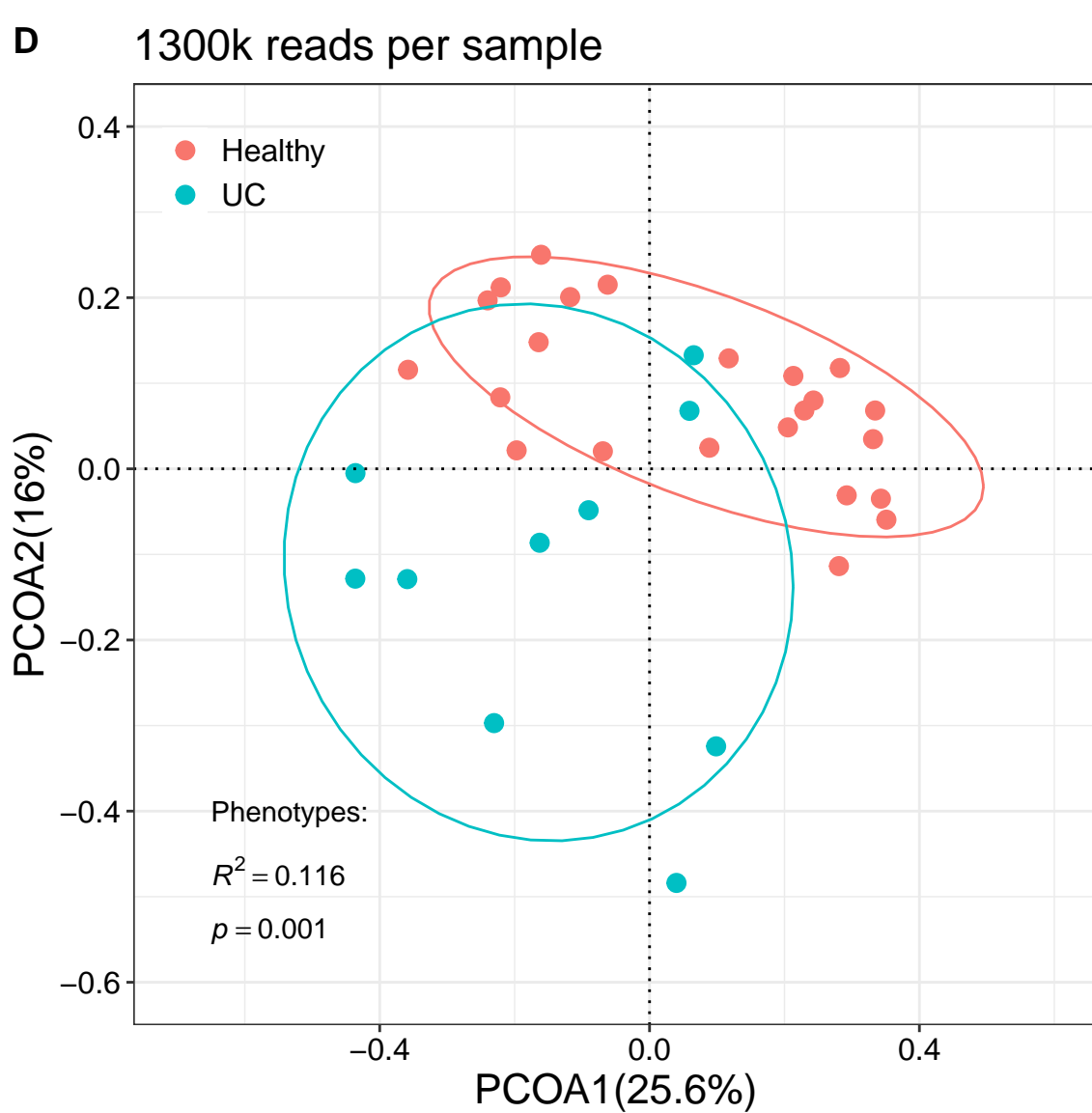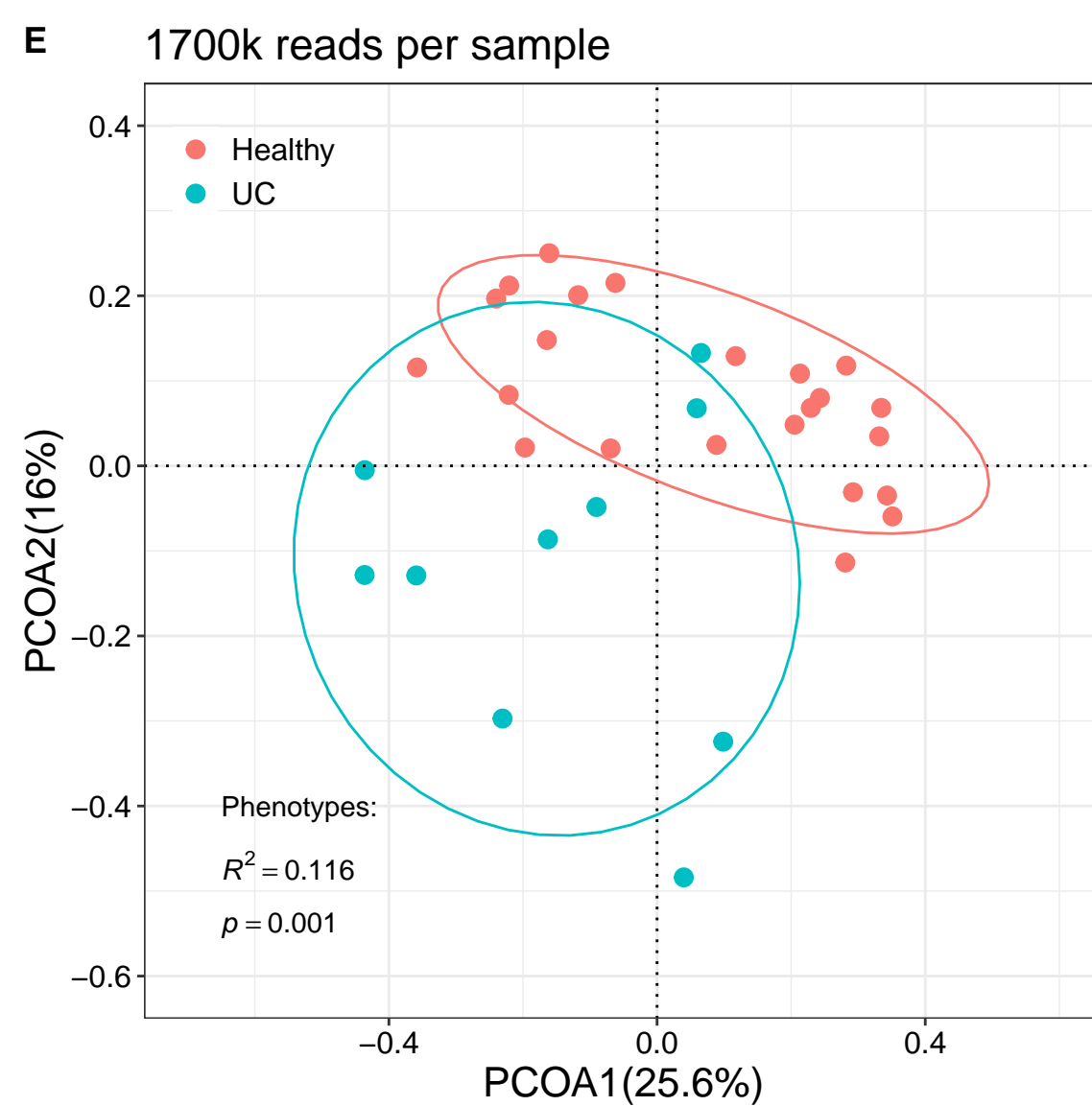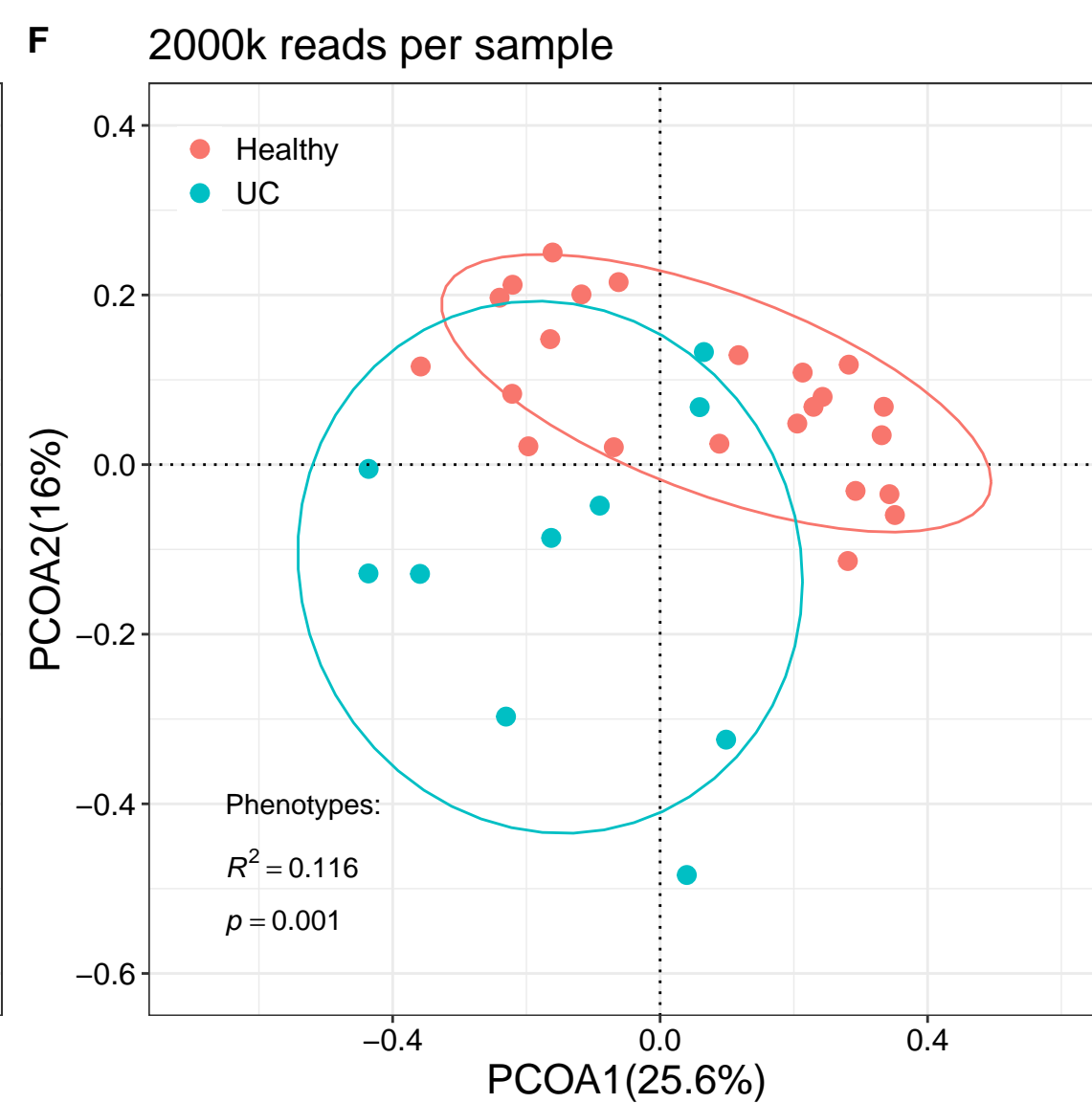

Supplement: Supplementary file 3 — Supplementary Information 3. [file 41598_2022_7995_MOESM3_ESM.zip › supplementary_tex/shotgun_rarefied_pcoa_remove_uc_therapy.pdf]

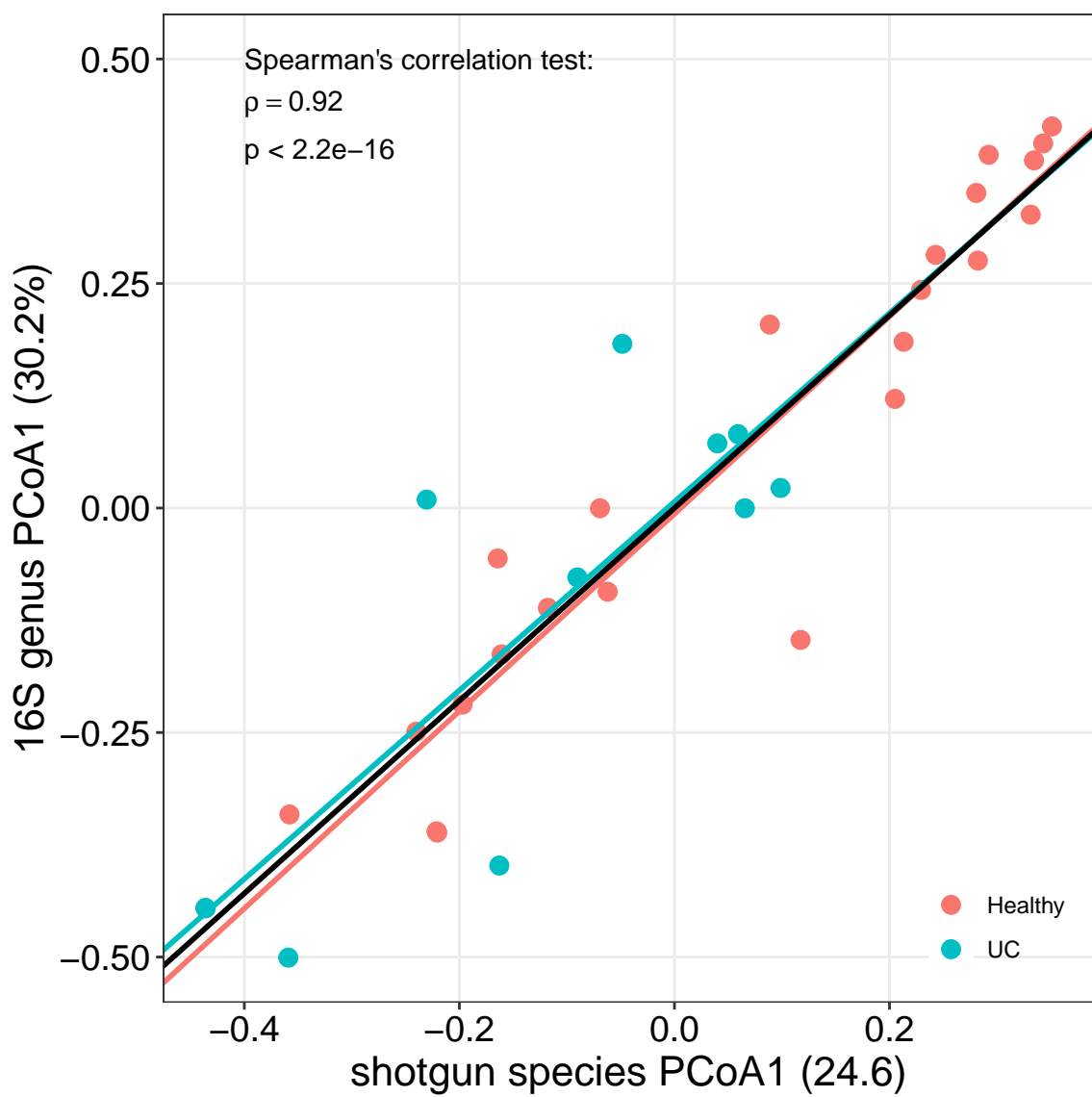

Supplement: Supplementary file 3 — Supplementary Information 3. [file 41598_2022_7995_MOESM3_ESM.zip › supplementary_tex/corr_pcoa1_remove_uc_therapy.pdf]
